# Limited Mitochondrial Permeabilization Causes DNA Damage and Genomic Instability in the Absence of Cell Death

**DOI:** 10.1016/j.molcel.2015.01.018

**Published:** 2015-03-05

**Authors:** Gabriel Ichim, Jonathan Lopez, Shafiq U. Ahmed, Nathiya Muthalagu, Evangelos Giampazolias, M. Eugenia Delgado, Martina Haller, Joel S. Riley, Susan M. Mason, Dimitris Athineos, Melissa J. Parsons, Bert van de Kooij, Lisa Bouchier-Hayes, Anthony J. Chalmers, Rogier W. Rooswinkel, Andrew Oberst, Karen Blyth, Markus Rehm, Daniel J. Murphy, Stephen W.G. Tait

**Affiliations:** 1 Cancer Research UK Beatson Institute, Garscube Estate, Switchback Road, Glasgow G61 1BD, UK; 2 Institute of Cancer Sciences, University of Glasgow, Garscube Estate, Switchback Road, Glasgow G61 1BD, UK; 3 Centre for Systems Medicine, Royal College of Surgeons in Ireland, Dublin 2, Ireland, Baylor College of Medicine, Houston, TX 77030, USA; 4 Center for Cell and Gene Therapy, Baylor College of Medicine, Houston, TX 77030, USA; 5 Department of Pediatrics-Hematology, Baylor College of Medicine, Houston, TX 77030, USA; 6 Division of Immunology, The Netherlands Cancer Institute, Plesmanlaan 121, Amsterdam 1066 CX, the Netherlands; 7 Department of Immunology, University of Washington, 750 Republican Street, Seattle, WA 98109, USA

## Abstract

During apoptosis, the mitochondrial outer membrane is permeabilized, leading to the release of cytochrome *c* that activates downstream caspases. Mitochondrial outer membrane permeabilization (MOMP) has historically been thought to occur synchronously and completely throughout a cell, leading to rapid caspase activation and apoptosis. Using a new imaging approach, we demonstrate that MOMP is not an all-or-nothing event. Rather, we find that a minority of mitochondria can undergo MOMP in a stress-regulated manner, a phenomenon we term “minority MOMP.” Crucially, minority MOMP leads to limited caspase activation, which is insufficient to trigger cell death. Instead, this caspase activity leads to DNA damage that, in turn, promotes genomic instability, cellular transformation, and tumorigenesis. Our data demonstrate that, in contrast to its well-established tumor suppressor function, apoptosis also has oncogenic potential that is regulated by the extent of MOMP. These findings have important implications for oncogenesis following either physiological or therapeutic engagement of apoptosis.

## Highlights

• MOMP can occur in a minority of mitochondria
• Minority MOMP triggers caspase activity but fails to kill cells
• Minority MOMP-induced caspase activity causes DNA damage and genomic instability
• Minority MOMP promotes cellular transformation and tumorigenesis

## Graphical Abstract

**Figure:**
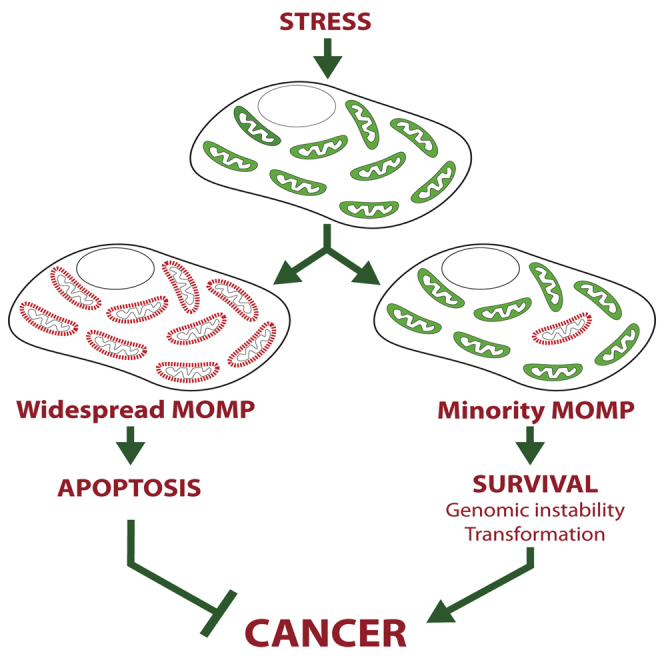

## Teaser

During apoptosis, mitochondrial outer membrane permeabilization (MOMP) is widespread, leading to rapid cell death. Here, Ichim et al. demonstrate that MOMP can also be engaged in a minority of mitochondria without killing the cell. Instead, minority MOMP triggers caspase-dependent DNA damage and genomic instability, thereby promoting transformation and tumorigenesis.

## Introduction

Following most apoptotic stimuli, the pro-apoptotic BCL-2 family members Bax and Bak permeabilize the outer membrane of the mitochondria, an event termed “mitochondrial outer membrane permeabilization” (MOMP). MOMP leads to rapid cell death by releasing mitochondrial proteins including cytochrome *c* that activate caspases (Tait and Green, 2010). However, even in the absence of caspase activity, cells typically die once MOMP has occurred, most likely due to progressive mitochondrial dysfunction (Lartigue et al., 2009; Tait et al., 2014). Due to these catastrophic effects, MOMP is often considered the point of no return in the apoptotic program. Mitochondrial apoptosis plays numerous important pathophysiological roles. In cancer, inhibition of apoptosis both promotes tumorigenesis and impedes anti-cancer therapeutic efficacy (Delbridge et al., 2012). Apoptotic inhibition is often achieved by upregulation of anti-apoptotic BCL-2 family members that prevent MOMP. This has led to the development of new anticancer drugs, called BH3-mimetics, which neutralize anti-apoptotic BCL-2 function (Ni Chonghaile and Letai, 2008).

Live-cell imaging has demonstrated that mitochondrial permeabilization is often an all-or-nothing event (Goldstein et al., 2000). Widespread mitochondrial permeabilization underpins the lethal effects of MOMP by ensuring robust caspase activity, or in its absence, massive mitochondrial dysfunction. In some limited circumstances, cells can survive MOMP. For example, growth factor-deprived neurons can survive MOMP due to a failure to properly engage caspase activity (Deshmukh and Johnson, 1998; Martinou et al., 1999; Wright et al., 2004). In proliferating cells, expression of the key glycolytic enzyme GAPDH can promote cell survival following MOMP provided caspase activity is inhibited (Colell et al., 2007). We have previously found that the ability of cells to survive MOMP depends on a few mitochondria that evade permeabilization and re-populate the cell (Tait et al., 2010).

Whereas earlier studies demonstrated that strong pro-apoptotic stimuli lead to rapid, synchronous, and complete MOMP, technical limitations have made it impossible to study the effects of sub-lethal stresses on individual mitochondria. Here, we use newly developed imaging techniques to demonstrate that MOMP can occur in a limited subset of mitochondria following a sub-lethal stress. Crucially, this limited MOMP leads to caspase activation, which, while insufficient to trigger cell death, leads to limited cleavage of key caspase substrates. This in turn drives DNA-damage and genomic instability, promoting transformation and tumorigenesis. Importantly, our data argue that the mitochondrial apoptotic pathway may exert either a tumor suppressor or oncogenic function depending upon the extent of MOMP.

## Results

### Limited Mitochondrial Permeabilization Occurs in the Absence of Cell Death

**Figure 1:**
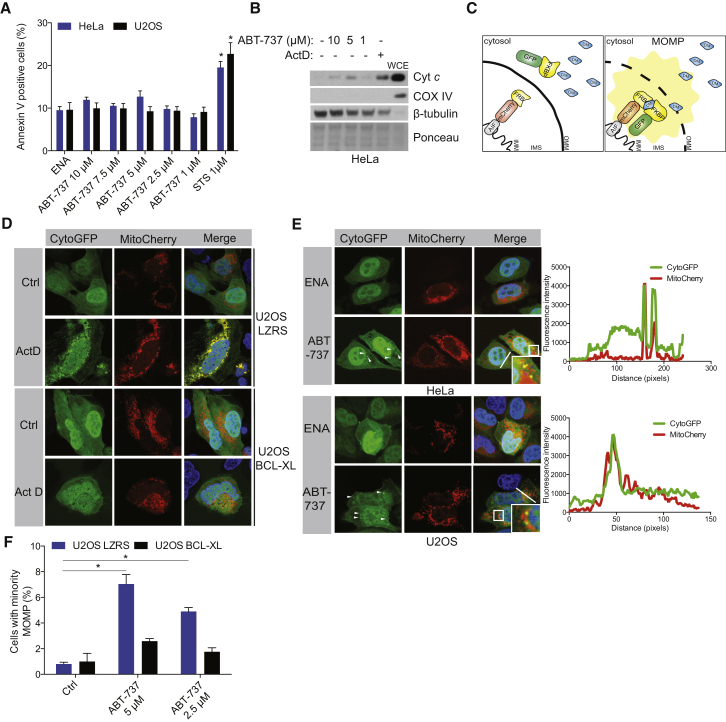
Limited Mitochondrial Outer Membrane Permeabilization Occurs without Triggering Cell Death (A) HeLa and U2OS cells were treated for 3 hr with different concentrations of ABT-737 or enantiomer (ENA, 10 μM) or with staurosporine (STS, 1 μM) for 12 hr and analyzed by flow cytometry for Annexin V-positive cells. Data represent mean ± SEM of three independent experiments. (B) HeLa cells were treated for 3 hr with different concentrations of ABT-737 or actinomycin D for 6 hr (Act D, 1 μM), and cytosolic extracts were western blotted for cytochrome *c*, COX IV, and β-tubulin. WCE, whole-cell extract. (C) Schematic representation of GFP relocalization-based MOMP detection method. DM, chemical heterodimerizer; IMM, inner mitochondrial membrane; IMS, intermembrane space; OMM, outer mitochondrial membrane. (D) U2OS cells expressing vector or BCL-xL together with CytoGFP/MitoCherry were treated with Act D (1 μM) for 3 hr in the presence of heterodimerizer and imaged by confocal microscopy. (E) HeLa or U2OS cells expressing CytoGFP/MitoCherry were treated with vehicle or ABT-737 (5 μM) or enantiomer (5 μM, ENA) for 3 hr and imaged by confocal microscopy. Arrows denote permeabilized mitochondria. Line scans represent variation in red and green fluorescence intensity along the denoted line. (F) U2OS cells expressing CytoGFP/MitoCherry were treated for 3 hr with ABT-737, and minority MOMP was quantified. Data represent mean ± SEM of three independent experiments. ^∗^p < 0.05, compared versus control. See also Figure S1 and Movies S1 and S2.

Mitochondrial permeabilization during apoptosis is widespread such that most or all mitochondria within a cell undergo MOMP; this effectively commits a cell to die. However, the potential for sub-lethal apoptotic stresses to engage MOMP in a limited number of mitochondria has not been tested. To investigate this, we used ABT-737, the prototypic BH3-mimetic compound that sensitizes to apoptosis by antagonizing anti-apoptotic BCL-2 family proteins (Oltersdorf et al., 2005). HeLa or U2OS cells were treated with varying concentrations of ABT-737, enantiomer (less-active stereoisomer of ABT-737) or the apoptosis-inducer staurosporine (STS) and analyzed for apoptosis by Annexin V staining and flow cytometry. Importantly, whereas STS triggered apoptosis, treatment with ABT-737 at varying doses failed to induce detectable apoptosis (Figure 1A). Similarly, live-cell imaging using the cell impermeable dye Sytox green also failed to reveal a cytotoxic effect of ABT-737 treatment (Figure S1A). Finally, BH3-mimetic treatment at the indicated doses had no effect on long-term cell survival as determined by clonogenic assay (Figure S1B). We next asked if mitochondrial permeabilization occurred following this non-lethal BH3-mimetic treatment. HeLa cells were treated with ABT-737 or, as a positive control, Actinomycin D (Act D) and cytosolic fractions were probed for the presence of cytochrome *c* to detect MOMP. As expected, Act D treatment led to MOMP as demonstrated by the detection of cytochrome *c* in the cytosolic extract (Figure 1B). Surprisingly, treatment with a non-lethal dose of ABT-737 also led to low, but detectable levels of MOMP, implying that MOMP could be engaged without killing the cell (Figure 1B).

In caspase-proficient cells, complete MOMP invariably represents a point of no return; we therefore reasoned that MOMP could only be non-lethal if it occurred in a minority of mitochondria (called hereafter minority MOMP). To test this possibility, we developed a new approach to specifically visualize permeabilized mitochondria using fluorescent protein re-localization and chemically dimerizable FKBP/FRB domains (Belshaw et al., 1996). Two fluorescent probes were constructed: a cytosolic probe comprising of GFP fused to a FKBP domain (cytoGFP) and a mitochondrial targeted probe (mitoCherry) comprising of mCherry fused to an FRB domain and the mitochondrial anchoring sequence of Apoptosis Inducing Factor (AIF) (Otera et al., 2005). In the presence of chemical heterodimerizer (A/C heterodimerizer, AP21967), the two probes can only co-localize on mitochondria following MOMP when cytoGFP can gain access to the mitochondrial inner membrane (Figure 1C). To validate this method, we treated U2OS cells with the apoptotic stimulus Act D. Importantly, Act D treatment led to robust mitochondrial re-localization of cytoGFP only in the presence of dimerizer that, as expected, was prevented by blocking MOMP by expression of BCL-xL (Figures 1D, S1C, and S1D). In line with this method marking MOMP as the key initiating apoptotic event, cytoGFP re-localization preceded caspase dependent apoptotic effects including cell shrinkage, rounding, and plasma membrane blebbing (Movie S1). Further verifying this technique, Act D-induced mitochondrial localization of GFP was only observed in cells in which MOMP had occurred, as demonstrated by the cytosolic release of Smac mCherry (a verified reporter of MOMP) (Figure S1E and Movie S2) (Tait et al., 2010). These fluorescent tools thus allow us to detect the permeabilization of individual mitochondria, and thereby assay for the presence of minority MOMP.

Using this approach, we investigated the extent of MOMP following sub-lethal apoptotic stimuli. Strikingly, following ABT-737 treatment, we were able detect MOMP in a limited number of mitochondria in both HeLa and U2OS cells (Figure 1E). Confirming their permeabilization, mitochondria with relocalized CytoGFP had also released Smac-mCherry and cytochrome *c* (Figures S1F and S1G). The percentage of cells displaying minority MOMP increased in a dose-dependent and BCL-xL inhibitable manner following ABT-737 treatment (Figure 1F). Importantly, using live-cell imaging, cells displaying minority MOMP failed to undergo cell death during extended periods of analysis (Figure S1H). Collectively, these data demonstrate that MOMP can occur in a limited number of mitochondria in response to ABT-737 treatment without leading to cell death.

### Minority MOMP Engages Sub-Lethal Caspase Activity

**Figure 2:**
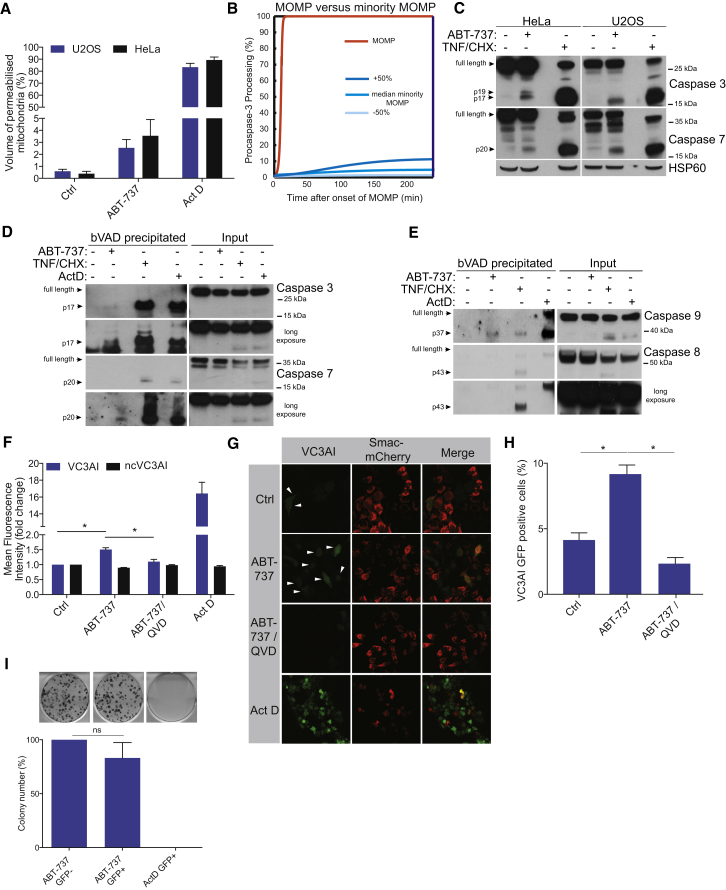
Minority MOMP Engages Sub-Lethal Caspase Activity (A) U2OS and HeLa cells expressing CytoGFP/MitoCherry were treated or not with ABT-737 (10 μM for 3 hr in the presence of dimerizer) to induce minority MOMP, and then z stack confocal imaging was performed. Act D (1 μM) was used to induce complete MOMP. Mitochondrial volume was measured using ImageJ. Data represent mean of permeabilized mitochondrial volume ± SEM from ten cells per condition. (B) Data from (A) were used as inputs into a mathematical HeLa cell model of apoptosis execution signaling to calculate the consequences of minority MOMP on the efficacy of procaspase-3 processing. (C) HeLa and U2OS cells were treated with ABT-737 (10 μM) or TNF/CHX (20 ng/ml TNF and 1 μg/ml CHX) for 3 hr, and cell extracts were western blotted for caspases-3 and -7. (D) Biotinylated-VAD-FMK (bVAD) was incubated with HeLa cells for 1 hr following 3 hr treatments with the indicated stimuli. Cell lysates and precipitated proteins were western blotted for caspases-3 and -7. (E) As in (D), except HeLa cells were pre-incubated for 1 hr with bVAD and treated for 3 hr with ABT-737 (10 μM) or 16 hr Act D (16 hr, 1 μM) or 3 hr TNF/CHX (20 ng/ml TNF and 1 μg/ml CHX). Proteins were precipitated with neutravidin agarose resin. Cell lysates and precipitated proteins were western blotted for caspases-8 and -9. (F) HeLa cells stably expressing the caspase activity reporter (VC3AI) or non-cleavable control (ncVC3AI) were treated for 24 hr with 10 μM ABT-737 in presence or absence of Q-VD-OPh (10 μM). GFP mean fluorescence intensity of the viable cells was quantified by flow cytometry. Results represent the fold increase in fluorescence over control. Data represent mean ± SEM of four independent experiments. (G) HeLa cells stably expressing VC3AI together with Smac-mCherry were treated as in (F) and imaged for GFP. Arrows denote caspase reporter (GFP)-positive cells. (H) Quantification of percentage of GFP-positive cells following ABT-737 (10 μM) treatment in the presence or absence of Q-VD-OPh (10 μM). Data represent mean ± SEM of three independent experiments. (I) HeLa VC3AI cells were treated with ABT-737 (10 μM) for 24 hr or Act D (0.5 μM), and equal numbers of GFP-positive cells (ABT-737 and Act D treated sample) and GFP-negative cells (ABT-737 treated cells) were sorted by flow cytometry and assessed for clonogenic survival. Data represent mean ± SEM of three independent experiments. ^∗^p < 0.05, compared versus control. See also Figure S2.

We next sought to understand the consequences of minority MOMP, and in particular, whether it might lead to activation of caspases at sub-apoptotic levels. We first quantified the extent of minority MOMP in HeLa cells. This revealed an average of 2.5% of a cell’s mitochondria undergoing permeabilization following sub-lethal ABT-737 treatment (Figure 2A). Together with previously published criteria, this allowed us to adapt a mathematical HeLa cell model of the apoptosis execution phase (Rehm et al., 2006) to perform in silico simulations of the consequences of minority MOMP on the efficiency of caspase-3 processing and activation. Importantly, despite the presence of amplifying feedback loops which ensure rapid and full caspase activation in response to regular MOMP, simulations for minority MOMP conditions demonstrate that caspase-3 would be processed and activated sub-maximally and therefore, potentially, at sub-lethal levels (Figures 2B and S2A). To experimentally verify this, U2OS and HeLa cells were treated with the BH3-mimetic ABT-737 in the presence or absence of caspase inhibitor quinolyl-valyl-O-methylaspartyl-[2,6-difluoro- phenoxy]-methyl ketone (Q-VD-OPh). Treatment with a wide range of sub-lethal doses of ABT-737 triggered caspase activity as evidenced by pro-caspase-3 processing and PARP cleavage, effects that were blocked by caspase inhibition (Figures S2B and S2C). We next compared caspase activity between conditions that engage minority MOMP and apoptotic conditions. Cells were treated with ABT-737 to induce minority MOMP or with TNF/CHX or Act D to engage apoptosis (Figures 2C–2E and S2D). We determined executioner caspase-3 and -7 activity, by detection of their active, cleaved fragment (Figure 2C) or by their activity-dependent precipitation using biotin-Val-Ala-Asp-Fluoromethyl Ketone (b-VAD) (Figure 2D). Both approaches demonstrated that caspase-3 and -7 activity was detectable but significantly less in ABT-737 treated cells undergoing minority MOMP compared with apoptotic cells (Figures 2C and 2D). Levels of active caspase-9, precipitated with b-VAD, were also detectable but significantly less in ABT-737 treated cells undergoing minority MOMP in comparison to apoptotic cells (Figure 2E). In line with ABT-737 activating the mitochondrial caspase pathway, ABT-737 treatment led to caspase-9 but not caspase-8 activation (Figure 2E). Collectively, these data argue that minority MOMP can engage sub-lethal caspase activity. To corroborate these findings, we used a recently developed caspase reporter protein (VC3AI) that fluoresces following caspase-mediated cleavage (Zhang et al., 2013). As validation, apoptotic treatments led to an increase in fluorescence in VC3AI expressing HeLa cells in a caspase-dependent manner, whereas cells expressing the non-cleavable control (ncVC3AI) remained non-fluorescent (Figure S2E). Significantly, flow cytometry analysis demonstrated that ABT-737 treatment led to a detectable increase in caspase activity in viable cells that was inhibited by the caspase inhibitor Q-VD-OPh, further supporting the hypothesis that minority MOMP triggers sub-lethal caspase activation (Figure 2F). We then treated HeLa cells co-expressing Smac-mCherry together with either VC3AI or ncVC3AI with the BH3-mimetic ABT-737. In line with the flow cytometry data, treatment with ABT-737 specifically increased the percentage of cells displaying weak caspase-dependent fluorescence (Figures 2G, 2H, and S2F). Importantly, cells exhibiting caspase-activity failed to display apoptotic, widespread MOMP because Smac-mCherry remained localized in the mitochondria (Figure 2G). We next determined if cells displaying sub-lethal caspase activity could survive long-term. HeLa cells expressing the VC3AI reporter were treated either with ABT-737 to engage minority MOMP dependent caspase activity or Act D to trigger mitochondria-dependent apoptosis. Equal numbers of caspase active (GFP-positive cells) from both conditions were sorted and compared for clonogenic survival versus caspase inactive, GFP-negative cells (Figures 2I and S2G). Importantly, similar clonogenic survival was observed comparing ABT-737 treated caspase active versus inactive cells; in contrast, stimulation of mitochondrial dependent apoptosis by Act D prevented clonogenic outgrowth (Figure 2I). Taken together, these data demonstrate that minority MOMP can engage low level caspase activity under which cells can survive.

### Minority MOMP Induces Caspase-Dependent DNA Damage

**Figure 3:**
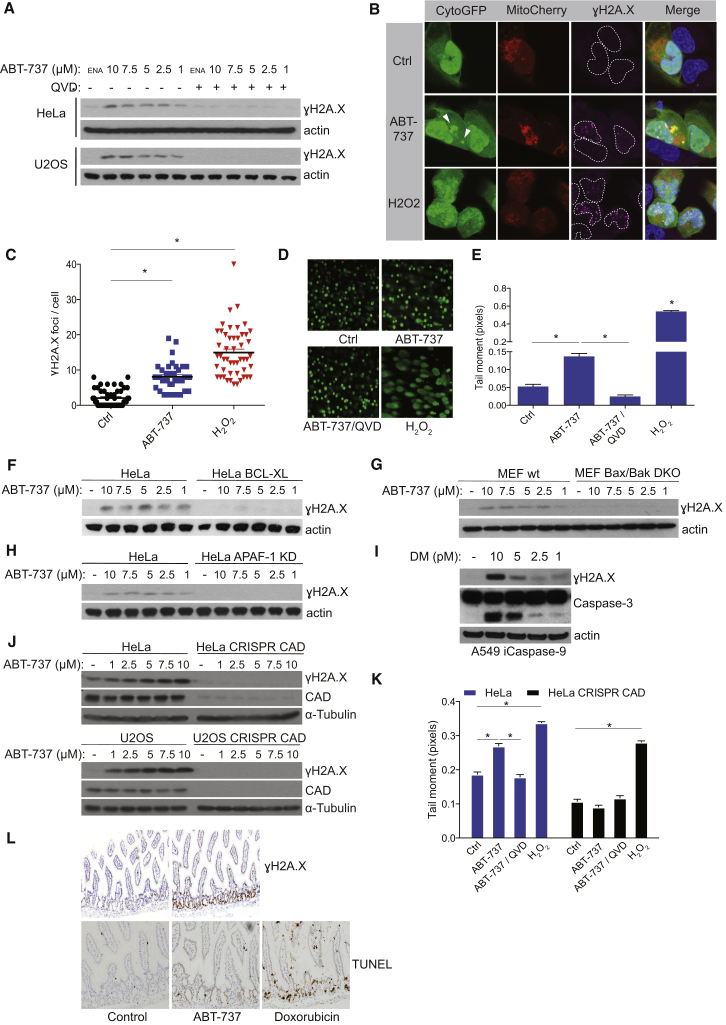
Minority MOMP Induces Caspase-Dependent DNA Damage (A) HeLa and U2OS cells were treated for 3 hr with indicated sub-lethal doses of ABT-737 or enantiomer (10 μM, ENA) in presence or absence of caspase inhibitor Q-VD-OPh (10 μM). Cell lysates were for probed by western blot for γH2A.X and actin (as loading control). (B) U2OS cells transiently expressing CytoGFP and MitoCherry were treated with ABT-737 (5 μM) for 3 hr or H_2_O_2_ (25 μM) for 10 min and immunostained for γH2A.X. Representative images are shown. (C) Quantification of γH2A.X foci in cells displaying minority MOMP (ABT-737-treated cells), control, and H_2_O_2_-treated cells. Data represent mean ± SEM of three independent experiments. (D) HeLa cells were treated as in (B) and subject to comet assay. Representative images are shown. (E) Quantification of comet tail moment following ABT-737 treatment. Data represent mean ± SEM of three independent experiments. (F–H) HeLa and HeLa overexpressing BCL-xL (F), wild-type MEF and MEF double knockout for Bax and Bak (G), or HeLa versus HeLa knockdown for APAF-1 (H) were treated as in (A) and western blotted for γH2A.X and actin. (I) A549 cells expressing caspase-9 fused to a FKBP dimerization domain were treated with indicated sub-lethal concentrations of homodimerizer (DM) for 3 hr to induce caspase-9 dimerization and activation. Cleavage of caspase-3 and γH2A.X was assessed by western blot. (J) Wild-type HeLa and U2OS cells and their *Cad*-deleted counterparts were treated and immunoblotted as in (A). (K) Wild-type and *Cad*-deleted HeLa cells were treated as in (D) and used to perform comet assay. Graph represents quantification of comet tail moment. Data represent mean ± SEM of four independent experiments. (L) Representative images of γH2A.X and TUNEL immunohistochemical staining in small intestine of mice treated with ABT-737 (75 mg/kg) for 1 day (n = 3). ^∗^p < 0.05, compared versus control. See also Figure S3.

DNA fragmentation is a classical apoptotic hallmark mediated by caspase-activated DNase (CAD) (Sakahira et al., 1998). We hypothesized that limited caspase activity following minority MOMP might lead to low-level CAD activation, and, in turn, to induction of DNA damage in surviving cells. To test this, we treated HeLa and U2OS cells with ABT-737 in the presence or absence of Q-VD-OPh and analyzed for γH2A.X as readout for DNA damage. Importantly, in both cell lines, non-lethal treatment with BH3 mimetic led to caspase-dependent DNA damage as demonstrated by a caspase-dependent increase in γH2A.X (Figure 3A). Importantly, the extent of DNA-damage (measured by γH2A.X foci) correlated with minority MOMP, implicating a causal relationship between the two (Figures 3B and 3C). The ability of BH3 mimetics to engage DNA-damage in a caspase-dependent manner was also observed in other cell lines (Figure S3A). Further demonstrating caspase-dependent DNA damage, ABT-737 treatment also led to an increase in DNA breaks, measured by comet assay and Ser^15^ p53 phosphorylation dependent on caspase function, mirroring γH2A.X levels (Figures 3D, 3E, and S3B). Although p53 independent, induction of DNA damage depended on mitochondrial caspase activation, because overexpression of BCL-xL (HeLa), deletion of Bax (HCT-116) or Bax and Bak in murine embryonic fibroblasts (MEF), or knockdown of APAF-1 (HeLa) prevented BH3 mimetic-induced γH2A.X (Figures 3F–3H and S3C–S3E). Supporting these findings, direct, non-lethal activation of caspase-9 by chemical dimerization also led to DNA damage (Figures 3I and S3F).

Because these data clearly demonstrate caspase-dependent activation of a DNA damage response, we next tested whether this effect is mediated by CAD activation. Caspase-3 activity is required for CAD activation through cleavage of its inhibitor ICAD. Consistent with caspase-3 and CAD-dependent DNA damage downstream of minority MOMP, γH2A.X was not observed following ABT-737 treatment of caspase-3 deficient MCF-7 cells in contrast to caspase-3 reconstituted MCF-7 (Figure S3G). To directly test the involvement of CAD in minority MOMP-induced DNA damage, we used CRISPR/Cas9 genome editing to generate CAD-deficient HeLa and U2OS cells (Figure 3J). In both cell lines, CAD deletion prevented BH3 mimetic induction of γH2A.X, demonstrating its requirement for minority MOMP-induced DNA damage (Figure 3J). Similar results were observed following CAD knockdown by siRNA (Figure S3H). In line with these findings, CAD deletion also effectively prevented ABT-737 induced DNA breaks, as determined by comet assay (Figure 3K). Furthermore, we also found that ICAD is cleaved following induction of minority MOMP, thereby supporting CAD activation (Figure S3I). Finally, we addressed if minority MOMP also promotes DNA damage in vivo. Mice were administered one dose of ABT-737 (either 75 mg/kg or 125 mg/kg) or administered daily with ABT-737 (75 mg/kg) over a 3-day period. Strikingly, a single dose of ABT-737 resulted in a significant increase of γH2A.X immunoreactivity in the small intestine of mice (Figure 3L). Repeated dosing did not result in an increase in γH2A.X immunoreactivity, potentially due to resolution of DNA damage in between doses (Figure S3J). Although not an absolute measure of apoptosis, because it detects cells only transiently during apoptosis, TUNEL staining failed to reveal any evidence of apoptosis following all ABT-737 treatments (Figures 3L and S3J). Collectively, these data show that sub-lethal stresses, causing minority MOMP, can trigger caspase-dependent DNA damage both in vitro and in vivo.

### Sub-Lethal BH3-Only and Apoptotic Stress Induce Minority MOMP and DNA Damage

**Figure 4:**
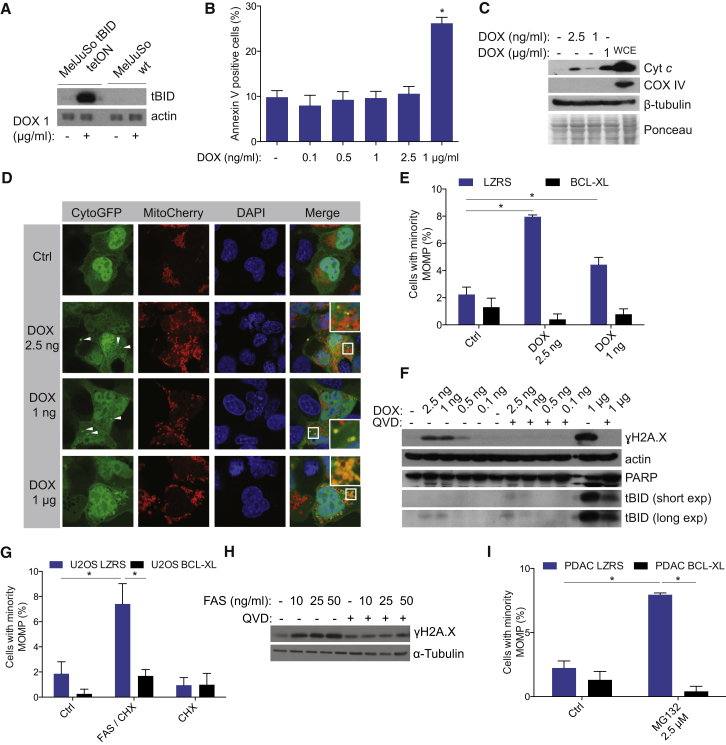
Sub-Lethal BH3-Only Protein and Apoptotic Stress Induces Minority MOMP and DNA Damage (A) MelJuSo tBID tetON or wild-type cells were treated for 12 hr with 1 μg/ml of doxycycline (DOX), and cell lysates were probed for tBID. (B) MelJuSo tBID tetON cells were treated for 6 hr with DOX and cell viability was assessed by Annexin V-based flow cytometry. Data represent mean ± SEM of three independent experiments. ^∗^p < 0.05, compared to control. (C) Cytosolic fractions from MelJuSo tetON tBID cells treated as in (B) were probed for cytochrome *c*. To induce apoptosis, 1 μg/ml DOX was used as a positive control. WCE, whole-cell extract. (D) MelJuSo tBID tetON expressing CytoGFP/MitoCherry were treated with DOX as in (A) and imaged by confocal microscopy. Arrows denote mitochondria undergoing permeabilization. (E) Quantification of cells undergoing minority MOMP. Data represent mean ± SEM of three independent experiments. (F) MelJuSo tBID tetON were treated with DOX as in (A) and cell lysates were probed by western blot for γH2A.X, PARP, and tBID. (G) U2OS cells stably expressing empty vector or BCL-xL were treated for 3 hr with FAS ligand (10 ng/ml) and CHX (1 μg/ml) and scored for the presence of minority MOMP. Data represent mean ± SEM of three independent experiments. (H) U2OS cells were treated for 3 hr with the indicated concentrations of FAS ligand and CHX (1 μg/ml), and western blot was performed for γH2A.X. (I) PDAC cells were treated with MG132 (2.5 μM) for 3 hr, and minority MOMP was quantified. Data represent mean ± SEM of three independent experiments. ^∗^p < 0.05, compared versus control. See also Figure S4.

Although our data demonstrate that the BH3 mimetic drug ABT-737 triggers minority MOMP and DNA damage, we sought to demonstrate that these effects also occur following MOMP triggered through other means. BH3-only proteins are the endogenous inducers of MOMP through their ability to activate Bax and Bak. Therefore, we next asked whether BH3-only proteins themselves could also engage minority MOMP. For this purpose, we generated a MelJuSo cell line expressing the BH3-only protein tBID under a doxycycline inducible promoter. Whereas doxycycline addition at 1 μg/ml led to robust tBID expression and apoptotic cell death, we were able to titrate doxycycline down to induce non-lethal tBID expression (Figures 4A, 4B, and S4A–S4D). We assessed whether expression of tBID at non-lethal levels could also trigger minority MOMP. Importantly, sub-lethal amounts of tBID (induced by 2.5 and 1 ng/ml doxycycline) led to minority MOMP as detected by the presence of cytochrome *c* in the cytosol (Figure 4C). We aimed to validate these findings using our method to detect MOMP via GFP re-localization. Non-lethal levels of tBID expression led to a clear increase in cells displaying minority MOMP in a manner that could be prevented through co-expression of BCL-xL (Figures 4D and 4E). Importantly, as was observed for BH3-mimetic treatment, the caspase inhibitor Q-VD-OPh also prevented H2A.X phosphorylation upon induction of sub-lethal levels of tBID (Figure 4F). We next addressed if a physiological apoptotic stimulus could also trigger minority MOMP and DNA-damage. Accordingly, sub-lethal treatment of U2OS cells with FAS ligand triggered minority MOMP and DNA-damage in a BCL-xL and caspase-inhibitable manner (Figures 4G, 4H, and S4E). Similarly, sub-lethal treatment of cells with the proteasome inhibitor MG132 also induced minority MOMP (Figure 4I). Collectively, these data demonstrate that similar to BH3-mimetics, apoptotic stimuli and pro-apoptotic BH3-only proteins also induce minority MOMP and DNA damage.

### JNK Regulates the DNA Damage Response

**Figure 5:**
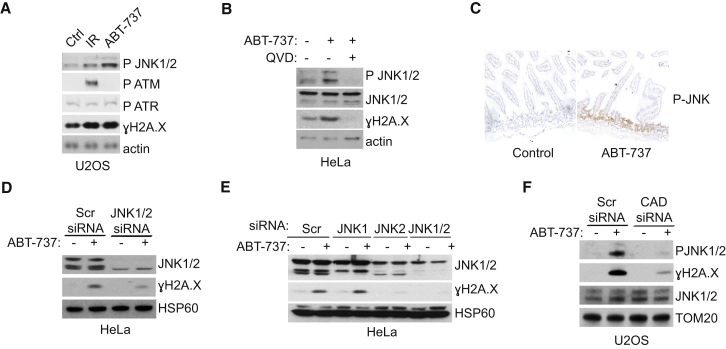
JNK Regulates the DNA Damage Response (A) U2OS cells were treated for 3 hr with 10 μM ABT-737, and phospho JNK1/2, ATM, and ATR were assessed by western blot. Ionizing radiation (2 Gy) was used as a positive control. (B) U2OS were treated with ABT-737 as in (A) in the presence or absence of Q-VD-OPh (10 μM) and immunoblotted for P-JNK1/2 and γH2A.X. (C) Representative images of P-JNK1/2 immunohistochemical staining in small intestine of mice treated with ABT-737 (75 mg/kg) for 1 day (n = 3); untreated mice (n = 3) were used as control. (D) HeLa cells were transiently transfected with siRNA for JNK1/2 and treated as in (A). Cell lysates were probed for total JNK1/2 and γH2A.X. (E) As in (D), except that siRNA oligos targeting JNK1, JNK2, or both JNK1 and JNK2 together were used. (F) U2OS cells were transfected with siRNA targeting CAD and treated with ABT-737. Cell lysates were probed for γH2A.X and P-JNK1/2. See also Figure S5.

DNA damage-induced phosphorylation of H2A.X at S139 often occurs via the PI3K-related kinase family members ATM, ATR, and DNA-PK (Jackson and Bartek, 2009; Shiloh, 2003). However, we did not observe any significant increase in activated, phosphorylated ATM or ATR kinase following sub-lethal ABT-737 treatment (Figure 5A). Moreover, RNAi-mediated knockdown of ATM, ATR, or DNA-PK failed to affect BH3 mimetic-induced γH2A.X levels (Figures S5A and S5B). Besides ATM, ATR, and DNA-PK, c-Jun N-terminal kinase (JNK) has also been found to mediate H2A.X phosphorylation in some settings (Lu et al., 2006). Importantly, sub-lethal treatment with ABT-737 led to a caspase-dependent increase in JNK1/2 activation mirroring levels of γH2A.X (Figure 5B). JNK activation following ABT-737 administration was also detected in vivo in the small intestine (Figure 5C). To directly investigate the role of JNK in H2A.X phosphorylation, we used RNAi. Combined knockdown of JNK1/2 or selective knockdown of JNK2 effectively prevented ABT-737 induced γH2A.X implicating a direct role for JNK2 in H2A.X phosphorylation (Figures 5D and 5E). Accordingly, RNAi-mediated knockdown of CAD largely inhibited JNK1/2 phosphorylation (Figure 5F). These results identify JNK2 as a key player in the minority MOMP-induced DNA damage response.

### Minority MOMP Promotes Genomic Instability

**Figure 6:**
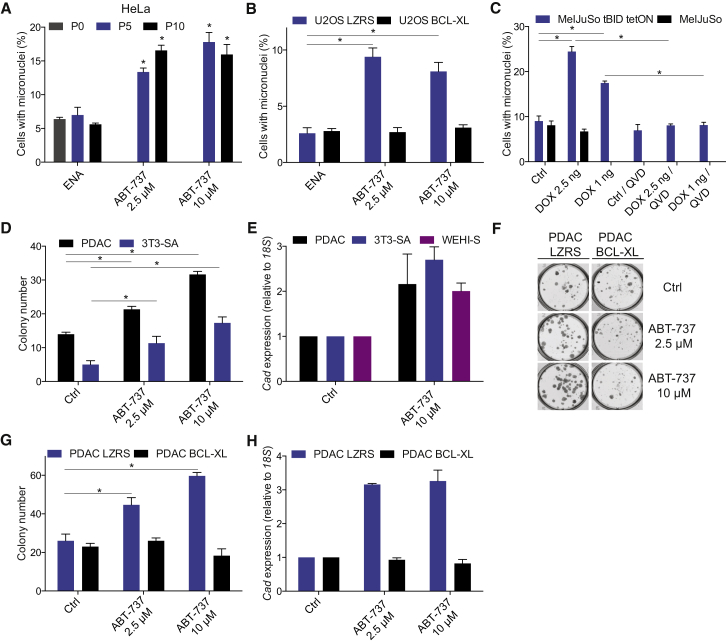
Minority MOMP Promotes Genomic Instability (A) HeLa cells were treated daily with ABT-737 or enantiomer (10 μM, Ctrl) at the indicated concentrations for either 5 (P5) or 10 (P10) passages and then scored for micronuclei. Data represent mean ± SEM of three independent experiments. (B) U2OS cells stably expressing BCL-xL (U2OS BCL-xL) or empty vector (U2OS LZRS) were treated as in (A) and assessed for micronuclei. Data represent mean ± SEM of three independent experiments. (C) MelJuSo tetON or wild-type MelJuSo cells were treated daily for ten passages with the indicated concentration of doxycycline (DOX) in the absence or presence of Q-VD-OPh, and micronuclei were scored. Data represent mean ± SEM of three independent experiments. (D) PDAC and 3T3-SA cells were treated daily for five passages with ABT-737 at the indicated concentrations, and clonogenic survival assay was performed in media containing PALA (100 μM). Data represent mean ± SEM of three independent experiments. (E) Genomic DNA was extracted from PALA-resistant PDAC, 3T3-SA, and WEHI-S clones, and *Cad* gene levels were quantified by qPCR. Data represent the mean ± SD from triplicate samples from a representative experiment carried out twice independently. (F) Representative images of PALA-resistant colonies from PDAC cells stably expressing BCL-xL (PDAC BCL-xL) or empty vector (PDAC LZRS). Cells were treated daily for five passages with ABT-737 at the indicated concentrations, and clonogenic survival assay was performed in media containing PALA (100 μM). (G) Quantification of PALA-resistance clonogenic survival in PDAC BCL-xL versus PDAC LZRS cells. Data represent mean ± SEM of three independent experiments. (H) *Cad* expression in PDAC BCL-xL and PDAC-LZRS PALA resistant colonies. Data represent the mean from a representative experiment carried out twice independently. Where stated, ^∗^p < 0.05, compared versus control. See also Figure S6.

Based on our results, we hypothesized that by causing DNA damage, minority MOMP may promote genomic instability and transformation. To test this possibility, we repeatedly treated HeLa and U2OS cells with sub-lethal doses of ABT-737 for five (P5) or ten passages (P10). Following blockage of cytokinesis, we then quantified the number of cells with micronuclei, a well-established marker for chromosomal damage (Figure S6A) (Fenech, 2007). Strikingly, U2OS and HeLa cells displayed a significant increase in micronuclei number following ABT-737 treatment in a dose-dependent manner (Figures 6A and S6B). Ectopic BCL-xL expression inhibited micronuclei accumulation in U2OS cells, confirming that the observed genomic instability required mitochondrial permeabilization (Figure 6B). In an analogous manner, induction of sub-lethal levels of the BH3-only protein tBID in MelJuSo cells also promoted micronuclei accumulation in a dose-dependent manner (Figure 6C). Micronuclei accumulation following tBID expression was reduced to control levels by the caspase inhibitor Q-VD-OPh, demonstrating a requirement for caspases in minority MOMP-induced chromosomal damage (Figure 6C).

DNA damage can also lead to gene amplification, a deleterious event that contributes to oncogene activation. We used the PALA assay to test if minority MOMP can trigger gene amplification (Mathew et al., 2009). PALA (N-phosphonoacetyl-L-aspartate) prevents pyrimidine synthesis by inhibiting the CAD enzyme (carbamyl phosphate synthetase/aspartate transcarbamylase/dihydro-orotase; note that this enzyme shares an abbreviation with, but is distinct from, caspase-activated DNase discussed previously). Resistance to PALA treatment in murine cells is mediated solely through *Cad* gene amplification. Various murine cell lines (PDAC, 3T3-SA, and WEHI-S) were repeatedly treated with BH3 mimetic, then assayed for amplification of the *Cad* locus and resistance to PALA treatment. Crucially, ABT-737 treatment promoted clonogenic survival in all tested murine cell lines following PALA treatment (Figures 6D and S6C), consistent with genome instability-driven *Cad* locus amplification. In line with this result, qPCR revealed that ABT-737 treatment induced *Cad* gene amplification in all three cell lines (Figure 6E). Importantly, BCL-xL overexpression in PDAC cells inhibited ABT-737-induced PALA resistance and *Cad* gene amplification, implicating a key role of minority MOMP in gene amplification (Figures 6F–6H, S6D, and S6E). Collectively, these results demonstrate that minority MOMP causes genomic instability.

### Minority MOMP Promotes Transformation and Tumorigenesis

**Figure 7:**
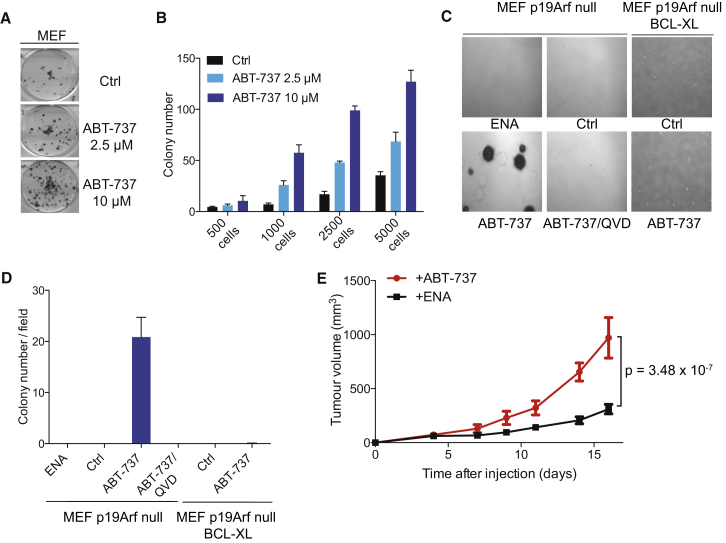
Minority MOMP Promotes Transformation and Tumorigenesis (A and B) Primary mouse embryonic fibroblasts (MEF) were treated daily for seven passages with the indicated concentrations of ABT-737 and transduced with E1A- and KRAS-expressing retrovirus. Representative images are shown in (A), and quantitation of the number of transformed colonies is depicted in (B). Data represent mean ± SEM of triplicate samples for a representative experiment (out of three independent experiments). (C) Primary p19^Arf^ null MEFs or p19^Arf^ null MEFs transduced with empty vector or BCL-xL-expressing retrovirus were treated for ten passages with ABT-737 (10 μM) in the presence or absence of Q-VD-OPh (10 μM), enantiomer (10 μM), or DMSO (Ctrl), and their anchorage-independent growth was assessed by soft agar assay. Representative images for each condition are shown. (D) Quantification of triplicate samples (mean ± SEM) from one representative soft agar assay (out of three independent experiments). (E) Primary p19^Arf^ null MEF were treated with ABT-737 (10 μM) or enantiomer (ENA, 10 μM) for ten passages. CD1-*Nude* female mice were injected subcutaneously with treated MEF and tumor growth was measured over time. Data are plotted as mean with error bars representing 95% CI (n = 15 for both treatments). Applying Welch’s t test to the area-under-the-curve data produces a p value of 3.48 × 10^−7^ for the tumor growth rates. See also Figure S7.

Genomic instability can promote oncogenic transformation. Therefore, we investigated if minority MOMP-induced DNA damage leading to genomic instability could potentiate E1A/KRAS-induced transformation in primary mouse embryonic fibroblasts (MEF). Primary MEFs were treated with sub-lethal doses of ABT-737 prior to transformation with E1A/KRAS. Remarkably, BH3-only stress was able to potentiate the transforming ability of E1A/KRAS in primary cells in a dose-dependent manner (Figures 7A and 7B). We next tested if minority MOMP could promote transformation in a different setting. Primary MEF deficient in the tumor suppressor p19^Arf^ were treated with enantiomer or ABT-737 in the presence or absence of caspase-inhibitor Q-VD-OPh (Figure S7A). Additionally, primary p19^Arf^ deficient MEF expressing BCL-xL or empty vector were treated with ABT-737 (Figures S7B and S7C). Following treatment, cellular transformation was assessed by anchorage-independent growth in soft agar. Strikingly, ABT-737 treatment led to transformation that was completely prevented by inhibiting caspases (with Q-VD-OPh) or by inhibiting MOMP through BCL-xL expression (Figures 7C and 7D). We next compared ABT-737 or enantiomer treated p19^Arf^ null MEF for their tumorigenic potential in vivo following subcutaneous injection in CD1-*Nude* mice. Significantly, ABT-737 treated MEF formed tumors much more rapidly than enantiomer-treated MEF (Figure 7E). Collectively, these results demonstrate that minority MOMP promotes both cellular transformation and tumorigenesis.

## Discussion

Apoptosis plays multiple beneficial roles in cancer, acting as both a potent tumor suppressor and therapeutic effector mechanism. Conversely, various reports also suggest an oncogenic function for apoptosis in some settings. For example, overexpression of anti-apoptotic BCL-2 proteins confers a favorable prognosis in certain cancer types whereas genetic deletion of the pro-apoptotic BH3-only proteins BID and PUMA inhibits tumorigenesis in some settings (Berardo et al., 1998; Biswas et al., 2013; Labi et al., 2010; Michalak et al., 2010; Reed, 1996). Moreover, caspase-dependent DNA damage has been described following mitotic slippage and treatment with the death-receptor ligand TRAIL (Lovric and Hawkins, 2010; Orth et al., 2012). Finally, following ethanol exposure, recovery from apoptosis is associated with genomic instability and transformation, although it remains unclear whether apoptosis has a direct causative role in this setting (Tang et al., 2012).

Our data demonstrate that by limited mitochondrial permeabilization, the same core apoptotic machinery that protects from cancer can now lead to caspase-dependent genomic instability and promote transformation. We find that the ability of minority MOMP to stimulate DNA damage requires mitochondrial-dependent caspase activation of the DNase CAD. Our finding that repeated engagement of minority MOMP triggers genomic instability may be due to an excessive induction of DNA damage and/or additionally a simultaneous inactivation of key DNA damage response (DDR) pathways. Accordingly, we observe sub-lethal caspase activation suffices to cleave PARP1, an important player in the DDR, as well as ATM and KAP1 (data not shown) both of have been shown or are predicted to be inactivated following caspase-mediated cleavage (Smith et al., 1999; Van Damme et al., 2005). Inactivation of ATM may also explain the requirement for JNK in mediating γH2A.X phosphorylation following CAD activity. Potentially, inactivation of additional DDR pathways may exacerbate DNA-damage and genomic instability initiated by minority MOMP.

Minority MOMP-induced DNA damage may play various important roles in cancer. For example, physiological triggers of mitochondrial apoptosis that fail to trigger cell death could promote genomic instability and cancer. Further studies investigating the impact of CAD deletion upon transformation and tumorigenesis will help shed light on this. The ability of anti-cancer therapies to trigger minority MOMP-induced DNA damage may have negative consequences. We find that the BH3 mimetic ABT-737 can induce DNA damage and genomic instability at doses comparable to those achieved during clinical application of BH3 mimetics. In combination with oncogenic stress or tumor suppressor loss, ABT-737 treatment promoted transformation. In cancer therapy, this raises a cautionary paradox, namely that treating tumor cells with an apoptosis-inducing therapy (such as BH3-mimetics) may in itself be oncogenic. Supporting this, etoposide-induced chromosomal translocations driving therapy-associated leukemia have previously been shown to require caspase and CAD activity; this possibly requires minority MOMP to initiate caspase activation (Hars et al., 2006; Sim and Liu, 2001). Second, minority MOMP-induced DNA damage that is engaged by pro-apoptotic anti-cancer therapies has the potential to be mutagenic. This could contribute to increased aggressiveness of relapsing tumors following treatment and to the acquisition of drug resistance. Indeed, acquired resistance to the BH3 mimetic ABT-199 was recently shown to be due to ABT-199-induced missense mutations in *BCL-2* (Fresquet et al., 2014).

Cell survival following minority MOMP may be even more important in post-mitotic, non-renewable cells such as neurons. Recently, neurons have been found to rapidly degrade cytochrome *c* following MOMP in a manner dependent on the E3 ubiquitin-ligase PARC (Gama et al., 2014). Beyond promoting cell survival, degradation of cytochrome *c* also has the potential to limit minority MOMP-dependent DNA damage. Additionally, removal of individual, permeablized mitochondria, particularly under stressful conditions, may also affect the DNA-damaging effects of minority MOMP. Along these lines, different studies have found mitochondria are degraded in an autophagy-dependent manner following permeabilization (Colell et al., 2007; Xue et al., 2001).

A variety of non-apoptotic roles for mitochondrial dependent caspase activation have been described, particularly in neurons (Hyman and Yuan, 2012). These include caspase-dependent dendrite pruning and AMPA receptor internalization (Li et al., 2010; Simon et al., 2012). Minority MOMP could be a potential mechanism to establish sub-lethal caspase activity in such processes.

Besides the detrimental, oncogenic effects of minority MOMP, our findings also raise potentially important therapeutic possibilities. For example, manipulating minority MOMP to convert it to complete MOMP or enhancing downstream caspase activity may improve the cytotoxic effects of anti-cancer regimens. Conversely, inhibition of caspase activity downstream of minority MOMP could mitigate undesirable cytotoxicity and thereby reduce the systemic effects of chemotherapy.

## Experimental Procedures

### Plasmids

CytoGFP plasmid was cloned as follows: the cDNA encoding 1XFKBP was obtained by PCR and inserted into the pEGFP-C1 plasmid via EcoRI and BamHI restriction sites. The MitoCherry plasmid was obtained by sub-cloning the N terminus sequence of AIF (N-terminal 1–90 amino acids) into pcDNA3 via HindIII and KpnI restriction sites, followed by mCherry fused with a glycine linker in between KpnI and NotI, the FRB domain was inserted via NotI/XhoI. Both CytoGFP and MitoCherry were transiently expressed and all treatments and confocal imaging were done 24 hr later. For generating Dox-inducible, stable expression in MelJuSo cells, C-terminally HA-tagged tBID-C in pLVX-Tight-Puro (Clontech) was used as previously described (Rooswinkel et al., 2014).

### Microscopy

Both live-cell imaging and immunostaining analysis were carried out using a Nikon A1R confocal microscope (Nikon Instruments) with laser wavelengths of 405 nm, 488 nm, 561 nm, and 636 nm. Where live-cell imaging was carried out, the temperature was maintained at 37°C and CO_2_ at 5% v/v in a humidified atmosphere. Images were acquired with a 60× NA 1.4 objective.

### Micronuclei Assay

Cells were plated onto coverslips and treated for 36 hr with cytochalasin B in the culture media at a final concentration of 3 μg/ml. When the majority of cells were bi-nucleated, they were fixed in paraformaldehyde 4% in PBS for 10 min at room temperature, washed once in PBS, and mounted with DAPI-containing Vectashield. A minimum of 200 bi-nucleated cells was counted per condition.

### Statistical Analysis

For comparison of multiple groups, two-way ANOVA was used while Student’s t test was applied when comparing two groups. Analyses were performed using Prism 5.0 software (GraphPad).

## Author Contributions

G.I., J.L., and S.W.G.T. conceived and designed the study. G.I. and J.L. performed the majority of experimental work. N.M. and D.J.M. carried out in vivo BH3-mimetic/immunohistochemistry analysis and bred mice for MEF generation. E.G. generated and characterized APAF-1 deficient cells. M.H. and J.S.R. performed biotin-VAD experiments and analysis of CRISPR/Cas-9-generated CAD-deficient cells. M.J.P. and L.B.-H. provided reagents and support for the inducible caspase-9 experiments. S.U.A. and A.J.C. provided technical advice/expertise for analyzing the DNA damage response. S.M.M., D.A., and K.B. carried out and analyzed the in vivo tumorigenesis experiments. B.v.d.K. and R.W.R. developed and characterized the inducible tBID cell line. M.E.D. and M.R. carried out computational modeling experiments. A.O. provided intellectual input and, together with G.I., J.L., and S.W.G.T., wrote the manuscript.

## Supplemental Information

Document S1. Supplemental Experimental Procedures and Figures S1–S7

Movie S1. U2OS Cells Undergoing Apoptosis Showing Re-Localization of CytoGFP (Marking MOMP) prior to Apoptotic Execution, Related to Figure 1 U2OS cells transiently expressing CytoGFP and MitoCherry were treated with Act D (10 μM) and heterodimerizer and imaged every 10 min.

Movie S2. U2OS Cells Undergoing Apoptosis Showing Smac mCherry Mitochondrial Release prior to CytoGFP Mitochondrial Re-Localization, Related to Figure 1 U2OS cells transiently expressing Smac-mCherry together with CytoGFP and MitoFRB (MitoCherry lacking mCherry) were treated with Act D (10 μM) and heterodimerizer in the presence of Q-VD-OPh and imaged every 10 min.

Document S2. Article plus Supplemental Information
