# Supplementary material for: Limited Mitochondrial Permeabilization Causes DNA Damage and Genomic Instability in the Absence of Cell Death
Source: Mol Cell. 2015 Mar 5;57(5):860–72. doi: 10.1016/j.molcel.2015.01.018 (PMC4352766; doi:10.1016/j.molcel.2015.01.018)
Supplement: Document S1. Supplemental Experimental Procedures and Figures S1–S7 [file mmc1.pdf]

Molecular Cell

Supplemental Information

Limited Mitochondrial Permeabilization Causes DNA Damage and Genomic Instability in the Absence of Cell Death

Gabriel Ichim, Jonathan Lopez, Shafiq U. Ahmed, Nathiya Muthalagu, Evangelos Giampazolias, M. Eugenia Delgado, Martina Haller, Joel S. Riley, Susan M. Mason, Dimitris Athineos, Melissa J. Parsons, Bert van de Kooij, Lisa Bouchier-Hayes, Anthony J. Chalmers, Rogier W. Rooswinkel, Andrew Oberst, Karen Blyth, Markus Rehm, Daniel J. Murphy, and Stephen W.G. Tait

Figure S1

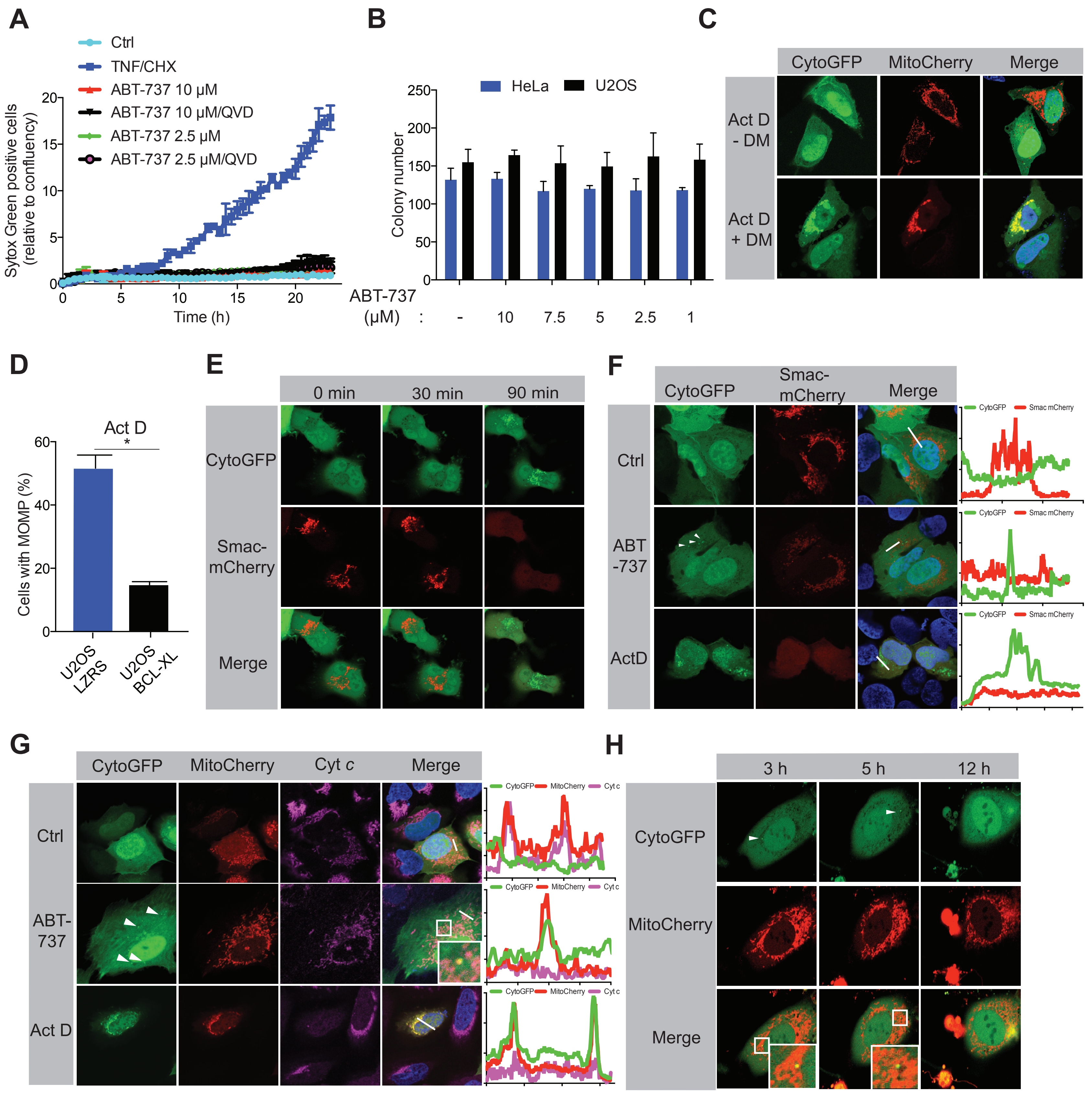
A
Ctrl
TNF/CHX
ABT-737 10 μM
ABT-737 10 μM/QVD
ABT-737 2.5 μM
ABT-737 2.5 μM/QVD
Sytox Green positive cells (relative to confluency)
Time (h)
B
HeLa
U2OS
Colony number
ABT-737 (μM) :
-
10
7.5
5
2.5
1
C
CytoGFP
MitoCherry
Merge
Act D - DM
Act D + DM
D
Act D
Cells with MOMP (%)
U2OS LZRS
U2OS BCL-XL
E
0 min
30 min
90 min
CytoGFP
Smac-mCherry
Merge
F
CytoGFP
Smac-mCherry
Merge
Ctrl
ABT -737
ActD
G
CytoGFP
MitoCherry
Cyt c
Merge
Ctrl
ABT-737
Act D
H
3 h
5 h
12 h
CytoGFP
MitoCherry
Merge

Figure S1 (related to Figure 1)

A. HeLa cells were treated with ABT-737 at the indicated concentrations over a 24-hour period and cell viability was continuously measured by Sytox Green exclusion in an Incucyte Imager. Graphs report the mean of a representative experiment repeated three times independently. As a positive control to induce apoptosis, tumour necrosis factor (TNF, 20ng/ml) cycloheximide (CHX, 1µg/ml) treatment was used. **B.** HeLa and U2OS cells were treated for 3 hours with varying concentrations of ABT-737 and quantified for long-term clonogenic survival. Graph represents the mean ± SEM of three independent experiments. **C**. U2OS cells expressing CytoGFP/MitoCherry were treated with Act D (1µM) for 3 hours in the presence or absence of heterodimeriser (DM) and imaged by confocal microscopy. **D**. U2OS cells stably expressing BCL-xL (U2OS BCL-xL) or empty vector (U2OS LZRS) together with CytoGFP/MitoCherry were treated with Act D (1µM for 12 hours) in the presence of heterodimeriser and the percentage of cells with complete MOMP was quantified. Data represent the mean ± SEM of three independent experiments. **E.** U2OS cells transiently expressing Smac-mCherry together with CytoGFP and MitoFRB (MitoCherry lacking mCherry) were treated with Act D (1µM) together with heterodimeriser and imaged by confocal microscopy for 90 minutes every 5 minutes. Representative images of the same two cells are shown over time. **F**. U2OS cells transiently expressing Smac-mCherry together with CytoGFP and MitoFRB in the presence of dimeriser were treated with ABT-737 for 3 hours. Act D was used as positive control to induce complete mitochondrial release of Smac-mCherry. Line scans represent variation in red and green fluorescence intensity along the denoted line. **G**. Same as in F, except U2OS cells transiently expressing CytoGFP and MitoCherry were immunostained for cytochrome *c*. **H.** U2OS cells transiently expressing CytoGFP and MitoCherry were treated for 3 hours prior to imaging with ABT-737 (5µM) in the presence of chemical dimeriser. Representative images over the time course are shown. Arrows and inset indicate mitochondria undergoing permeabilisation. Where stated,* represents $p<0.05$, compared versus control.

Figure S2

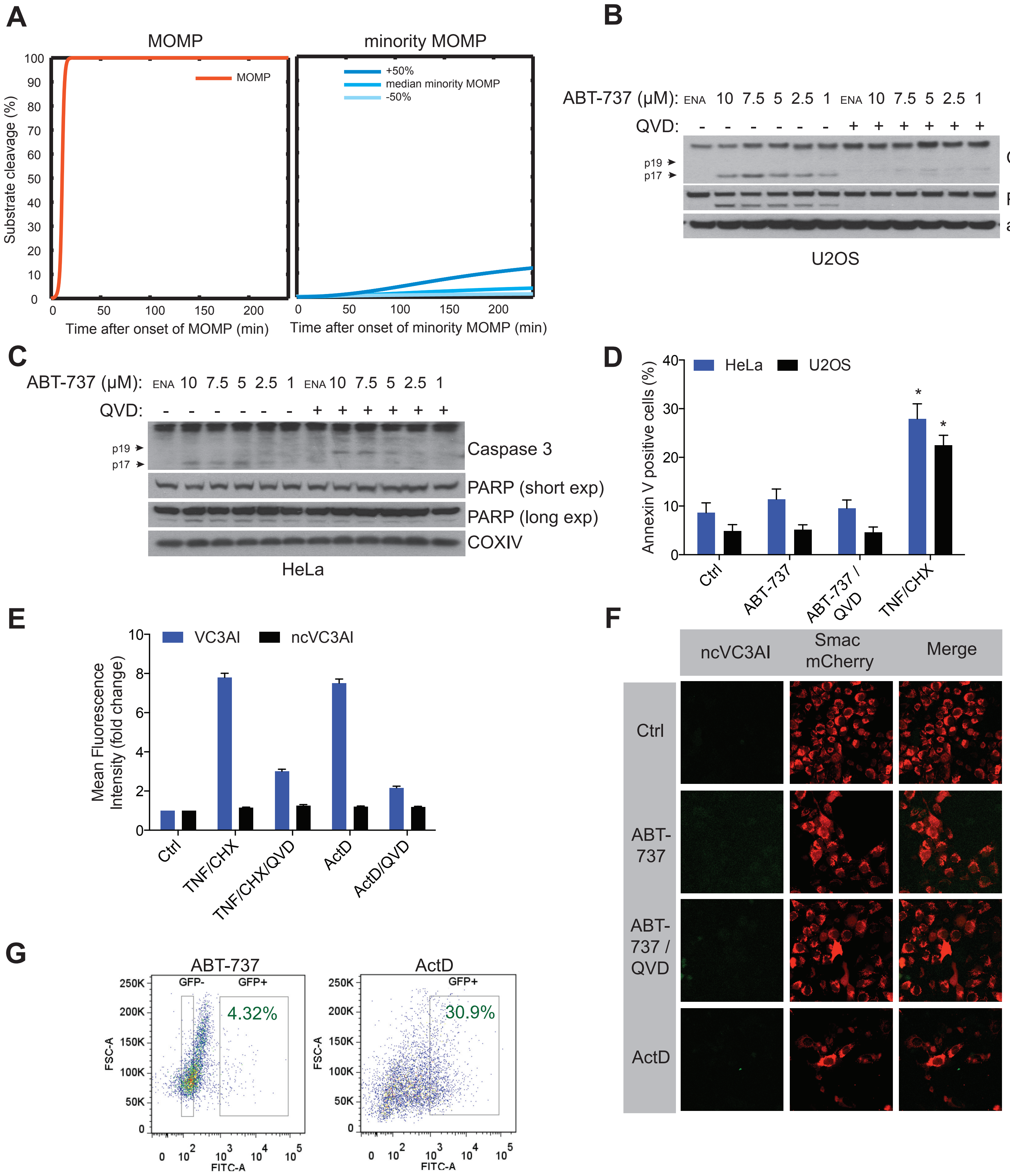

A
MOMP
minority MOMP
MOMP
+50%
median minority MOMP
-50%
Substrate cleavage (%)
Time after onset of MOMP (min)
Time after onset of minority MOMP (min)
B
ABT-737 (µM): ENA 10 7.5 5 2.5 1 ENA 10 7.5 5 2.5 1
QVD: - - - - - - + + + + + +
p19
p17
Caspase 3
PARP
actin
U2OS
C
ABT-737 (µM): ENA 10 7.5 5 2.5 1 ENA 10 7.5 5 2.5 1
QVD: - - - - - - + + + + + +
p19
p17
Caspase 3
PARP (short exp)
PARP (long exp)
COXIV
HeLa
D
HeLa
U2OS
Annexin V positive cells (%)
Ctrl
ABT-737
ABT-737 / QVD
TNF/CHX
E
VC3AI
ncVC3AI
Mean Fluorescence Intensity (fold change)
Ctrl
TNF/CHX
TNF/CHX/QVD
ActD
ActD/QVD
F
ncVC3AI
Smac mCherry
Merge
Ctrl
ABT-737
ABT-737 / QVD
ActD
G
ABT-737
ActD
GFP-
GFP+
4.32%
30.9%
FSC-A
FITC-A

Figure S2 (related to Figure 2)

A. The mathematical HeLa cell model of apoptosis execution was used to calculate the amounts of substrate cleavage by activated caspase-3 for scenarios of MOMP and minority MOMP. Model predictions demonstrate that minority MOMP triggers limited amounts of substrate cleavage, consistent with experimental validation data (see Figure 2D in the main manuscript). **B** and **C**. U2OS (B) or HeLa cells (C) were treated for 3 hours with varying doses of ABT-737 in the presence or absence of Q-VD-OPh (10μM) or enantiomer (10μM, ENA) and cell lysates were probed by Western blot for caspase-3 and PARP. **D**. HeLa and U2OS cells were treated for 3 hours with ABT-737 or TNF/CHX, stained for Annexin V and the percentage of dead cells was determined by flow-cytometry. Data represent mean ± standard error of the mean (SEM) of three independent experiments **E**. HeLa cells stably expressing the fluorescence caspase reporter VC3AI or the non-cleavable variant (ncVC3AI) were treated for 12 hours with TNF/CHX and Act D in the presence or absence of Q-VD-OPh and analysed by flow-cytometry for GFP mean fluorescence intensity. Data represent mean ± SEM of three independent experiments. **F**. HeLa cells stably expressing the non-cleavable caspase reporter (ncVC3AI) and transiently transfected with Smac-mCherry were treated with ABT-737 (10μM for 24h) and imaged by confocal microscopy; representative images are shown. **G**. Flow-cytometry dot plot profiles showing the GFP negative (ABT-737) and positive (ABT-737/Act D) cell populations sorted and assessed for clonogenic survival.

Figure S3

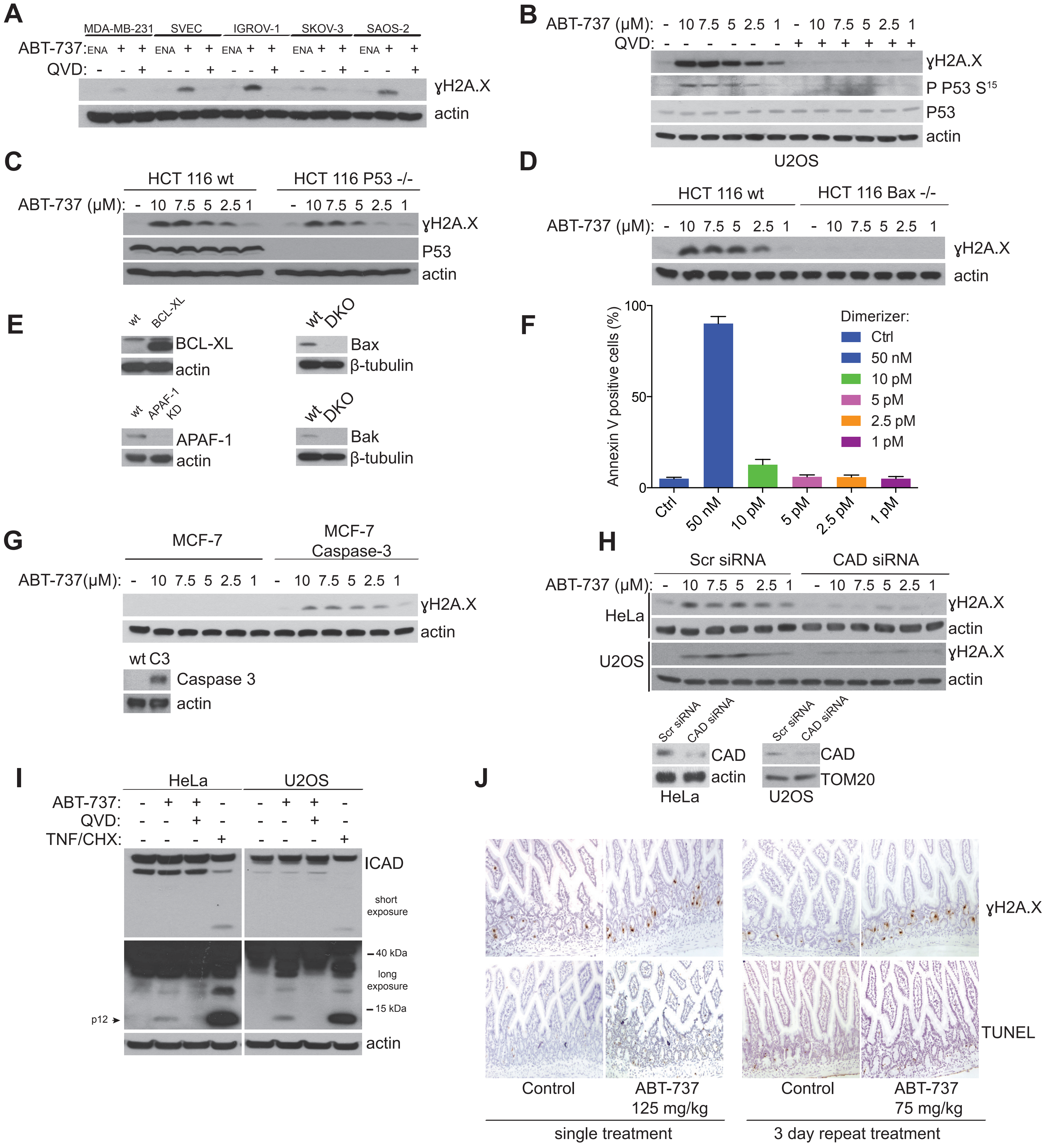
A
MDA-MB-231 SVEC IGROV-1 SKOV-3 SAOS-2
ABT-737: ENA + + ENA + + ENA + + ENA + + ENA + +
QVD: - - + - - + - - + - - + - - +
ɣH2A.X
actin
B
ABT-737 (μM): - 10 7.5 5 2.5 1 - 10 7.5 5 2.5 1
QVD: - - - - - - + + + + + +
ɣH2A.X
P P53 S15
P53
actin
U2OS
C
HCT 116 wt
HCT 116 P53 -/-
ABT-737 (μM): - 10 7.5 5 2.5 1 - 10 7.5 5 2.5 1
ɣH2A.X
P53
actin
D
HCT 116 wt
HCT 116 Bax -/-
ABT-737 (μM): - 10 7.5 5 2.5 1 - 10 7.5 5 2.5 1
ɣH2A.X
actin
E
wt BCL-XL
BCL-XL
actin
wt DKO
Bax
β-tubulin
wt APAF-1 KD
APAF-1
actin
wt DKO
Bak
β-tubulin
F
Annexin V positive cells (%)
100
50
0
Dimerizer:
Ctrl
50 nM
10 pM
5 pM
2.5 pM
1 pM
Ctrl 50 nM 10 pM 5 pM 2.5 pM 1 pM
G
MCF-7
MCF-7 Caspase-3
ABT-737(μM): - 10 7.5 5 2.5 1 - 10 7.5 5 2.5 1
ɣH2A.X
actin
wt C3
Caspase 3
actin
H
Scr siRNA
CAD siRNA
ABT-737 (μM): - 10 7.5 5 2.5 1 - 10 7.5 5 2.5 1
HeLa
ɣH2A.X
actin
U2OS
ɣH2A.X
actin
Scr siRNA CAD siRNA
CAD
actin
HeLa
Scr siRNA CAD siRNA
CAD
TOM20
U2OS
I
HeLa
U2OS
ABT-737: - + + - - + + -
QVD: - - + - - - + -
TNF/CHX: - - - + - - - +
ICAD
short exposure
40 kDa
long exposure
15 kDa
p12
actin
J
ɣH2A.X
TUNEL
Control
ABT-737 125 mg/kg
Control
ABT-737 75 mg/kg
single treatment
3 day repeat treatment

Figure S3 (related to Figure 3)

A. The indicated cell lines were treated for 3 hours with ABT-737 (1μM) or enantiomer (1μM, ENA) and immunoblotted for γH2A.X. **B**. U2OS cells were treated with ABT-737 as in (A) and immunoblotted for phospho-S^{15} p53 and total p53. **C**. Wild type HCT-116 or HCT-116 p53 -/- cells were treated with ABT-737 as in (B) and cell lysates were probed for γH2A.X. **D**. Wild type HCT-116 and HCT-116 BAX -/- were treated and analysed as in (C). **E**. Expression analysis, Western blot for HeLa BCL-xL and HeLa APAF-1 KD, respectively. Right panel, Western blot validation of BAX and BAK knock-out MEF. **F**. A549 cells inducible for Caspase-9 dimerization/activation were treated for 3 hours with the indicated concentrations of homodimerizer (DM), stained for Annexin V and analysed by flow cytometry for cell death. Data represent mean ± standard error of the mean (SEM) of three independent experiments. **G**. Wild type MCF-7 or MCF-7 stably expressing Caspase-3 were treated with ABT-737 as in (C) and cell lysates were probed for γH2A.X. **H**. HeLa and U2OS cells were transfected with CAD siRNA and then treated with ABT-737 as in (B). The lower panels show the efficacy of CAD knock-down determined by Western blot. **I**. U2OS and HeLa cells were treated with ABT-737 (10μM for 3 hours) and cell lysates were blotted for ICAD (antibody recognizing both full length and cleaved ICAD). TNF/CHX treatment was used as positive control for ICAD cleavage. **J** Representative images of γH2A.X and TUNEL staining in small intestine from mice treated with ABT 737 (125 mg/kg for 1 day or 75 mg/kg for three consecutive days). Control mice were administered with vehicle.

Figure S4

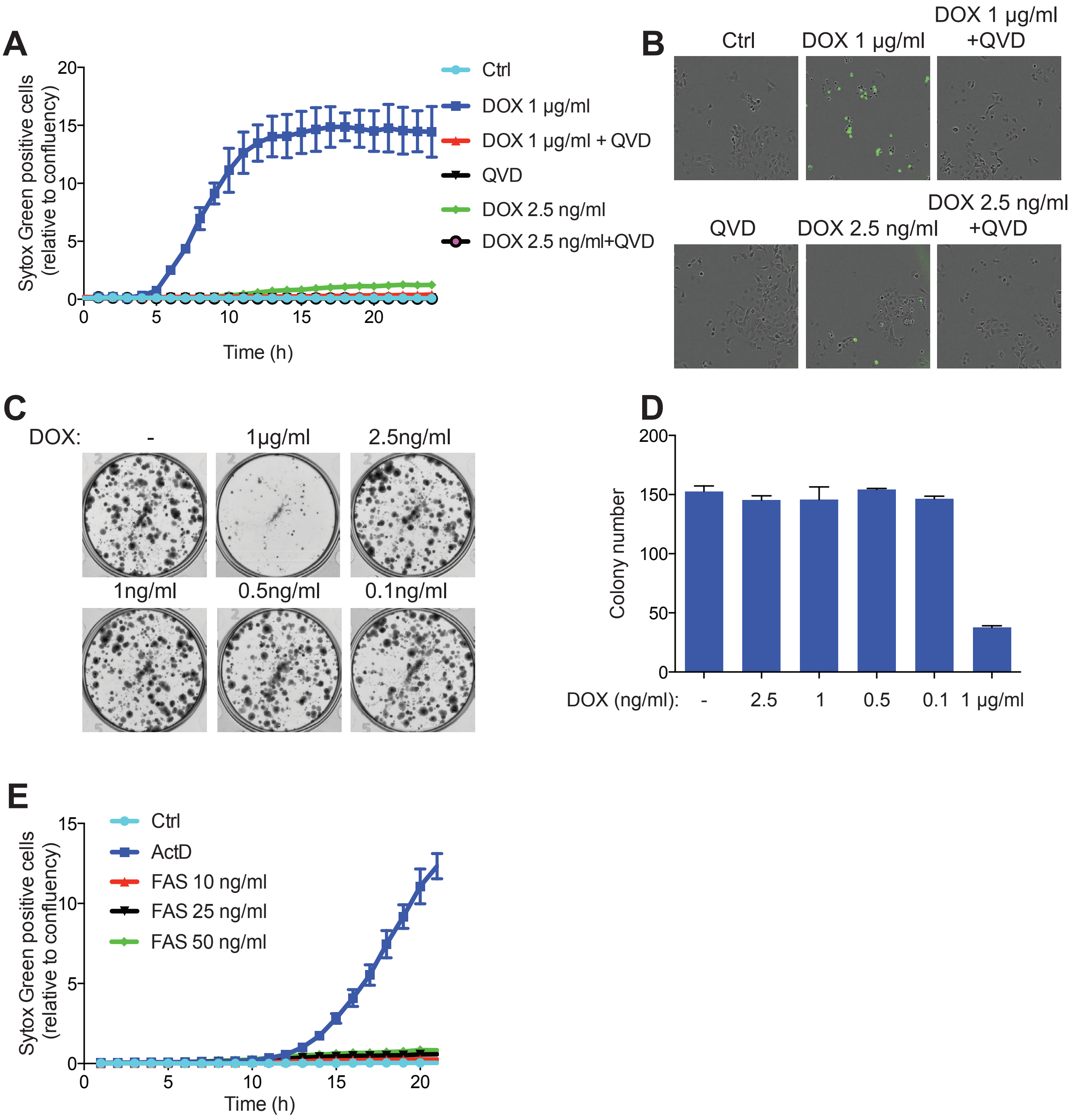

A
Sytox Green positive cells (relative to confluency)
Time (h)
Ctrl
DOX 1 µg/ml
DOX 1 µg/ml + QVD
QVD
DOX 2.5 ng/ml
DOX 2.5 ng/ml+QVD
B
Ctrl
DOX 1 µg/ml
DOX 1 µg/ml +QVD
QVD
DOX 2.5 ng/ml
DOX 2.5 ng/ml +QVD
C
DOX:
-
1µg/ml
2.5ng/ml
1ng/ml
0.5ng/ml
0.1ng/ml
D
Colony number
DOX (ng/ml):
-
2.5
1
0.5
0.1
1 µg/ml
E
Sytox Green positive cells (relative to confluency)
Time (h)
Ctrl
ActD
FAS 10 ng/ml
FAS 25 ng/ml
FAS 50 ng/ml

Figure S4 (related to Figure 4)

A. MelJuSo tBid tetON cells were treated for 24 hours with the indicated concentrations of doxycycline (DOX) and cell viability was measured by Sytox Green exclusion in an Incucyte Imager. One representative experiment out of three independent repeats is shown. **B**. Images of MelJuSo tBid tetON cells treated with DOX as in (A). Images were taken at 12 hour after DOX treatment, Sytox Green positive cells are non-viable. **C** and **D**. MelJuSo tBid tetON cells were treated for 3 hours with the indicated concentrations of DOX followed by long-term clonogenic survival assay. Representative images are shown in (C) and quantitation is depicted in (D). Data represent mean ± SEM from three independent experiments. **E**. U2OS cells were treated with FAS ligand and CHX at the indicated concentrations and cell viability was measured by Sytox Green exclusion. One representative experiment is shown (repeated three times independently).

Figure S5

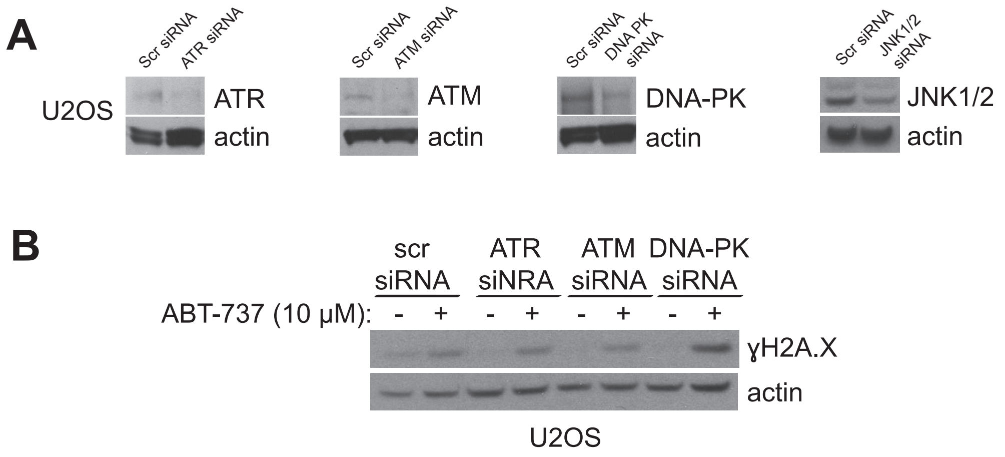
A
Scr siRNA
ATR siRNA
U2OS
ATR
actin
Scr siRNA
ATM siRNA
ATM
actin
Scr siRNA
DNA PK siRNA
DNA-PK
actin
Scr siRNA
JNK1/2 siRNA
JNK1/2
actin
B
scr siRNA
ATR siNRA
ATM siRNA
DNA-PK siRNA
ABT-737 (10 μM): - + - + - + - +
ɣH2A.X
actin
U2OS

Figure S5 (related to Figure 5)

A. Western blot analysis of ATM, ATR, DNA PK and JNK1/2 expression in U2OS cells following siRNA transfection. **B**. U2OS cells expressing siRNA targeting ATR, ATM or DNA-PK were treated with ABT-737 (10μM for 3 hours) and cell lysates were Western blotted for γH2A.X.

Figure S6

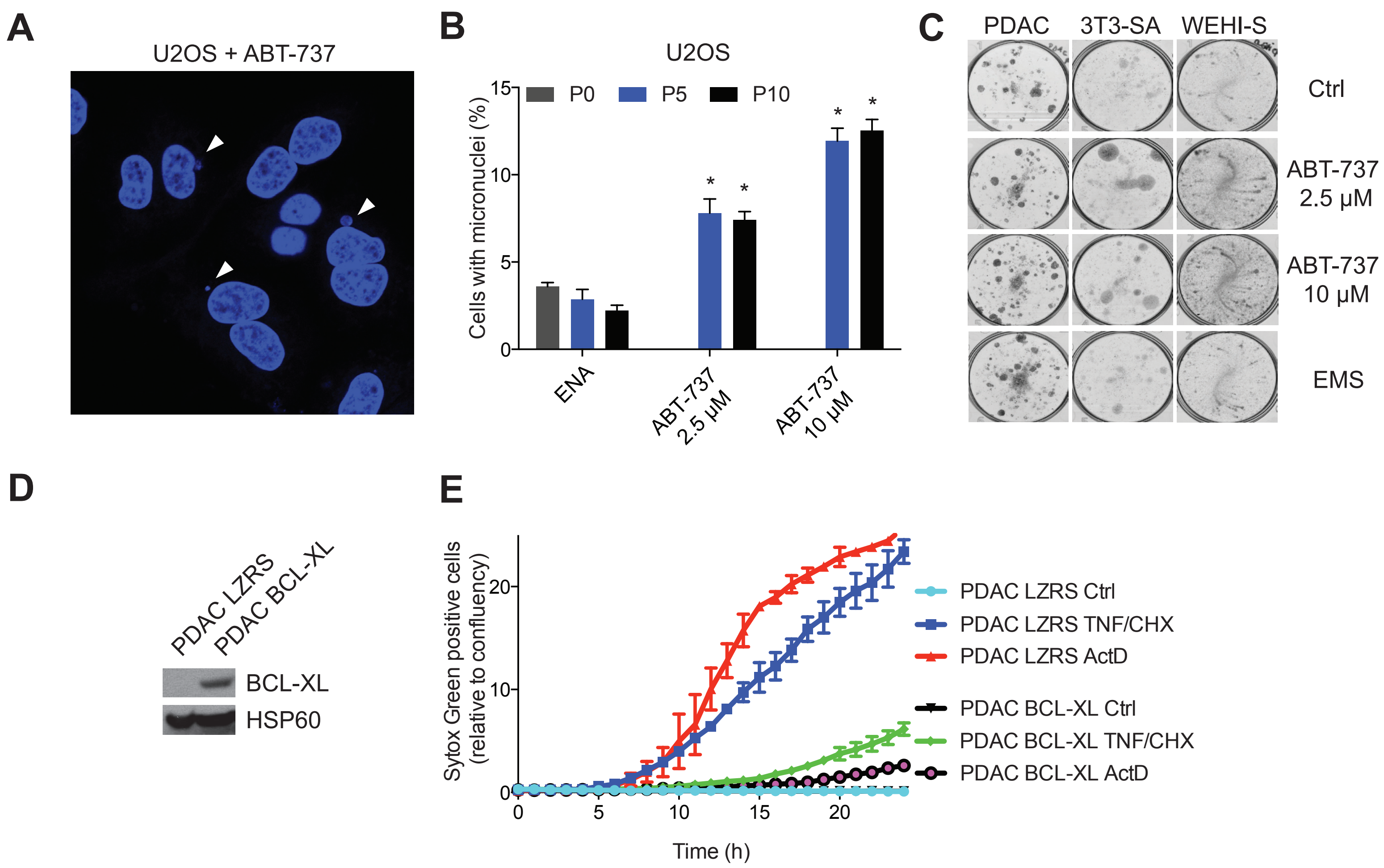
A
U2OS + ABT-737
B
U2OS
P0
P5
P10
Cells with micronuclei (%)
15
10
5
0
ENA
ABT-737 2.5 μM
ABT-737 10 μM
C
PDAC
3T3-SA
WEHI-S
Ctrl
ABT-737 2.5 μM
ABT-737 10 μM
EMS
D
PDAC LZRS
PDAC BCL-XL
BCL-XL
HSP60
E
Sytox Green positive cells (relative to confluency)
20
10
0
5
15
Time (h)
PDAC LZRS Ctrl
PDAC LZRS TNF/CHX
PDAC LZRS ActD
PDAC BCL-XL Ctrl
PDAC BCL-XL TNF/CHX
PDAC BCL-XL ActD

Figure S6 (related to Figure 6)

A. Representative image of U2OS cells treated daily for 10 passages with ABT-737 (10μM). Nuclei were stained using DAPI. **B**. U2OS cells were treated daily with ABT-737 or enantiomer (10μM, Ctrl) at the indicated concentrations for either 5 (P5) or 10 (P10) passages and then scored for micronuclei. Data represent mean ± SEM of three independent experiments. **C**. Representative images of PALA-resistant colonies for PDAC, 3T3-SA and WEHI-S. **D**. Analysis of BCL-xL expression by Western blot in stably overexpressing PDAC cells. **E**. Incucyte viability analysis of PDAC cells stably expressing BCL-xL (PDAC BCL-xL) or empty vector (PDAC LZRS) and treated for 24 hours with the indicated apoptotic stimuli.

Figure S7

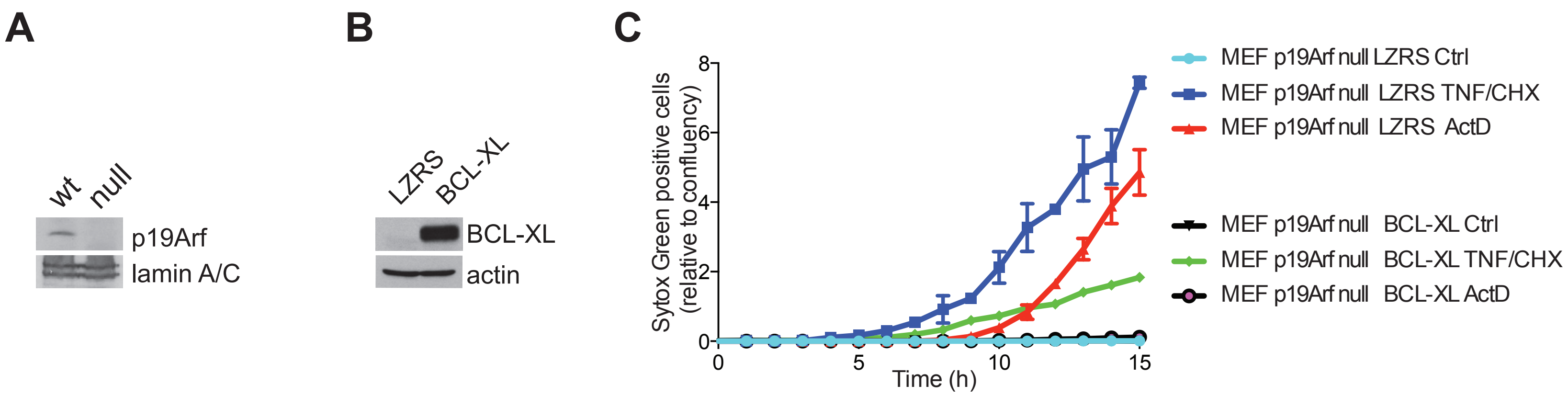
A
wt
null
p19Arf
lamin A/C
B
LZRS
BCL-XL
BCL-XL
actin
C
Sytox Green positive cells
(relative to confluency)
8
6
4
2
0
0
5
10
15
Time (h)
MEF p19Arf null LZRS Ctrl
MEF p19Arf null LZRS TNF/CHX
MEF p19Arf null LZRS ActD
MEF p19Arf null BCL-XL Ctrl
MEF p19Arf null BCL-XL TNF/CHX
MEF p19Arf null BCL-XL ActD

Figure S7 (related to Figure 7)

A. Western blot validation of $p19^{Arf}$ deficiency in $p19^{Arf}$ null MEFs. **B**. Western blot analysis of BCL-xL expression in vector and BCL-xL transduced $p19^{Arf}$ null MEF. **C**. Incucyte, Sytox Green exclusion-based viability analysis of $p19^{Arf}$ null MEF stably expressing BCL-xL and treated for 15 hours with the indicated apoptotic stimuli.

Supplemental Experimental Procedures

Cell lines and reagents

All cell lines were maintained in DMEM high glucose medium supplemented with 10% FCS, 2mM glutamine, 1mM sodium pyruvate, 50μM β-mercaptoethanol and penicillin/streptomycin. ABT-737 and Q-VD-OPh were obtained from ApexBio, the ABT-737 enantiomer was obtained from Abbvie Pharmaceuticals. PALA compound was obtained through the NCI Developmental Therapeutics Program.

Microscopy

For live cell imaging, cells were plated at 2 x 10^5 cells in glass bottom 35 mm microwell dishes (MatTek Corporation), transfected next day as indicated in the respective figure legend and imaged 24 hour later. Immunofluorescence staining for γH2A.X and cytochrome *c* was as follows: U2OS cells transiently expressing CytoGFP and MitoCherry, were fixed in 4% PFA/PBS for 10 minutes and permeabilised in 0.2 % Triton/PBS for 15 minutes followed by 1 hour blocking in 2% BSA/PBS. The primary antibody for phospho H2A.X (Cell Signalling, 2577, 1/100 in PBS) and cytochrome *c* (BD Biosciences, 556432, 1/300 in PBS) was incubated overnight. AlexaFluor 647 goat anti-rabbit was used as secondary antibody (Life Technologies, A21245, 1/300 in PBS). For visualizing minority MOMP, U2OS, HeLa, MelJuso tBID tetON or PDAC cells were transiently transfected in 12-well plates using GeneJuice. 24 hours later, cells were treated as described in the figures legend together with A/C heterodomerizer (50nM, Clontech, 635057). Cells were then fixed with PFA 4% in PBS for 10 minutes at room temperature and mounted using Vectashield mounting medium with DAPI (Vector Laboratories, H-1200). Cells displaying minority MOMP were then scored under the confocal microscope. Images were analyzed with ImageJ 1.47i.

E1A/Ras transformation and soft-agar assays

Primary MEF cells were isolated from E14 embryos (C57BL/6 strain) and cultured with ABT-737 for 5 passages. Cells were then plated in 6-well plates, in triplicate at 500, 1000, 2500 or 5000 cells/well. The following day cells were

infected with retroviruses encoding E1A and *KRas*. 24 hours later fresh media with 0.5 µg/ml puromycin (for *KRas*) and 40µg/ml hygromycin B (for E1A) was added and 7 days later the colonies were stained using methylene blue (1 % w/v in 50/50 methanol/water v/v). For soft-agar assays, primary p19^{ARF} null MEF were generated from E14 embryos derived from *Arf*-null mice *Cdkn2atm1(GFP)Cjs* (*ARF GFP*) mice, wherein GFP is inserted into exon 1 β of the *Cdkn2a* locus, abrogating p19^{ARF} expression (Zindy et al., 2003). Cells were treated with enantiomer or ABT-737 (10μM) in the presence or absence of caspase-inhibitor Q-VD-OPh for 10 passages. Alternatively, cells were transduced with empty LZRS-zeo retrovirus or retrovirus encoding BCL-xL and selected with Zeocin (200μg/ml) prior to treatment. Soft agar assay was performed as follows: a 1% base agarose solution (Low gelling temperature agarose, Sigma, A9414-25G) was poured into 6-well plates and allowed to solidify. Meanwhile, 7500 cells were suspended in 0.6% agarose solution in order to reach a final concentration of 0.3% and then laid on top of the base agarose. When the agarose solution solidified the dish was covered in complete DMEM media and colonies were counted 14 days later. Representative images for the soft agarose colonies were taken using Qcapture Pro7 software (QImaging).

Immunohistochemistry

All immunohistochemistry (IHC) staining was performed on Zinc-formalin fixed, paraffin-embedded sections (4µm thick). TUNEL staining was performed using the ApopTag peroxidase labelling kit according to the manufacturer's instructions (Millipore, S7100). An additional blocking step (1% BSA for 1hr at RT) was incorporated prior to addition of peroxidase-conjugated anti-digoxigenin. Anti-phospho H2A.X (Cell Signalling, 2577) and anti-pJNK (Thr183/Tyr185) (Abcam, ab18680) were used at 1:50 dilution after antigen retrieval with sodium citrate buffer (pH 6.0). All tissues were counterstained in Gil 1 haematoxylin followed by blueing solution.

Western blotting

Cell lysates were prepared using NP-40 lysis buffer (1% NP-40, 1mM EDTA, 150mM NaCl, 50mM Tris pH7.4, 1mM PMSF, Complete Protease Inhibitors [Roche]). Protein content was determined by Bio-Rad assay, proteins were separated by SDS-PAGE and blotted onto nitrocellulose. Membranes were probed with antibodies at 1/1000 dilution unless otherwise stated: anti-actin (MP Biomedicals, 8691001, 1/10000), anti-Tom20 (Santa Cruz, sc-11415), anti-BCL-xL (Abcam, ab32370), anti-cytochrome *c* (BD, 556433), anti-COXIV (Cell Signalling, 4850), anti-caspase-3 (Cell Signalling, 9662), anti-PARP (Cell Signalling, 9532, 1/1000), anti-γH2A.X (Cell Signalling, 9718S), anti-HSP60 (Santa Cruz, sc-13115), anti-BAK (Cell Signalling, 12105), anti-BAX (Santa Cruz, sc-493), anti-ICAD (Abcam, ab108521), anti-CAD (Santa Cruz, sc-374067), anti-APAF1 (Cell Signalling, 8723), anti-DNA-PK (Abcam, ab1832), anti-ATM (Abcam, ab2618), anti-P-ATM S1981 (Novus, 100-307), anti-ATR (Santa Cruz, sc-1887, 1/250), anti-P-ATR S428 (Cell Signalling, 2853), anti-JNK (Cell Signalling, 9252S), anti-P-JNK T183/Y185 (Cell Signalling, 4668S, 1/1000), anti-p53 (BD Biosciences, 554293), anti-P-S10 p53 (Cell Signalling, 9286), anti caspase-7 (Cell Signalling, 9492), anti caspase-9 (Cell Signalling, 9502S), anti caspase-8 (Enzo, ALX-804-429-C050) antibodies followed by incubation with the appropriate HRP conjugated secondary antibody and detection of immunoreactive proteins by ECL.

Comet assay

The alkaline Comet assay was used as described in the manufacturer's manual (Trevigen, 4250-050-K). The comet tail length was measured in ImageJ.

zVAD-biotin pull-downs

For caspase-3 and-7 pull-down, HeLa cells were treated with the indicated concentrations of ABT-737, Act D and TNF/CHX for 3 hours and then zVAD-biotin (Santa Cruz, sc-311290) was incubated with the cells for another 3 hours at 50μM final concentration. Cell were lysed in RIPA buffer (150 mM NaCl, 0.5% sodium deoxycholate, 0.1% SDS, and 50 mM Tris, pH 8.0) and

250 µg total protein (determined by Bio-Rad assay) were incubated overnight at 4°C with neutravidin agarose resin (Thermo Scientific, 29202). The following day, the resin was washed 3 times in RIPA buffer and once in PBS and then denaturated at 95°C with 30 µl sample buffer. For the caspase-9 and caspase-8 pulldowns the procedure was identical except zVAD-biotin was incubated with the cells for 1 hour prior to and maintained throughout the treatment period.

Generation of CAD deficient cells by CRISPR/Cas9 genome editing

Oligos targeting the following human CAD sequence: CAGCCCGAGGAAGTTCGGCG were cloned into LentiCRISPRv1 plasmid (obtained from Addgene, 49535) as previously described (Shalem et al., 2014). Following transduction, CAD-deficient HeLa and U20S cells were selected with puromycin for 2 weeks prior to analysis.

PALA assay

The PALA LD_{50} was determined for the three murine cell lines. Cells were cultured in nucleoside-free α-MEM containing Pen/Strep and 10% dialyzed FBS. Briefly, cells were plated in 96-well plate at a density of 500 and 1000 cells per well. PALA treatment was added at concentrations ranging from 0 to 100 µM. Three days later, MMT assay was performed to determine viability. Briefly, 10 % (v/v) MTT (5 mg/ml in PBS) was added to the media and incubated for 2 hours at 37°C. DMSO was added to solubilize the MTT crystals and the absorbance was read at 560 nm. For PALA resistance assay, cells were cultured in triplicates in 6-well plates at a density of 2500 cells/well. PALA was added at $3xLD_{50}$ and the treatment was carried out until colonies were visible. At the end of the experiment, colonies were stained using methylene blue (1 % in 50/50 methanol/water v/v). qPCR was performed on PALA-resistant colonies to assess *Cad* genomic amplification as previously described (Mathew et al., 2009). PCR fragments were amplified 3 min at 95°C, followed by 40 cycles of 20 sec at 95°C, 30 sec at 57°C, 30 sec at 72°C and final 5min at 72°C. The following primers were used: mCAD For AAGCTCAGATCCTAGTGCTAACG, mCAD Rev –

CCGTAGTTGCCGATGAGAGG, m18S For – ATGGTAGTCGCCGTGCCTAC and m18S Rev – CCGGAATCGAACCCTGATT.

Digitonin fractionation

Cells were washed once in PBS and re-suspended in a solution containing 70 mM Tris and 250 mM sucrose, pH 7.0. Digitonin (Sigma-Aldrich, D5628) was added to 100 µg/ml on ice for 1 minute and the cells were immediately centrifuged at 3000 rpm for 3 minutes. The supernatant was collected as cytosol and the pellet was solubilised in RIPA buffer (150 mM NaCl, 0.5% sodium deoxycholate, 0.1% SDS, and 50 mM Tris, pH 8.0) to obtain the whole cell lysate fraction.

Retroviral and lentiviral transduction

Phoenix Ampho (for human retrovirus), Phoenix Eco (for mouse retrovirus) or 293T cells (for lentivirus) (2×10^6 in a 10 cm dish) were transfected with LZRS zeo, LZRS FLAG-BCL-xL, pCDH-puro-CMV-VC3AI, pCDH-puro-CMV-ncVC3AI, pWZLH.E1A or pBabePuro.H-ras using Lipofectamine 2000 (Invitrogen) according to the manufacturer's instructions. The helper plasmids for lentivirus production were pVSVg (Addgene 8454)(Stewart et al., 2003) and psPAX2 (Addgene 12260). Two days later virus-containing supernatant was harvested, filtered and used infect target cells in the presence of 1μg/ml polybrene. Two days post-infection, stably expressing cells were selected by growth in 200ug/ml Zeocin (Invitrogen) or 1ug/ml puromycin, respectively. MelJuSo cells with Dox-inducible expression of tBid proteins were created by sequential lentiviral transduction with pLVX-Tet-On Advanced (Clontech) and pLVX-tight encoding tBid as previously described (Rooswinkel et al., 2014).

siRNA and plasmid transfection

Cells were transfected with 25 nM non-targeting control or the individual RNAi duplexes (Thermo Scientific) using Lipofectamine 2000 (Invitrogen) according to the manufacturer’s protocol. Alternatively, for plasmid transfection GeneJuice transfectant (Merck Millipore) was used according to the manufacturer's instructions. The following siRNA oligos were used: hCAD

(Dharmacon, D-004425-01), hJNK1 (Dharmacon, M-003514-04), hJNK2 (Dharmacon, M-003505-02), hATM (Dharmacon, M-003201-04), hATR (Dharmacon, M-003202-05), hDNA-PK (Dharmacon, M-005030-01).

Cell viability assays

Cell viability was determined using an Incucyte FLR imaging system (Essen Bioscience). Cells were plated in medium containing 30nM Sytox Green (Invitrogen S7020). Cells were treated as described, imaged every 60 minutes over a period of 24 hours and analysed using Incucyte image analysis software (Essen Bioscience). The number of SytoxGreen positive cells was normalized to the confluency factor of the respective well. Alternatively, cell viability was analysed by flow cytometry using a FACS calibur (BD Biosciences). For this purpose, cells were stained with Alexa Fluor 647-Annexin V (BioLegend) according to the manufacturer's protocols. Analysis was performed using CellQuest Pro software (BD Biosciences).

Caspase-reporter based cell sorting

HeLa cells stably expressing the VC3AI caspase-3 reporter were treated for 24 hours with ABT-737 (10 μM) or 12 hours with 0.5 μM actinomycin D. Following treatment, equal numbers of GFP positive (ABT-737 or actinomycin D treated) or negative (ABT-737 treated sample) were sorted into 6-well plates using a FACSAria Fusion cell sorter (BD Biosciences). Clonogenic survival was quantified after 2 weeks using methylene blue (1 % w/v in 50/50 methanol/water v/v).

Mathematical modelling

The mathematical model of apoptosis execution has been described previously (Rehm et al., 2006). In brief, it comprises 19 reaction partners and 53 reactions, implemented as ordinary differential equations on the basis of mass action kinetics. The model has been parameterised and validated to accurately simulate the non-linear signalling from MOMP to caspase-3 activation and apoptosis execution in HeLa cervical cancer cells. For the analysis of the consequences of minority MOMP, here the model was adapted by modulating the strength of model input functions for mitochondrial Smac

release and cytochrome *c* driven caspase-9 activation, according to quantitative measurements of minority MOMP (Figure 2A). Calculations of the total amount of processed caspase-3 and the resulting cleavage of caspase-3 substrates served as model outputs. Simulations were conducted using MatLab (The Mathworks, UK).

In vivo experiments

All procedures were approved by Animal Welfare and Ethical Review Board (University of Glasgow) and carried out in accordance with the Animals (Scientific Procedures) Act 1986 and guidelines of the Committee of the National Cancer Research Institute. C57BL/6 mice were treated with ABT-737 (single intraperitoneal injection, 75 mg/kg and 125 mg/kg of ABT-737 or 3 consecutive days treatment of 75 mg/kg single dose per day), while control mice were treated with vehicle (30% v/v propylene glycol, 5% v/v Tween, 5% w/v dextrose, pH4). For a positive TUNEL staining control, mice were treated with doxorubicin (single intraperitoneal injection, 10 mg/kg). For tumourigenesis experiments, 7-week old CD1-*Nude* female mice were injected subcutaneously with 5 x 10^6 cells (15 mice per group). Tumor formation was monitored and tumor volume based on caliper measurements was calculated by the formula: tumor volume = (length x width x width)/2.

Supplemental References

Mathew, R., Karantza-Wadsworth, V., and White, E. (2009). Assessing metabolic stress and autophagy status in epithelial tumors. Methods in enzymology *453*, 53-81.

Rehm, M., Huber, H.J., Dussmann, H., and Prehn, J.H. (2006). Systems analysis of effector caspase activation and its control by X-linked inhibitor of apoptosis protein. The EMBO journal *25*, 4338-4349.

Rooswinkel, R.W., van de Kooij, B., de Vries, E., Paauwe, M., Braster, R., Verheij, M., and Borst, J. (2014). Antiapoptotic potency of Bcl-2 proteins primarily relies on their stability, not binding selectivity. Blood *123*, 2806-2815.

Shalem, O., Sanjana, N.E., Hartenian, E., Shi, X., Scott, D.A., Mikkelsen, T.S., Heckl, D., Ebert, B.L., Root, D.E., Doench, J.G., *et al.* (2014). Genome-scale CRISPR-Cas9 knockout screening in human cells. Science *343*, 84-87.

Stewart, S.A., Dykxhoorn, D.M., Palliser, D., Mizuno, H., Yu, E.Y., An, D.S., Sabatini, D.M., Chen, I.S., Hahn, W.C., Sharp, P.A., *et al.* (2003). Lentivirus-delivered stable gene silencing by RNAi in primary cells. Rna *9*, 493-501.

Zindy, F., Williams, R.T., Baudino, T.A., Rehg, J.E., Skapek, S.X., Cleveland, J.L., Roussel, M.F., and Sherr, C.J. (2003). Arf tumor suppressor promoter monitors latent oncogenic signals in vivo. Proceedings of the National Academy of Sciences of the United States of America *100*, 15930-15935.
