# Supplementary figures and images for: Limited Mitochondrial Permeabilization Causes DNA Damage and Genomic Instability in the Absence of Cell Death
Source: Mol Cell. 2015 Mar 5;57(5):860–72. doi: 10.1016/j.molcel.2015.01.018 (PMC4352766; doi:10.1016/j.molcel.2015.01.018)

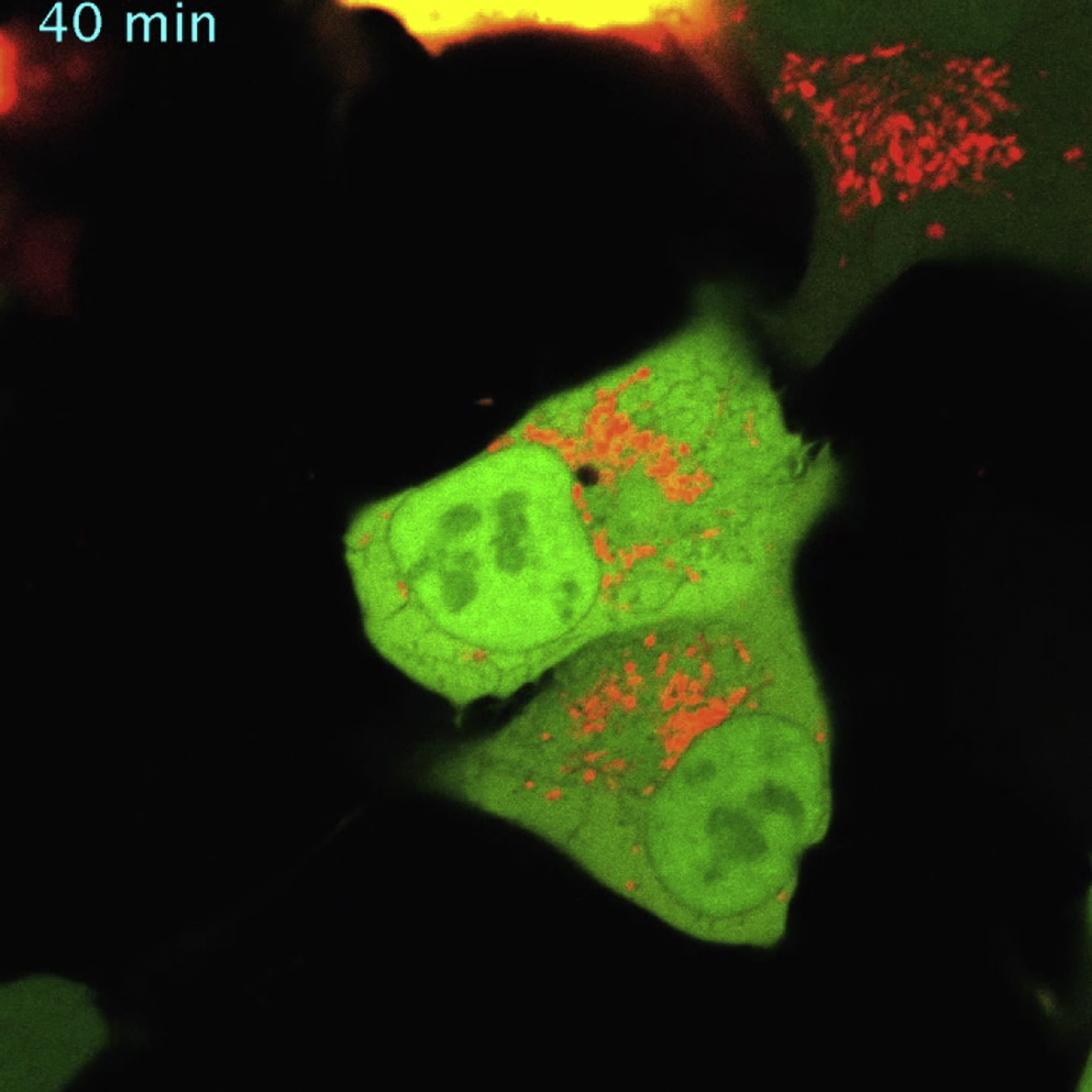

Supplement: Movie S1. U2OS Cells Undergoing Apoptosis Showing Re-Localization of CytoGFP (Marking MOMP) prior to Apoptotic Execution, Related to Figure 1 — U2OS cells transiently expressing CytoGFP and MitoCherry were treated with Act D (10 μM) and heterodimerizer and imaged every 10 min. [file mmc2.jpg]

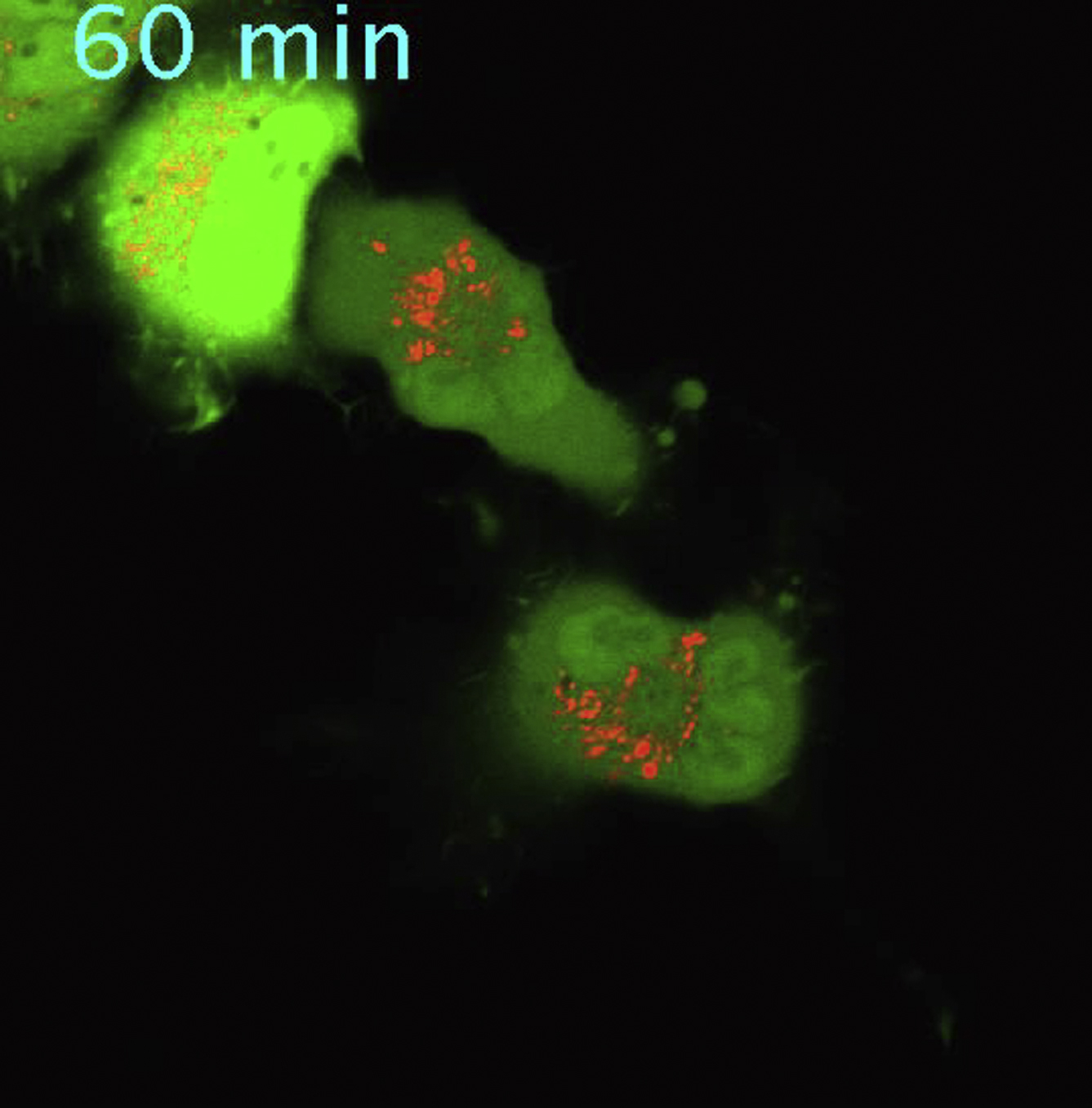

Supplement: Movie S2. U2OS Cells Undergoing Apoptosis Showing Smac mCherry Mitochondrial Release prior to CytoGFP Mitochondrial Re-Localization, Related to Figure 1 — U2OS cells transiently expressing Smac-mCherry together with CytoGFP and MitoFRB (MitoCherry lacking mCherry) were treated with Act D (10 μM) and heterodimerizer in the presence of Q-VD-OPh and imaged every 10 min. [file mmc3.jpg]
