# Supplementary material for: Limited Mitochondrial Permeabilization Causes DNA Damage and Genomic Instability in the Absence of Cell Death
Source: Mol Cell. 2015 Mar 5;57(5):860–72. doi: 10.1016/j.molcel.2015.01.018 (PMC4352766; doi:10.1016/j.molcel.2015.01.018)
Supplement: Document S2. Article plus Supplemental Information [file mmc4.pdf]

Molecular Cell

Article

Limited Mitochondrial Permeabilization Causes DNA Damage and Genomic Instability in the Absence of Cell Death

Graphical Abstract

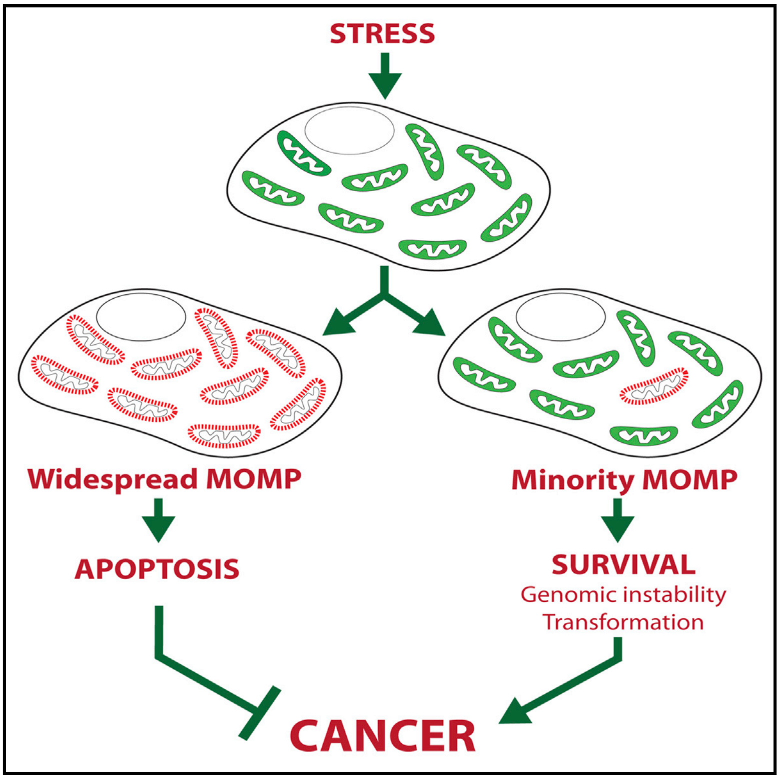

Highlights

- MOMP can occur in a minority of mitochondria
- Minority MOMP triggers caspase activity but fails to kill cells
- Minority MOMP-induced caspase activity causes DNA damage and genomic instability
- Minority MOMP promotes cellular transformation and tumorigenesis

Authors

Gabriel Ichim, Jonathan Lopez, ..., Daniel J. Murphy, Stephen W.G. Tait

Correspondence

stephen.tait@glasgow.ac.uk

In Brief

During apoptosis, mitochondrial outer membrane permeabilization (MOMP) is widespread, leading to rapid cell death. Here, Ichim et al. demonstrate that MOMP can also be engaged in a minority of mitochondria without killing the cell. Instead, minority MOMP triggers caspase-dependent DNA damage and genomic instability, thereby promoting transformation and tumorigenesis.

Ichim et al., 2015, Molecular Cell *57*, 860–872
March 5, 2015 ©2015 The Authors
http://dx.doi.org/10.1016/j.molcel.2015.01.018

Limited Mitochondrial Permeabilization Causes DNA Damage and Genomic Instability in the Absence of Cell Death

Gabriel Ichim,[1,2] Jonathan Lopez,[1,2] Shafiq U. Ahmed,[2] Nathiya Muthalagu,[1,2] Evangelos Giampazolias,[1,2] M. Eugenia Delgado,[3] Martina Haller,[1,2] Joel S. Riley,[1,2] Susan M. Mason,[1] Dimitris Athineos,[1] Melissa J. Parsons,[4,5] Bert van de Kooij,[6] Lisa Bouchier-Hayes,[4,5] Anthony J. Chalmers,[2] Rogier W. Rooswinkel,[6] Andrew Oberst,[7] Karen Blyth,[1] Markus Rehm,[3] Daniel J. Murphy,[1,2] and Stephen W.G. Tait[1,2,*]
[1]Cancer Research UK Beatson Institute
[2]Institute of Cancer Sciences, University of Glasgow
Garscube Estate, Switchback Road, Glasgow G61 1BD, UK
[3]Centre for Systems Medicine, Royal College of Surgeons in Ireland, Dublin 2, Ireland
[4]Center for Cell and Gene Therapy
[5]Department of Pediatrics-Hematology
Baylor College of Medicine, Houston, TX 77030, USA
[6]Division of Immunology, The Netherlands Cancer Institute, Plesmanlaan 121, Amsterdam 1066 CX, the Netherlands
[7]Department of Immunology, University of Washington, 750 Republican Street, Seattle, WA 98109, USA
*Correspondence: stephen.tait@glasgow.ac.uk
http://dx.doi.org/10.1016/j.molcel.2015.01.018
This is an open access article under the CC BY license (http://creativecommons.org/licenses/by/4.0/).

SUMMARY

During apoptosis, the mitochondrial outer membrane is permeabilized, leading to the release of cytochrome *c* that activates downstream caspases. Mitochondrial outer membrane permeabilization (MOMP) has historically been thought to occur synchronously and completely throughout a cell, leading to rapid caspase activation and apoptosis. Using a new imaging approach, we demonstrate that MOMP is not an all-or-nothing event. Rather, we find that a minority of mitochondria can undergo MOMP in a stress-regulated manner, a phenomenon we term "minority MOMP." Crucially, minority MOMP leads to limited caspase activation, which is insufficient to trigger cell death. Instead, this caspase activity leads to DNA damage that, in turn, promotes genomic instability, cellular transformation, and tumorigenesis. Our data demonstrate that, in contrast to its well-established tumor suppressor function, apoptosis also has oncogenic potential that is regulated by the extent of MOMP. These findings have important implications for oncogenesis following either physiological or therapeutic engagement of apoptosis.

INTRODUCTION

Following most apoptotic stimuli, the pro-apoptotic BCL-2 family members Bax and Bak permeabilize the outer membrane of the mitochondria, an event termed "mitochondrial outer membrane permeabilization" (MOMP). MOMP leads to rapid cell death by releasing mitochondrial proteins including cytochrome *c* that activate caspases (Tait and Green, 2010). However, even in the absence of caspase activity, cells typically die once MOMP has occurred, most likely due to progressive mitochondrial dysfunction (Lartigue et al., 2009; Tait et al., 2014). Due to these catastrophic effects, MOMP is often considered the point of no return in the apoptotic program. Mitochondrial apoptosis plays numerous important pathophysiological roles. In cancer, inhibition of apoptosis both promotes tumorigenesis and impedes anti-cancer therapeutic efficacy (Delbridge et al., 2012). Apoptotic inhibition is often achieved by upregulation of anti-apoptotic BCL-2 family members that prevent MOMP. This has led to the development of new anticancer drugs, called BH3-mimetics, which neutralize anti-apoptotic BCL-2 function (Ni Chonghaile and Letai, 2008).

Live-cell imaging has demonstrated that mitochondrial permeabilization is often an all-or-nothing event (Goldstein et al., 2000). Widespread mitochondrial permeabilization underpins the lethal effects of MOMP by ensuring robust caspase activity, or in its absence, massive mitochondrial dysfunction. In some limited circumstances, cells can survive MOMP. For example, growth factor-deprived neurons can survive MOMP due to a failure to properly engage caspase activity (Deshmukh and Johnson, 1998; Martinou et al., 1999; Wright et al., 2004). In proliferating cells, expression of the key glycolytic enzyme GAPDH can promote cell survival following MOMP provided caspase activity is inhibited (Colell et al., 2007). We have previously found that the ability of cells to survive MOMP depends on a few mitochondria that evade permeabilization and re-populate the cell (Tait et al., 2010).

Whereas earlier studies demonstrated that strong pro-apoptotic stimuli lead to rapid, synchronous, and complete MOMP, technical limitations have made it impossible to study the effects of sub-lethal stresses on individual mitochondria. Here, we use newly developed imaging techniques to

demonstrate that MOMP can occur in a limited subset of mitochondria following a sub-lethal stress. Crucially, this limited MOMP leads to caspase activation, which, while insufficient to trigger cell death, leads to limited cleavage of key caspase substrates. This in turn drives DNA-damage and genomic instability, promoting transformation and tumorigenesis. Importantly, our data argue that the mitochondrial apoptotic pathway may exert either a tumor suppressor or oncogenic function depending upon the extent of MOMP.

RESULTS

Limited Mitochondrial Permeabilization Occurs in the Absence of Cell Death

Mitochondrial permeabilization during apoptosis is widespread such that most or all mitochondria within a cell undergo MOMP; this effectively commits a cell to die. However, the potential for sub-lethal apoptotic stresses to engage MOMP in a limited number of mitochondria has not been tested. To investigate this, we used ABT-737, the prototypic BH3-mimetic compound that sensitizes to apoptosis by antagonizing anti-apoptotic BCL-2 family proteins (Oltersdorf et al., 2005). HeLa or U2OS cells were treated with varying concentrations of ABT-737, enantiomer (less-active stereoisomer of ABT-737) or the apoptosis-inducer staurosporine (STS) and analyzed for apoptosis by Annexin V staining and flow cytometry. Importantly, whereas STS triggered apoptosis, treatment with ABT-737 at varying doses failed to induce detectable apoptosis (Figure 1A). Similarly, live-cell imaging using the cell impermeable dye Sytox green also failed to reveal a cytotoxic effect of ABT-737 treatment (Figure S1A). Finally, BH3-mimetic treatment at the indicated doses had no effect on long-term cell survival as determined by clonogenic assay (Figure S1B). We next asked if mitochondrial permeabilization occurred following this non-lethal BH3-mimetic treatment. HeLa cells were treated with ABT-737 or, as a positive control, Actinomycin D (Act D) and cytosolic fractions were probed for the presence of cytochrome *c* to detect MOMP. As expected, Act D treatment led to MOMP as demonstrated by the detection of cytochrome *c* in the cytosolic extract (Figure 1B). Surprisingly, treatment with a non-lethal dose of ABT-737 also led to low, but detectable levels of MOMP, implying that MOMP could be engaged without killing the cell (Figure 1B).

In caspase-proficient cells, complete MOMP invariably represents a point of no return; we therefore reasoned that MOMP could only be non-lethal if it occurred in a minority of mitochondria (called hereafter minority MOMP). To test this possibility, we developed a new approach to specifically visualize permeabilized mitochondria using fluorescent protein re-localization and chemically dimerizable FKBP/FRB domains (Belshaw et al., 1996). Two fluorescent probes were constructed: a cytosolic probe comprising of GFP fused to a FKBP domain (cytoGFP) and a mitochondrial targeted probe (mitoCherry) comprising of mCherry fused to an FRB domain and the mitochondrial anchoring sequence of Apoptosis Inducing Factor (AIF) (Otera et al., 2005). In the presence of chemical heterodimerizer (A/C heterodimerizer, AP21967), the two probes can only co-localize on mitochondria following MOMP when cytoGFP can gain access to the mitochondrial inner membrane (Figure 1C). To validate this method, we treated U2OS cells with the apoptotic stimulus Act D. Importantly, Act D treatment led to robust mitochondrial re-localization of cytoGFP only in the presence of dimerizer that, as expected, was prevented by blocking MOMP by expression of BCL-xL (Figures 1D, S1C, and S1D). In line with this method marking MOMP as the key initiating apoptotic event, cytoGFP re-localization preceded caspase dependent apoptotic effects including cell shrinkage, rounding, and plasma membrane blebbing (Movie S1). Further verifying this technique, Act D-induced mitochondrial localization of GFP was only observed in cells in which MOMP had occurred, as demonstrated by the cytosolic release of Smac mCherry (a verified reporter of MOMP) (Figure S1E and Movie S2) (Tait et al., 2010). These fluorescent tools thus allow us to detect the permeabilization of individual mitochondria, and thereby assay for the presence of minority MOMP.

Using this approach, we investigated the extent of MOMP following sub-lethal apoptotic stimuli. Strikingly, following ABT-737 treatment, we were able detect MOMP in a limited number of mitochondria in both HeLa and U2OS cells (Figure 1E). Confirming their permeabilization, mitochondria with relocalized CytoGFP had also released Smac-mCherry and cytochrome *c* (Figures S1F and S1G). The percentage of cells displaying minority MOMP increased in a dose-dependent and BCL-xL inhibitable manner following ABT-737 treatment (Figure 1F). Importantly, using live-cell imaging, cells displaying minority MOMP failed to undergo cell death during extended periods of analysis (Figure S1H). Collectively, these data demonstrate that MOMP can occur in a limited number of mitochondria in response to ABT-737 treatment without leading to cell death.

Minority MOMP Engages Sub-Lethal Caspase Activity

We next sought to understand the consequences of minority MOMP, and in particular, whether it might lead to activation of caspases at sub-apoptotic levels. We first quantified the extent of minority MOMP in HeLa cells. This revealed an average of 2.5% of a cell's mitochondria undergoing permeabilization following sub-lethal ABT-737 treatment (Figure 2A). Together with previously published criteria, this allowed us to adapt a mathematical HeLa cell model of the apoptosis execution phase (Rehm et al., 2006) to perform in silico simulations of the consequences of minority MOMP on the efficiency of caspase-3 processing and activation. Importantly, despite the presence of amplifying feedback loops which ensure rapid and full caspase activation in response to regular MOMP, simulations for minority MOMP conditions demonstrate that caspase-3 would be processed and activated sub-maximally and therefore, potentially, at sub-lethal levels (Figures 2B and S2A). To experimentally verify this, U2OS and HeLa cells were treated with the BH3-mimetic ABT-737 in the presence or absence of caspase inhibitor quinolyl-valyl-O-methylaspartyl-[2,6-difluoro- phenoxy]-methyl ketone (Q-VD-OPh). Treatment with a wide range of sub-lethal doses of ABT-737 triggered caspase activity as evidenced by pro-caspase-3 processing and PARP cleavage, effects that were blocked by caspase inhibition (Figures S2B and S2C). We next compared caspase activity between conditions that engage minority MOMP and apoptotic conditions. Cells were treated with ABT-737 to induce minority MOMP or with TNF/CHX or Act D to engage apoptosis (Figures 2C–2E and S2D). We determined

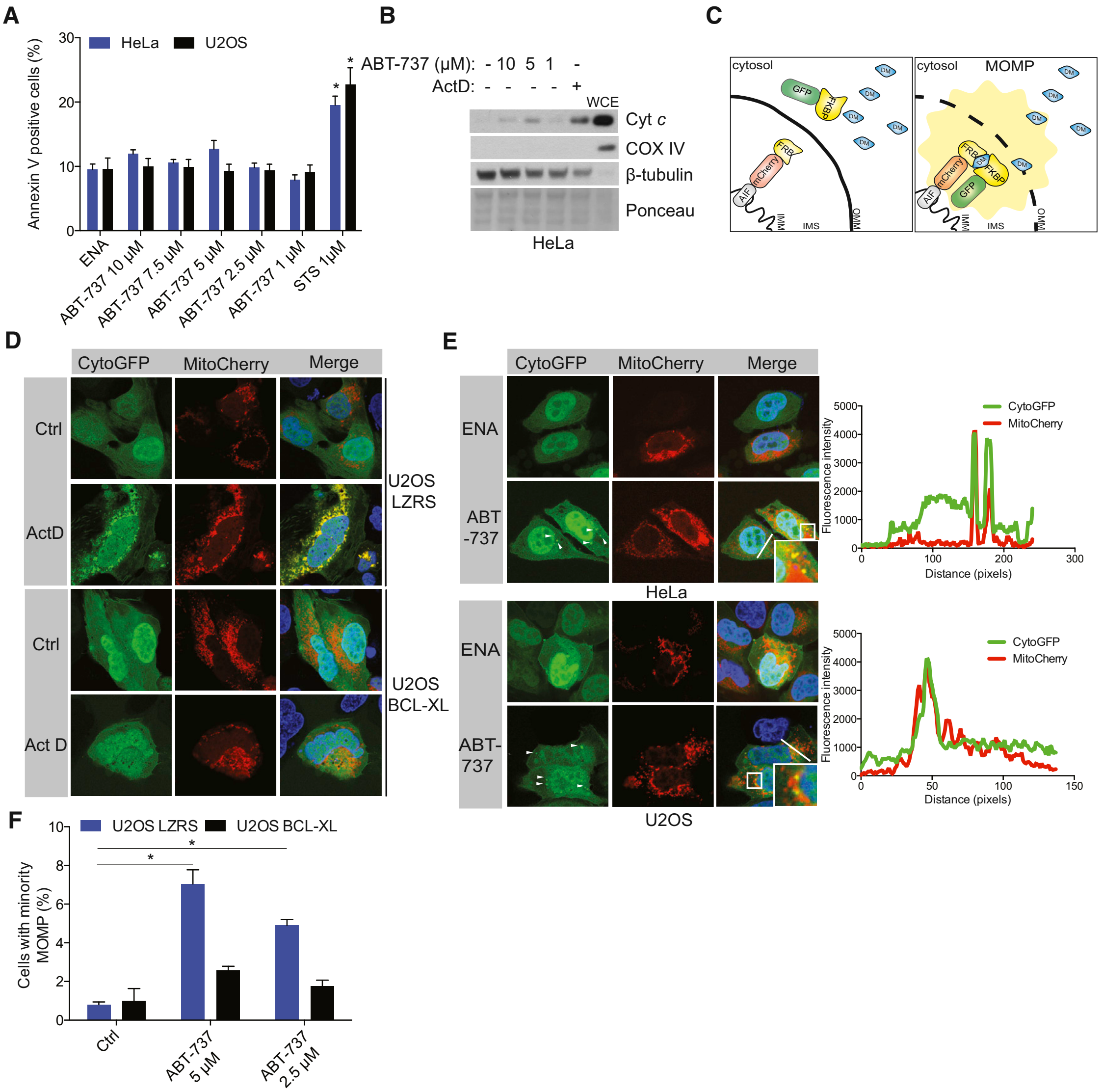

Figure 1. Limited Mitochondrial Outer Membrane Permeabilization Occurs without Triggering Cell Death

(A) HeLa and U2OS cells were treated for 3 hr with different concentrations of ABT-737 or enantiomer (ENA, 10 μM) or with staurosporine (STS, 1 μM) for 12 hr and analyzed by flow cytometry for Annexin V-positive cells. Data represent mean ± SEM of three independent experiments.

(B) HeLa cells were treated for 3 hr with different concentrations of ABT-737 or actinomycin D for 6 hr (Act D, 1 μM), and cytosolic extracts were western blotted for cytochrome *c*, COX IV, and β-tubulin. WCE, whole-cell extract.

(C) Schematic representation of GFP relocalization-based MOMP detection method. DM, chemical heterodimerizer; IMM, inner mitochondrial membrane; IMS, intermembrane space; OMM, outer mitochondrial membrane.

(D) U2OS cells expressing vector or BCL-xL together with CytoGFP/MitoCherry were treated with Act D (1 μM) for 3 hr in the presence of heterodimerizer and imaged by confocal microscopy.

(E) HeLa or U2OS cells expressing CytoGFP/MitoCherry were treated with vehicle or ABT-737 (5 μM) or enantiomer (5 μM, ENA) for 3 hr and imaged by confocal microscopy. Arrows denote permeabilized mitochondria. Line scans represent variation in red and green fluorescence intensity along the denoted line.

(F) U2OS cells expressing CytoGFP/MitoCherry were treated for 3 hr with ABT-737, and minority MOMP was quantified. Data represent mean ± SEM of three independent experiments.

*$p < 0.05$, compared versus control. See also Figure S1 and Movies S1 and S2.

executioner caspase-3 and -7 activity, by detection of their active, cleaved fragment (Figure 2C) or by their activity-dependent precipitation using biotin-Val-Ala-Asp-Fluoromethyl Ketone (b-VAD) (Figure 2D). Both approaches demonstrated that caspase-3 and -7 activity was detectable but significantly less in ABT-737 treated cells undergoing minority MOMP compared with apoptotic cells (Figures 2C and 2D). Levels of active caspase-9, precipitated with b-VAD, were also detectable but significantly less in ABT-737 treated cells undergoing minority MOMP in comparison to apoptotic cells (Figure 2E). In line with ABT-737

activating the mitochondrial caspase pathway, ABT-737 treatment led to caspase-9 but not caspase-8 activation (Figure 2E). Collectively, these data argue that minority MOMP can engage sub-lethal caspase activity. To corroborate these findings, we used a recently developed caspase reporter protein (VC3AI) that fluoresces following caspase-mediated cleavage (Zhang et al., 2013). As validation, apoptotic treatments led to an increase in fluorescence in VC3AI expressing HeLa cells in a caspase-dependent manner, whereas cells expressing the non-cleavable control (ncVC3AI) remained non-fluorescent (Figure S2E). Significantly, flow cytometry analysis demonstrated that ABT-737 treatment led to a detectable increase in caspase activity in viable cells that was inhibited by the caspase inhibitor Q-VD-OPh, further supporting the hypothesis that minority MOMP triggers sub-lethal caspase activation (Figure 2F). We then treated HeLa cells co-expressing Smac-mCherry together with either VC3AI or ncVC3AI with the BH3-mimetic ABT-737. In line with the flow cytometry data, treatment with ABT-737 specifically increased the percentage of cells displaying weak caspase-dependent fluorescence (Figures 2G, 2H, and S2F). Importantly, cells exhibiting caspase-activity failed to display apoptotic, widespread MOMP because Smac-mCherry remained localized in the mitochondria (Figure 2G). We next determined if cells displaying sub-lethal caspase activity could survive long-term. HeLa cells expressing the VC3AI reporter were treated either with ABT-737 to engage minority MOMP dependent caspase activity or Act D to trigger mitochondria-dependent apoptosis. Equal numbers of caspase active (GFP-positive cells) from both conditions were sorted and compared for clonogenic survival versus caspase inactive, GFP-negative cells (Figures 2I and S2G). Importantly, similar clonogenic survival was observed comparing ABT-737 treated caspase active versus inactive cells; in contrast, stimulation of mitochondrial dependent apoptosis by Act D prevented clonogenic outgrowth (Figure 2I). Taken together, these data demonstrate that minority MOMP can engage low level caspase activity under which cells can survive.

Minority MOMP Induces Caspase-Dependent DNA Damage

DNA fragmentation is a classical apoptotic hallmark mediated by caspase-activated DNase (CAD) (Sakahira et al., 1998). We hypothesized that limited caspase activity following minority MOMP might lead to low-level CAD activation, and, in turn, to induction of DNA damage in surviving cells. To test this, we treated HeLa and U2OS cells with ABT-737 in the presence or absence of Q-VD-OPh and analyzed for γH2A.X as readout for DNA damage. Importantly, in both cell lines, non-lethal treatment with BH3 mimetic led to caspase-dependent DNA damage as demonstrated by a caspase-dependent increase in γH2A.X (Figure 3A). Importantly, the extent of DNA-damage (measured by γH2A.X foci) correlated with minority MOMP, implicating a causal relationship between the two (Figures 3B and 3C). The ability of BH3 mimetics to engage DNA-damage in a caspase-dependent manner was also observed in other cell lines (Figure S3A). Further demonstrating caspase-dependent DNA damage, ABT-737 treatment also led to an increase in DNA breaks, measured by comet assay and Ser^{15} p53 phosphorylation dependent on caspase function, mirroring γH2A.X levels (Figures 3D, 3E, and S3B).

Although p53 independent, induction of DNA damage depended on mitochondrial caspase activation, because overexpression of BCL-xL (HeLa), deletion of Bax (HCT-116) or Bax and Bak in murine embryonic fibroblasts (MEF), or knockdown of APAF-1 (HeLa) prevented BH3 mimetic-induced γH2A.X (Figures 3F–3H and S3C–S3E). Supporting these findings, direct, non-lethal activation of caspase-9 by chemical dimerization also led to DNA damage (Figures 3I and S3F).

Because these data clearly demonstrate caspase-dependent activation of a DNA damage response, we next tested whether this effect is mediated by CAD activation. Caspase-3 activity is required for CAD activation through cleavage of its inhibitor ICAD. Consistent with caspase-3 and CAD-dependent DNA damage downstream of minority MOMP, γH2A.X was not observed following ABT-737 treatment of caspase-3 deficient MCF-7 cells in contrast to caspase-3 reconstituted MCF-7 (Figure S3G). To directly test the involvement of CAD in minority MOMP-induced DNA damage, we used CRISPR/Cas9 genome editing to generate CAD-deficient HeLa and U2OS cells (Figure 3J). In both cell lines, CAD deletion prevented BH3 mimetic induction of γH2A.X, demonstrating its requirement for minority MOMP-induced DNA damage (Figure 3J). Similar results were observed following CAD knockdown by siRNA (Figure S3H). In line with these findings, CAD deletion also effectively prevented ABT-737 induced DNA breaks, as determined by comet assay (Figure 3K). Furthermore, we also found that ICAD is cleaved following induction of minority MOMP, thereby supporting CAD activation (Figure S3I). Finally, we addressed if minority MOMP also promotes DNA damage in vivo. Mice were administered one dose of ABT-737 (either 75 mg/kg or 125 mg/kg) or administered daily with ABT-737 (75 mg/kg) over a 3-day period. Strikingly, a single dose of ABT-737 resulted in a significant increase of γH2A.X immunoreactivity in the small intestine of mice (Figure 3L). Repeated dosing did not result in an increase in γH2A.X immunoreactivity, potentially due to resolution of DNA damage in between doses (Figure S3J). Although not an absolute measure of apoptosis, because it detects cells only transiently during apoptosis, TUNEL staining failed to reveal any evidence of apoptosis following all ABT-737 treatments (Figures 3L and S3J). Collectively, these data show that sub-lethal stresses, causing minority MOMP, can trigger caspase-dependent DNA damage both in vitro and in vivo.

Sub-Lethal BH3-Only and Apoptotic Stress Induce Minority MOMP and DNA Damage

Although our data demonstrate that the BH3 mimetic drug ABT-737 triggers minority MOMP and DNA damage, we sought to demonstrate that these effects also occur following MOMP triggered through other means. BH3-only proteins are the endogenous inducers of MOMP through their ability to activate Bax and Bak. Therefore, we next asked whether BH3-only proteins themselves could also engage minority MOMP. For this purpose, we generated a MelJuSo cell line expressing the BH3-only protein tBID under a doxycycline inducible promoter. Whereas doxycycline addition at 1 μg/ml led to robust tBID expression and apoptotic cell death, we were able to titrate doxycycline down to induce non-lethal tBID expression (Figures 4A, 4B, and S4A–S4D). We assessed whether expression of tBID at non-lethal

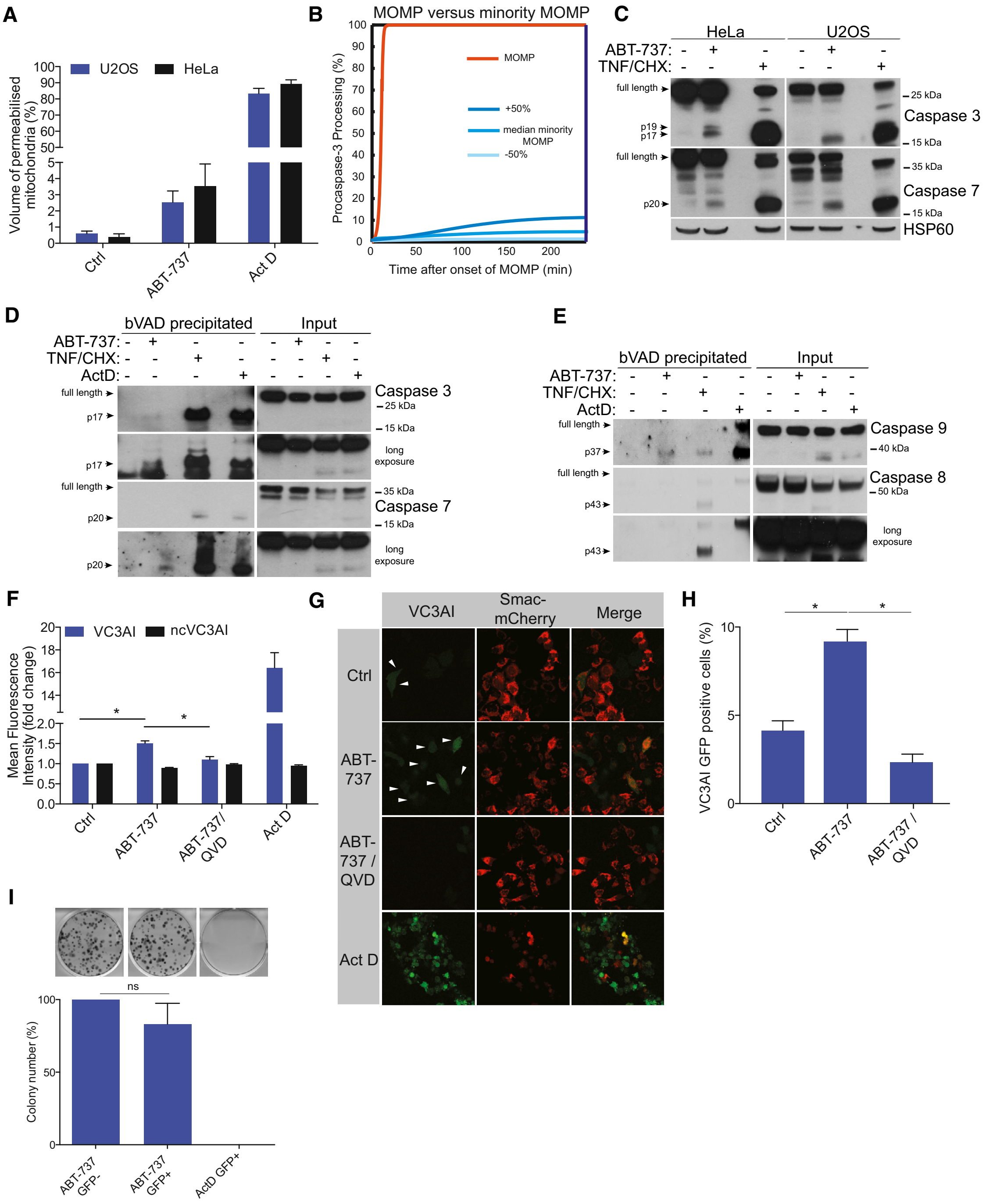

Figure 2. Minority MOMP Engages Sub-Lethal Caspase Activity

(A) U2OS and HeLa cells expressing CytoGFP/MitoCherry were treated or not with ABT-737 (10 μM for 3 hr in the presence of dimerizer) to induce minority MOMP, and then z stack confocal imaging was performed. Act D (1 μM) was used to induce complete MOMP. Mitochondrial volume was measured using ImageJ. Data represent mean of permeabilized mitochondrial volume ± SEM from ten cells per condition.

(B) Data from (A) were used as inputs into a mathematical HeLa cell model of apoptosis execution signaling to calculate the consequences of minority MOMP on the efficacy of procaspase-3 processing.

(C) HeLa and U2OS cells were treated with ABT-737 (10 μM) or TNF/CHX (20 ng/ml TNF and 1 μg/ml CHX) for 3 hr, and cell extracts were western blotted for caspases-3 and -7.

(D) Biotinylated-VAD-FMK (bVAD) was incubated with HeLa cells for 1 hr following 3 hr treatments with the indicated stimuli. Cell lysates and precipitated proteins were western blotted for caspases-3 and -7.

(legend continued on next page)

levels could also trigger minority MOMP. Importantly, sub-lethal amounts of tBID (induced by 2.5 and 1 ng/ml doxycycline) led to minority MOMP as detected by the presence of cytochrome *c* in the cytosol (Figure 4C). We aimed to validate these findings using our method to detect MOMP via GFP re-localization. Non-lethal levels of tBID expression led to a clear increase in cells displaying minority MOMP in a manner that could be prevented through co-expression of BCL-xL (Figures 4D and 4E). Importantly, as was observed for BH3-mimetic treatment, the caspase inhibitor Q-VD-OPh also prevented H2A.X phosphorylation upon induction of sub-lethal levels of tBID (Figure 4F). We next addressed if a physiological apoptotic stimulus could also trigger minority MOMP and DNA-damage. Accordingly, sub-lethal treatment of U2OS cells with FAS ligand triggered minority MOMP and DNA-damage in a BCL-xL and caspase-inhibitable manner (Figures 4G, 4H, and S4E). Similarly, sub-lethal treatment of cells with the proteasome inhibitor MG132 also induced minority MOMP (Figure 4I). Collectively, these data demonstrate that similar to BH3-mimetics, apoptotic stimuli and pro-apoptotic BH3-only proteins also induce minority MOMP and DNA damage.

JNK Regulates the DNA Damage Response

DNA damage-induced phosphorylation of H2A.X at S139 often occurs via the PI3K-related kinase family members ATM, ATR, and DNA-PK (Jackson and Bartek, 2009; Shiloh, 2003). However, we did not observe any significant increase in activated, phosphorylated ATM or ATR kinase following sub-lethal ABT-737 treatment (Figure 5A). Moreover, RNAi-mediated knockdown of ATM, ATR, or DNA-PK failed to affect BH3 mimetic-induced γH2A.X levels (Figures S5A and S5B). Besides ATM, ATR, and DNA-PK, c-Jun N-terminal kinase (JNK) has also been found to mediate H2A.X phosphorylation in some settings (Lu et al., 2006). Importantly, sub-lethal treatment with ABT-737 led to a caspase-dependent increase in JNK1/2 activation mirroring levels of γH2A.X (Figure 5B). JNK activation following ABT-737 administration was also detected in vivo in the small intestine (Figure 5C). To directly investigate the role of JNK in H2A.X phosphorylation, we used RNAi. Combined knockdown of JNK1/2 or selective knockdown of JNK2 effectively prevented ABT-737 induced γH2A.X implicating a direct role for JNK2 in H2A.X phosphorylation (Figures 5D and 5E). Accordingly, RNAi-mediated knockdown of CAD largely inhibited JNK1/2 phosphorylation (Figure 5F). These results identify JNK2 as a key player in the minority MOMP-induced DNA damage response.

Minority MOMP Promotes Genomic Instability

Based on our results, we hypothesized that by causing DNA damage, minority MOMP may promote genomic instability and transformation. To test this possibility, we repeatedly treated HeLa and U2OS cells with sub-lethal doses of ABT-737 for five (P5) or ten passages (P10). Following blockage of cytokinesis, we then quantified the number of cells with micronuclei, a well-established marker for chromosomal damage (Figure S6A) (Fenech, 2007). Strikingly, U2OS and HeLa cells displayed a significant increase in micronuclei number following ABT-737 treatment in a dose-dependent manner (Figures 6A and S6B). Ectopic BCL-xL expression inhibited micronuclei accumulation in U2OS cells, confirming that the observed genomic instability required mitochondrial permeabilization (Figure 6B). In an analogous manner, induction of sub-lethal levels of the BH3-only protein tBID in MelJuSo cells also promoted micronuclei accumulation in a dose-dependent manner (Figure 6C). Micronuclei accumulation following tBID expression was reduced to control levels by the caspase inhibitor Q-VD-OPh, demonstrating a requirement for caspases in minority MOMP-induced chromosomal damage (Figure 6C).

DNA damage can also lead to gene amplification, a deleterious event that contributes to oncogene activation. We used the PALA assay to test if minority MOMP can trigger gene amplification (Mathew et al., 2009). PALA (N-phosphonoacetyl-L-aspartate) prevents pyrimidine synthesis by inhibiting the CAD enzyme (carbamyl phosphate synthetase/aspartate transcarbamylase/dihydro-orotase; note that this enzyme shares an abbreviation with, but is distinct from, caspase-activated DNase discussed previously). Resistance to PALA treatment in murine cells is mediated solely through *Cad* gene amplification. Various murine cell lines (PDAC, 3T3-SA, and WEHI-S) were repeatedly treated with BH3 mimetic, then assayed for amplification of the *Cad* locus and resistance to PALA treatment. Crucially, ABT-737 treatment promoted clonogenic survival in all tested murine cell lines following PALA treatment (Figures 6D and S6C), consistent with genome instability-driven *Cad* locus amplification. In line with this result, qPCR revealed that ABT-737 treatment induced *Cad* gene amplification in all three cell lines (Figure 6E). Importantly, BCL-xL overexpression in PDAC cells inhibited ABT-737-induced PALA resistance and *Cad* gene amplification, implicating a key role of minority MOMP in gene amplification (Figures 6F–6H, S6D, and S6E). Collectively, these results demonstrate that minority MOMP causes genomic instability.

(E) As in (D), except HeLa cells were pre-incubated for 1 hr with bVAD and treated for 3 hr with ABT-737 (10 μM) or 16 hr Act D (16 hr, 1 μM) or 3 hr TNF/CHX (20 ng/ml TNF and 1 μg/ml CHX). Proteins were precipitated with neutravidin agarose resin. Cell lysates and precipitated proteins were western blotted for caspases-8 and -9.

(F) HeLa cells stably expressing the caspase activity reporter (VC3AI) or non-cleavable control (ncVC3AI) were treated for 24 hr with 10 μM ABT-737 in presence or absence of Q-VD-OPh (10 μM). GFP mean fluorescence intensity of the viable cells was quantified by flow cytometry. Results represent the fold increase in fluorescence over control. Data represent mean ± SEM of four independent experiments.

(G) HeLa cells stably expressing VC3AI together with Smac-mCherry were treated as in (F) and imaged for GFP. Arrows denote caspase reporter (GFP)-positive cells.

(H) Quantification of percentage of GFP-positive cells following ABT-737 (10 μM) treatment in the presence or absence of Q-VD-OPh (10 μM). Data represent mean ± SEM of three independent experiments.

(I) HeLa VC3AI cells were treated with ABT-737 (10 μM) for 24 hr or Act D (0.5 μM), and equal numbers of GFP-positive cells (ABT-737 and Act D treated sample) and GFP-negative cells (ABT-737 treated cells) were sorted by flow cytometry and assessed for clonogenic survival. Data represent mean ± SEM of three independent experiments.

*$p < 0.05$, compared versus control. See also Figure S2.

CellPress

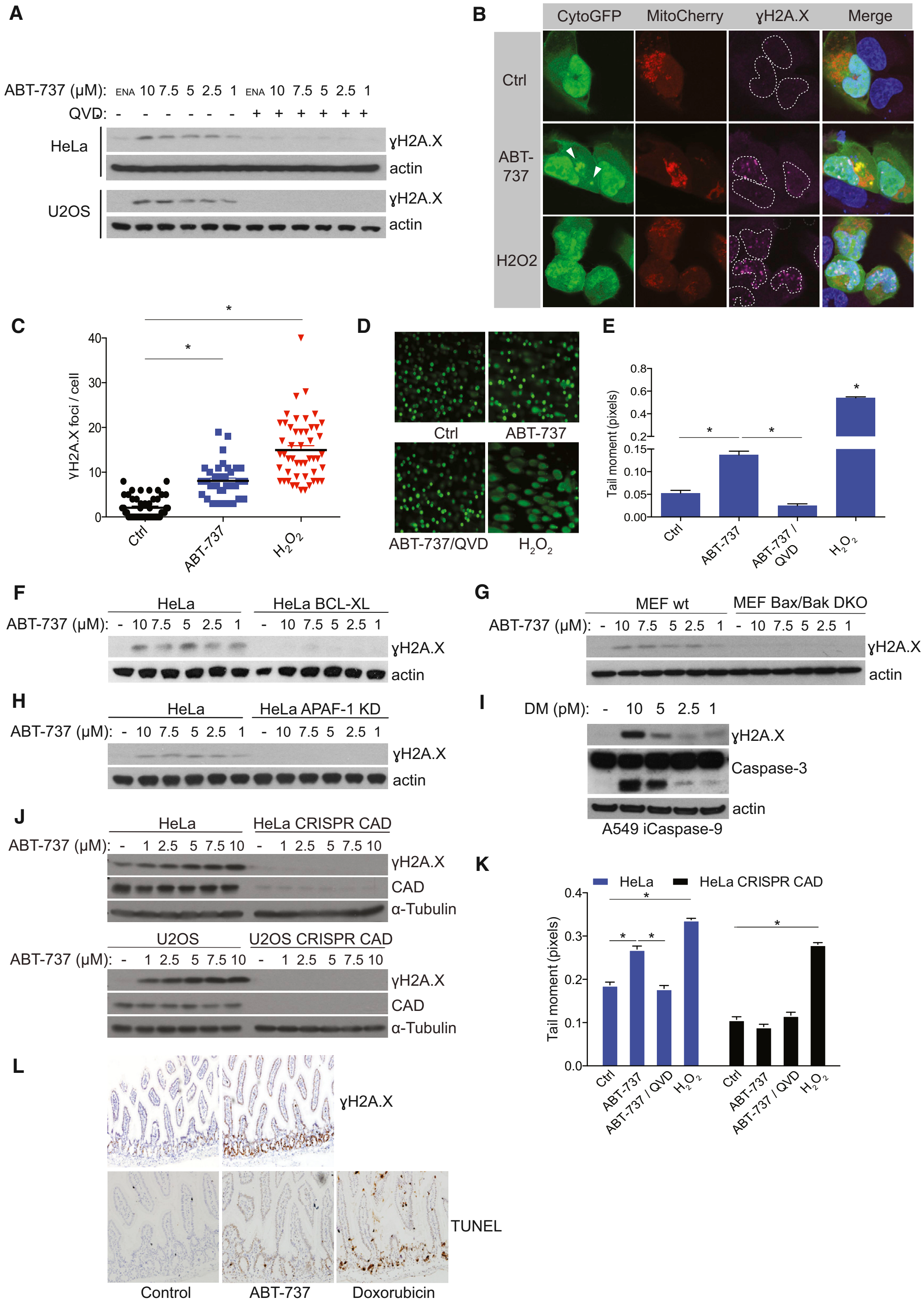

Figure 3. Minority MOMP Induces Caspase-Dependent DNA Damage

(A) HeLa and U2OS cells were treated for 3 hr with indicated sub-lethal doses of ABT-737 or enantiomer (10 μM, ENA) in presence or absence of caspase inhibitor Q-VD-OPh (10 μM). Cell lysates were for probed by western blot for γH2A.X and actin (as loading control).

(legend continued on next page)

Minority MOMP Promotes Transformation and Tumorigenesis

Genomic instability can promote oncogenic transformation. Therefore, we investigated if minority MOMP-induced DNA damage leading to genomic instability could potentiate E1A/KRAS-induced transformation in primary mouse embryonic fibroblasts (MEF). Primary MEFs were treated with sub-lethal doses of ABT-737 prior to transformation with E1A/KRAS. Remarkably, BH3-only stress was able to potentiate the transforming ability of E1A/KRAS in primary cells in a dose-dependent manner (Figures 7A and 7B). We next tested if minority MOMP could promote transformation in a different setting. Primary MEF deficient in the tumor suppressor $p19^{Arf}$ were treated with enantiomer or ABT-737 in the presence or absence of caspase-inhibitor Q-VD-OPh (Figure S7A). Additionally, primary $p19^{Arf}$ deficient MEF expressing BCL-xL or empty vector were treated with ABT-737 (Figures S7B and S7C). Following treatment, cellular transformation was assessed by anchorage-independent growth in soft agar. Strikingly, ABT-737 treatment led to transformation that was completely prevented by inhibiting caspases (with Q-VD-OPh) or by inhibiting MOMP through BCL-xL expression (Figures 7C and 7D). We next compared ABT-737 or enantiomer treated $p19^{Arf}$ null MEF for their tumorigenic potential in vivo following subcutaneous injection in CD1-*Nude* mice. Significantly, ABT-737 treated MEF formed tumors much more rapidly than enantiomer-treated MEF (Figure 7E). Collectively, these results demonstrate that minority MOMP promotes both cellular transformation and tumorigenesis.

DISCUSSION

Apoptosis plays multiple beneficial roles in cancer, acting as both a potent tumor suppressor and therapeutic effector mechanism. Conversely, various reports also suggest an oncogenic function for apoptosis in some settings. For example, overexpression of anti-apoptotic BCL-2 proteins confers a favorable prognosis in certain cancer types whereas genetic deletion of the pro-apoptotic BH3-only proteins BID and PUMA inhibits tumorigenesis in some settings (Berardo et al., 1998; Biswas et al., 2013; Labi et al., 2010; Michalak et al., 2010; Reed, 1996). Moreover, caspase-dependent DNA damage has been described following mitotic slippage and treatment with the death-receptor ligand TRAIL (Lovric and Hawkins, 2010; Orth et al., 2012). Finally, following ethanol exposure, recovery from apoptosis is associated with genomic instability and transformation, although it remains unclear whether apoptosis has a direct causative role in this setting (Tang et al., 2012).

Our data demonstrate that by limited mitochondrial permeabilization, the same core apoptotic machinery that protects from cancer can now lead to caspase-dependent genomic instability and promote transformation. We find that the ability of minority MOMP to stimulate DNA damage requires mitochondrial-dependent caspase activation of the DNase CAD. Our finding that repeated engagement of minority MOMP triggers genomic instability may be due to an excessive induction of DNA damage and/or additionally a simultaneous inactivation of key DNA damage response (DDR) pathways. Accordingly, we observe sub-lethal caspase activation suffices to cleave PARP1, an important player in the DDR, as well as ATM and KAP1 (data not shown) both of have been shown or are predicted to be inactivated following caspase-mediated cleavage (Smith et al., 1999; Van Damme et al., 2005). Inactivation of ATM may also explain the requirement for JNK in mediating γH2A.X phosphorylation following CAD activity. Potentially, inactivation of additional DDR pathways may exacerbate DNA-damage and genomic instability initiated by minority MOMP.

Minority MOMP-induced DNA damage may play various important roles in cancer. For example, physiological triggers of mitochondrial apoptosis that fail to trigger cell death could promote genomic instability and cancer. Further studies investigating the impact of CAD deletion upon transformation and tumorigenesis will help shed light on this. The ability of anti-cancer therapies to trigger minority MOMP-induced DNA damage may have negative consequences. We find that the BH3 mimetic ABT-737 can induce DNA damage and genomic instability at doses comparable to those achieved during clinical application of BH3 mimetics. In combination with oncogenic stress or tumor suppressor loss, ABT-737 treatment promoted transformation. In cancer therapy, this raises a cautionary paradox, namely that treating tumor cells with an apoptosis-inducing therapy (such as BH3-mimetics) may in itself be oncogenic. Supporting this, etoposide-induced chromosomal translocations driving therapy-associated leukemia have previously been shown to require caspase and CAD activity; this possibly requires minority MOMP to initiate caspase activation (Hars et al., 2006; Sim and Liu, 2001). Second, minority MOMP-induced DNA damage that is engaged by pro-apoptotic

(B) U2OS cells transiently expressing CytoGFP and MitoCherry were treated with ABT-737 (5 μM) for 3 hr or H_2O_2 (25 μM) for 10 min and immunostained for γH2A.X. Representative images are shown.

(C) Quantification of γH2A.X foci in cells displaying minority MOMP (ABT-737-treated cells), control, and H_2O_2-treated cells. Data represent mean ± SEM of three independent experiments.

(D) HeLa cells were treated as in (B) and subject to comet assay. Representative images are shown.

(E) Quantification of comet tail moment following ABT-737 treatment. Data represent mean ± SEM of three independent experiments.

(F–H) HeLa and HeLa overexpressing BCL-xL (F), wild-type MEF and MEF double knockout for Bax and Bak (G), or HeLa versus HeLa knockdown for APAF-1 (H) were treated as in (A) and western blotted for γH2A.X and actin.

(I) A549 cells expressing caspase-9 fused to a FKBP dimerization domain were treated with indicated sub-lethal concentrations of homodimerizer (DM) for 3 hr to induce caspase-9 dimerization and activation. Cleavage of caspase-3 and γH2A.X was assessed by western blot.

(J) Wild-type HeLa and U2OS cells and their *Cad*-deleted counterparts were treated and immunoblotted as in (A).

(K) Wild-type and *Cad*-deleted HeLa cells were treated as in (D) and used to perform comet assay. Graph represents quantification of comet tail moment. Data represent mean ± SEM of four independent experiments.

(L) Representative images of γH2A.X and TUNEL immunohistochemical staining in small intestine of mice treated with ABT-737 (75 mg/kg) for 1 day (n = 3).

*$p < 0.05$, compared versus control. See also Figure S3.

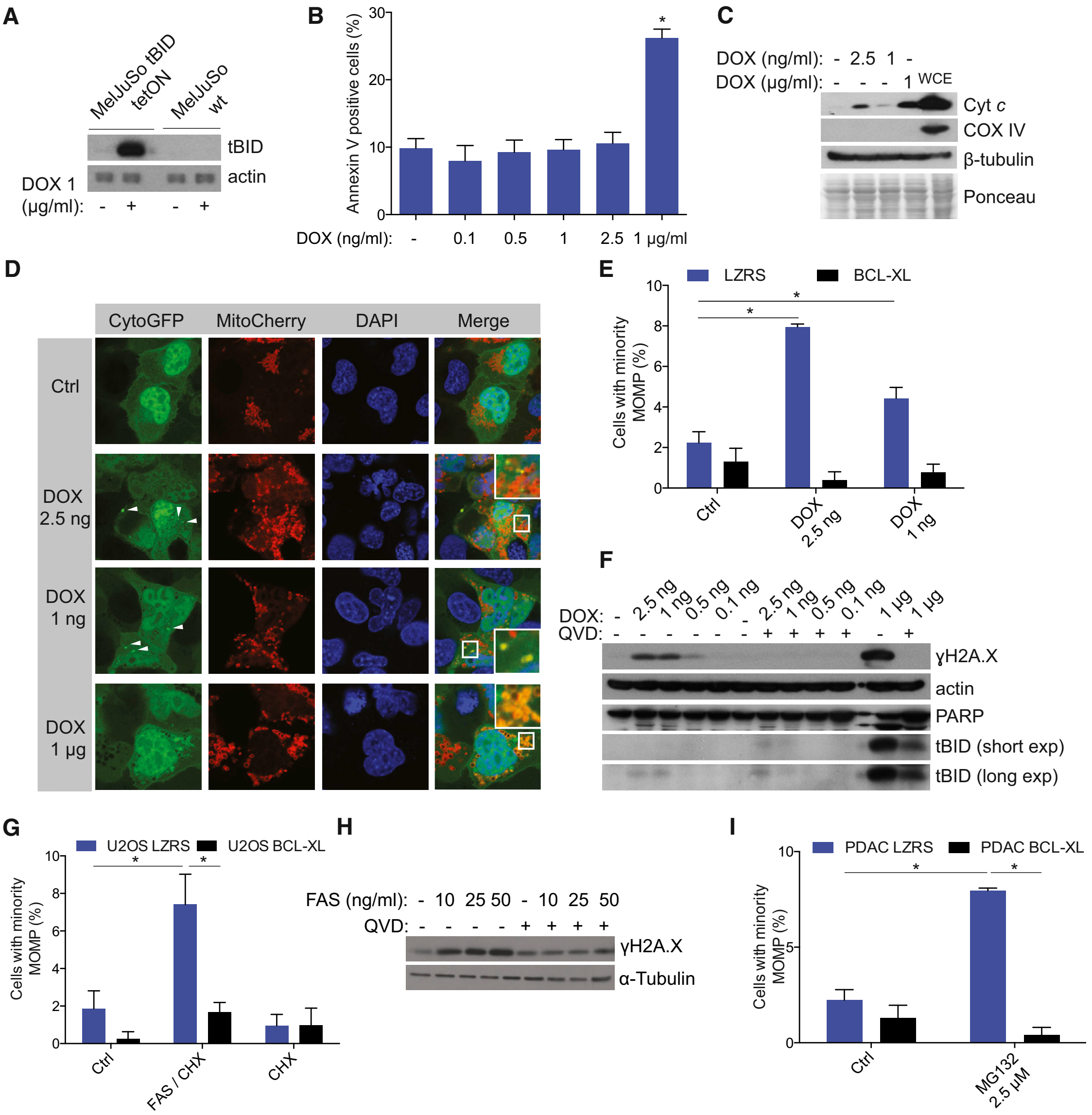

Figure 4. Sub-Lethal BH3-Only Protein and Apoptotic Stress Induces Minority MOMP and DNA Damage

(A) MelJuSo tBID tetON or wild-type cells were treated for 12 hr with 1 µg/ml of doxycycline (DOX), and cell lysates were probed for tBID.

(B) MelJuSo tBID tetON cells were treated for 6 hr with DOX and cell viability was assessed by Annexin V-based flow cytometry. Data represent mean ± SEM of three independent experiments. *$p < 0.05$, compared to control.

(C) Cytosolic fractions from MelJuSo tetON tBID cells treated as in (B) were probed for cytochrome *c*. To induce apoptosis, 1 µg/ml DOX was used as a positive control. WCE, whole-cell extract.

(D) MelJuSo tBID tetON expressing CytoGFP/MitoCherry were treated with DOX as in (A) and imaged by confocal microscopy. Arrows denote mitochondria undergoing permeabilization.

(E) Quantification of cells undergoing minority MOMP. Data represent mean ± SEM of three independent experiments.

(F) MelJuSo tBID tetON were treated with DOX as in (A) and cell lysates were probed by western blot for γH2A.X, PARP, and tBID.

(G) U2OS cells stably expressing empty vector or BCL-xL were treated for 3 hr with FAS ligand (10 ng/ml) and CHX (1 µg/ml) and scored for the presence of minority MOMP. Data represent mean ± SEM of three independent experiments.

(H) U2OS cells were treated for 3 hr with the indicated concentrations of FAS ligand and CHX (1 µg/ml), and western blot was performed for γH2A.X.

(I) PDAC cells were treated with MG132 (2.5 µM) for 3 hr, and minority MOMP was quantified. Data represent mean ± SEM of three independent experiments. *$p < 0.05$, compared versus control. See also Figure S4.

anti-cancer therapies has the potential to be mutagenic. This could contribute to increased aggressiveness of relapsing tumors following treatment and to the acquisition of drug resistance. Indeed, acquired resistance to the BH3 mimetic ABT-199 was recently shown to be due to ABT-199-induced missense mutations in *BCL-2* (Fresquet et al., 2014).

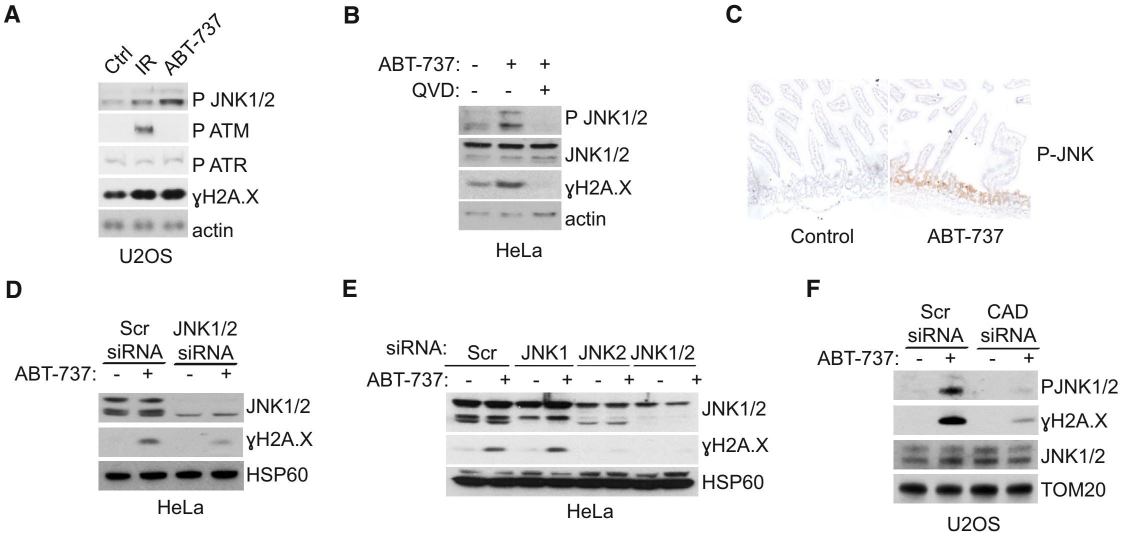

Figure 5. JNK Regulates the DNA Damage Response

(A) U2OS cells were treated for 3 hr with 10 μM ABT-737, and phospho JNK1/2, ATM, and ATR were assessed by western blot. Ionizing radiation (2 Gy) was used as a positive control.

(B) U2OS were treated with ABT-737 as in (A) in the presence or absence of Q-VD-OPh (10 μM) and immunoblotted for P-JNK1/2 and γH2A.X.

(C) Representative images of P-JNK1/2 immunohistochemical staining in small intestine of mice treated with ABT-737 (75 mg/kg) for 1 day (n = 3); untreated mice (n = 3) were used as control.

(D) HeLa cells were transiently transfected with siRNA for JNK1/2 and treated as in (A). Cell lysates were probed for total JNK1/2 and γH2A.X.

(E) As in (D), except that siRNA oligos targeting JNK1, JNK2, or both JNK1 and JNK2 together were used.

(F) U2OS cells were transfected with siRNA targeting CAD and treated with ABT-737. Cell lysates were probed for γH2A.X and P-JNK1/2.

See also Figure S5.

Cell survival following minority MOMP may be even more important in post-mitotic, non-renewable cells such as neurons. Recently, neurons have been found to rapidly degrade cytochrome *c* following MOMP in a manner dependent on the E3 ubiquitin-ligase PARC (Gama et al., 2014). Beyond promoting cell survival, degradation of cytochrome *c* also has the potential to limit minority MOMP-dependent DNA damage. Additionally, removal of individual, permeablized mitochondria, particularly under stressful conditions, may also affect the DNA-damaging effects of minority MOMP. Along these lines, different studies have found mitochondria are degraded in an autophagy-dependent manner following permeabilization (Colell et al., 2007; Xue et al., 2001).

A variety of non-apoptotic roles for mitochondrial dependent caspase activation have been described, particularly in neurons (Hyman and Yuan, 2012). These include caspase-dependent dendrite pruning and AMPA receptor internalization (Li et al., 2010; Simon et al., 2012). Minority MOMP could be a potential mechanism to establish sub-lethal caspase activity in such processes.

Besides the detrimental, oncogenic effects of minority MOMP, our findings also raise potentially important therapeutic possibilities. For example, manipulating minority MOMP to convert it to complete MOMP or enhancing downstream caspase activity may improve the cytotoxic effects of anti-cancer regimens. Conversely, inhibition of caspase activity downstream of minority MOMP could mitigate undesirable cytotoxicity and thereby reduce the systemic effects of chemotherapy.

EXPERIMENTAL PROCEDURES

Plasmids

CytoGFP plasmid was cloned as follows: the cDNA encoding 1XFKBP was obtained by PCR and inserted into the pEGFP-C1 plasmid via EcoRI and BamHI restriction sites. The MitoCherry plasmid was obtained by sub-cloning the N terminus sequence of AIF (N-terminal 1–90 amino acids) into pcDNA3 via HindIII and KpnI restriction sites, followed by mCherry fused with a glycine linker in between KpnI and NotI, the FRB domain was inserted via NotI/XhoI. Both CytoGFP and MitoCherry were transiently expressed and all treatments and confocal imaging were done 24 hr later. For generating Dox-inducible, stable expression in MelJuSo cells, C-terminally HA-tagged tBID-C in pLVX-Tight-Puro (Clontech) was used as previously described (Rooswinkel et al., 2014).

Microscopy

Both live-cell imaging and immunostaining analysis were carried out using a Nikon A1R confocal microscope (Nikon Instruments) with laser wavelengths of 405 nm, 488 nm, 561 nm, and 636 nm. Where live-cell imaging was carried out, the temperature was maintained at 37°C and CO_2 at 5% v/v in a humidified atmosphere. Images were acquired with a 60× NA 1.4 objective.

Micronuclei Assay

Cells were plated onto coverslips and treated for 36 hr with cytochalasin B in the culture media at a final concentration of 3 μg/ml. When the majority of cells were bi-nucleated, they were fixed in paraformaldehyde 4% in PBS for 10 min at room temperature, washed once in PBS, and mounted with DAPI-containing Vectashield. A minimum of 200 bi-nucleated cells was counted per condition.

Statistical Analysis

For comparison of multiple groups, two-way ANOVA was used while Student's t test was applied when comparing two groups. Analyses were performed using Prism 5.0 software (GraphPad).

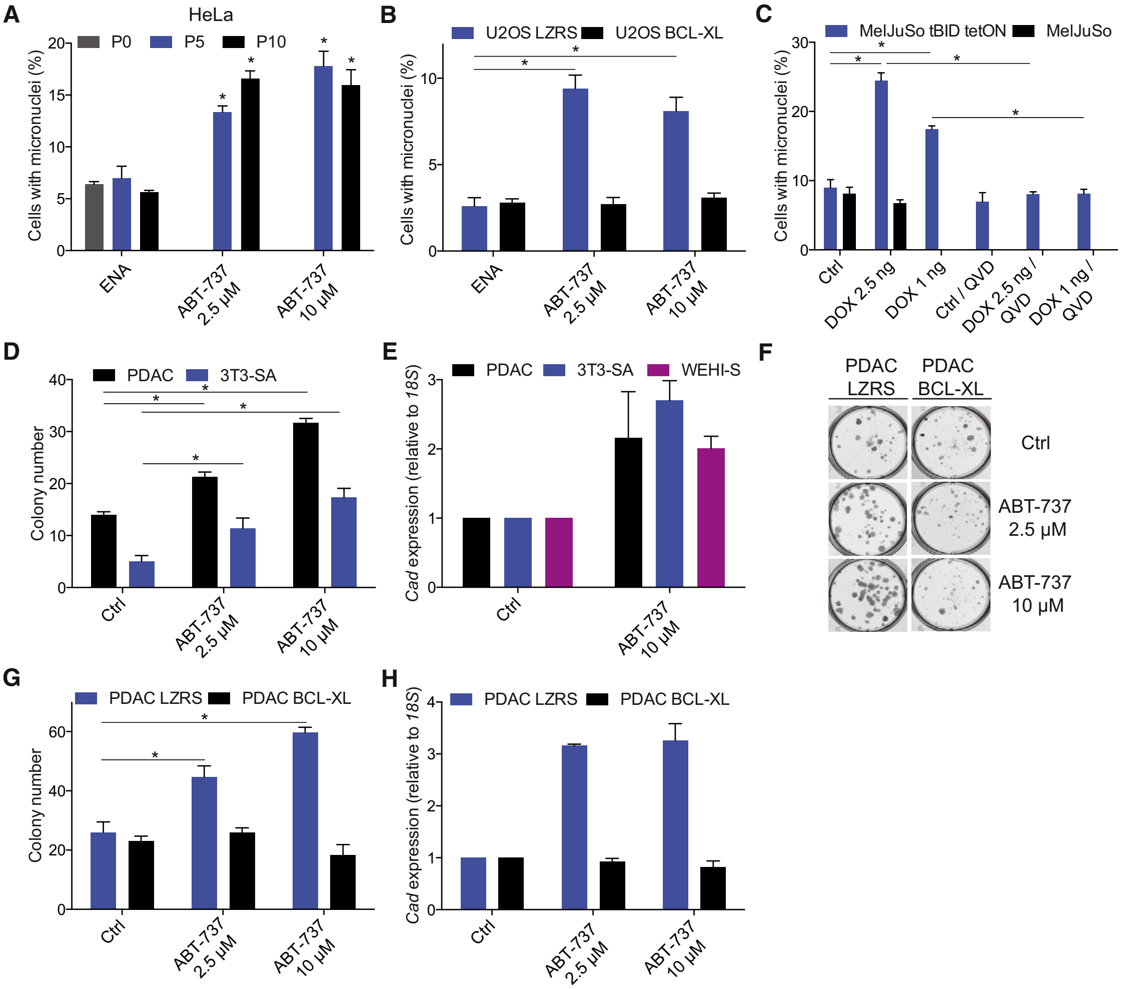

Figure 6. Minority MOMP Promotes Genomic Instability

(A) HeLa cells were treated daily with ABT-737 or enantiomer (10 µM, Ctrl) at the indicated concentrations for either 5 (P5) or 10 (P10) passages and then scored for micronuclei. Data represent mean ± SEM of three independent experiments.

(B) U2OS cells stably expressing BCL-xL (U2OS BCL-xL) or empty vector (U2OS LZRS) were treated as in (A) and assessed for micronuclei. Data represent mean ± SEM of three independent experiments.

(C) MelJuSo tetON or wild-type MelJuSo cells were treated daily for ten passages with the indicated concentration of doxycycline (DOX) in the absence or presence of Q-VD-OPh, and micronuclei were scored. Data represent mean ± SEM of three independent experiments.

(D) PDAC and 3T3-SA cells were treated daily for five passages with ABT-737 at the indicated concentrations, and clonogenic survival assay was performed in media containing PALA (100 µM). Data represent mean ± SEM of three independent experiments.

(E) Genomic DNA was extracted from PALA-resistant PDAC, 3T3-SA, and WEHI-S clones, and *Cad* gene levels were quantified by qPCR. Data represent the mean ± SD from triplicate samples from a representative experiment carried out twice independently.

(F) Representative images of PALA-resistant colonies from PDAC cells stably expressing BCL-xL (PDAC BCL-xL) or empty vector (PDAC LZRS). Cells were treated daily for five passages with ABT-737 at the indicated concentrations, and clonogenic survival assay was performed in media containing PALA (100 µM).

(G) Quantification of PALA-resistance clonogenic survival in PDAC BCL-xL versus PDAC LZRS cells. Data represent mean ± SEM of three independent experiments.

(H) *Cad* expression in PDAC BCL-xL and PDAC-LZRS PALA resistant colonies. Data represent the mean from a representative experiment carried out twice independently.

Where stated, *$p < 0.05$, compared versus control. See also Figure S6.

SUPPLEMENTAL INFORMATION

Supplemental Information includes Supplemental Experimental Procedures, seven figures, and two movies and can be found with this article online at http://dx.doi.org/10.1016/j.molcel.2015.01.018.

AUTHOR CONTRIBUTIONS

G.I., J.L., and S.W.G.T. conceived and designed the study. G.I. and J.L. performed the majority of experimental work. N.M. and D.J.M. carried out in vivo BH3-mimetic/immunohistochemistry analysis and bred mice for MEF

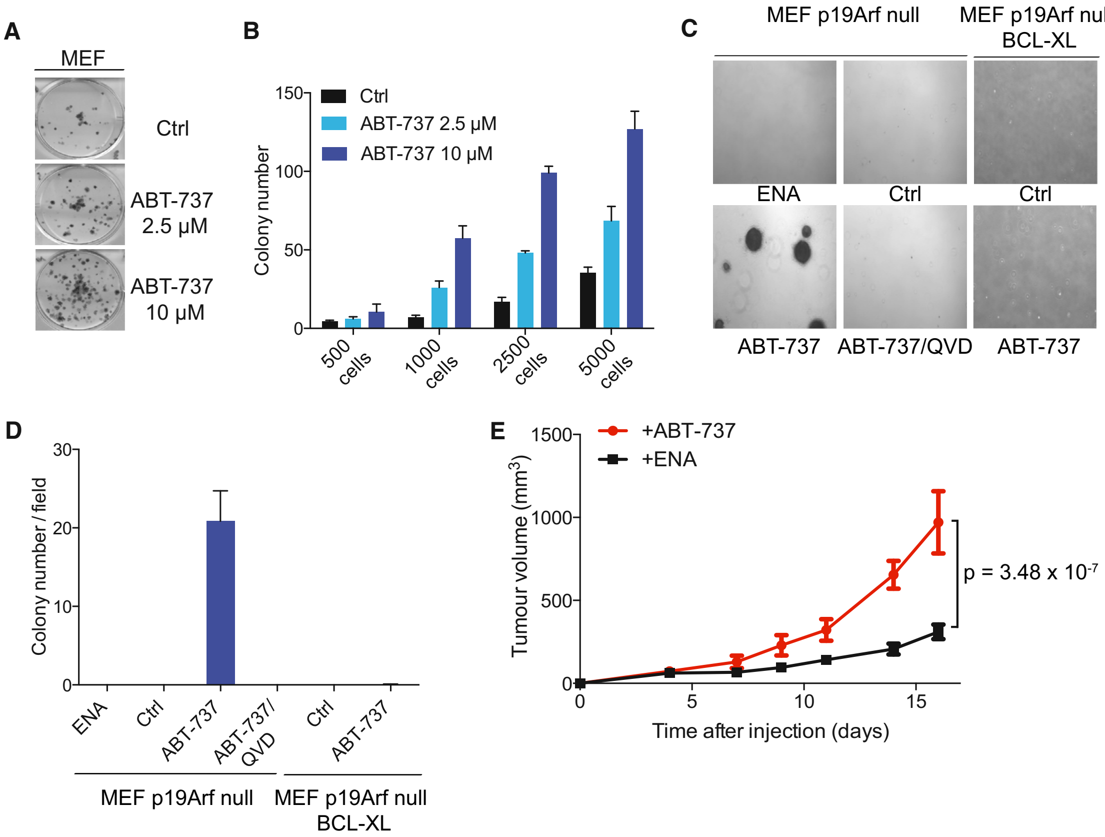

Figure 7. Minority MOMP Promotes Transformation and Tumorigenesis

(A and B) Primary mouse embryonic fibroblasts (MEF) were treated daily for seven passages with the indicated concentrations of ABT-737 and transduced with E1A- and KRAS-expressing retrovirus. Representative images are shown in (A), and quantitation of the number of transformed colonies is depicted in (B). Data represent mean ± SEM of triplicate samples for a representative experiment (out of three independent experiments).

(C) Primary $p19^{Arf}$ null MEFs or $p19^{Arf}$ null MEFs transduced with empty vector or BCL-xL-expressing retrovirus were treated for ten passages with ABT-737 (10 μM) in the presence or absence of Q-VD-OPh (10 μM), enantiomer (10 μM), or DMSO (Ctrl), and their anchorage-independent growth was assessed by soft agar assay. Representative images for each condition are shown.

(D) Quantification of triplicate samples (mean ± SEM) from one representative soft agar assay (out of three independent experiments).

(E) Primary $p19^{Arf}$ null MEF were treated with ABT-737 (10 μM) or enantiomer (ENA, 10 μM) for ten passages. CD1-*Nude* female mice were injected subcutaneously with treated MEF and tumor growth was measured over time. Data are plotted as mean with error bars representing 95% CI (n = 15 for both treatments). Applying Welch's t test to the area-under-the-curve data produces a p value of 3.48×10^{-7} for the tumor growth rates.

See also Figure S7.

generation. E.G. generated and characterized APAF-1 deficient cells. M.H. and J.S.R. performed biotin-VAD experiments and analysis of CRISPR/Cas-9-generated CAD-deficient cells. M.J.P. and L.B.-H. provided reagents and support for the inducible caspase-9 experiments. S.U.A. and A.J.C. provided technical advice/expertise for analyzing the DNA damage response. S.M.M., D.A., and K.B. carried out and analyzed the in vivo tumorigenesis experiments. B.v.d.K. and R.W.R. developed and characterized the inducible tBID cell line. M.E.D. and M.R. carried out computational modeling experiments. A.O. provided intellectual input and, together with G.I., J.L., and S.W.G.T., wrote the manuscript.

ACKNOWLEDGMENTS

This work was supported by funding from the Royal Society, an EU Marie Curie Career Integration Grant, BBSRC (grant BB/K008374/1 to S.W.G.T.), and funding from the European Union (FP7 APO-DECIDE) and Science Foundation Ireland (09-RFP-BIC2375 to M.R.). S.W.G.T. is a Royal Society University Research Fellow. G.I. was supported by an EMBO long-term postdoctoral fellowship (ALTF 55-2013). J.L. was supported, in part, by a fellowship from l'Association pour la recherché sur le cancer (ARC). E.G. was supported by a Cancer Research UK studentship. PALA compound was provided by the NIH Developmental Therapeutics Program. We thank AbbVie Pharmaceuticals and Drs. Feng Zhang, Didier Trono, Robert Weinberg, and Binghui Li for various reagents. We also thank BSU, David Strachan, Tom Gilbey, and Margaret O'Prey (Beatson Institute) for technical assistance; Matthew Neilson (Beatson Institute) for help with statistical analysis; and Catherine Winchester (Beatson Institute) for reviewing the manuscript.

Received: July 15, 2014
Revised: October 24, 2014
Accepted: January 8, 2015
Published: February 19, 2015

REFERENCES

Belshaw, P.J., Ho, S.N., Crabtree, G.R., and Schreiber, S.L. (1996). Controlling protein association and subcellular localization with a synthetic ligand that induces heterodimerization of proteins. Proc. Natl. Acad. Sci. USA *93*, 4604–4607.

Berardo, M.D., Elledge, R.M., de Moor, C., Clark, G.M., Osborne, C.K., and Allred, D.C. (1998). bcl-2 and apoptosis in lymph node positive breast carcinoma. Cancer *82*, 1296–1302.

Biswas, S., Shi, Q., Wernick, A., Aiello, A., and Zinkel, S.S. (2013). The loss of the BH3-only Bcl-2 family member Bid delays T-cell leukemogenesis in Atm-/- mice. Cell Death Differ. *20*, 869–877.

Colell, A., Ricci, J.E., Tait, S., Milasta, S., Maurer, U., Bouchier-Hayes, L., Fitzgerald, P., Guio-Carrion, A., Waterhouse, N.J., Li, C.W., et al. (2007). GAPDH and autophagy preserve survival after apoptotic cytochrome c release in the absence of caspase activation. Cell *129*, 983–997.

Delbridge, A.R., Valente, L.J., and Strasser, A. (2012). The role of the apoptotic machinery in tumor suppression. Cold Spring Harb. Perspect. Biol. *4*, pii: a008789.

Deshmukh, M., and Johnson, E.M., Jr. (1998). Evidence of a novel event during neuronal death: development of competence-to-die in response to cytoplasmic cytochrome c. Neuron *21*, 695–705.

Fenech, M. (2007). Cytokinesis-block micronucleus cytome assay. Nat. Protoc. *2*, 1084–1104.

Fresquet, V., Rieger, M., Carolis, C., García-Barchino, M.J., and Martinez-Climent, J.A. (2014). Acquired mutations in BCL2 family proteins conferring resistance to the BH3 mimetic ABT-199 in lymphoma. Blood *123*, 4111–4119.

Gama, V., Swahari, V., Schafer, J., Kole, A.J., Evans, A., Huang, Y., Cliffe, A., Golitz, B., Sciaky, N., Pei, X.H., et al. (2014). The E3 ligase PARC mediates the degradation of cytosolic cytochrome c to promote survival in neurons and cancer cells. Sci. Signal. *7*, ra67.

Goldstein, J.C., Waterhouse, N.J., Juin, P., Evan, G.I., and Green, D.R. (2000). The coordinate release of cytochrome c during apoptosis is rapid, complete and kinetically invariant. Nat. Cell Biol. *2*, 156–162.

Hars, E.S., Lyu, Y.L., Lin, C.P., and Liu, L.F. (2006). Role of apoptotic nuclease caspase-activated DNase in etoposide-induced treatment-related acute myelogenous leukemia. Cancer Res. *66*, 8975–8979.

Hyman, B.T., and Yuan, J. (2012). Apoptotic and non-apoptotic roles of caspases in neuronal physiology and pathophysiology. Nat. Rev. Neurosci. *13*, 395–406.

Jackson, S.P., and Bartek, J. (2009). The DNA-damage response in human biology and disease. Nature *461*, 1071–1078.

Labi, V., Erlacher, M., Krumschnabel, G., Manzl, C., Tzankov, A., Pinon, J., Egle, A., and Villunger, A. (2010). Apoptosis of leukocytes triggered by acute DNA damage promotes lymphoma formation. Genes Dev. *24*, 1602–1607.

Lartigue, L., Kushnareva, Y., Seong, Y., Lin, H., Faustin, B., and Newmeyer, D.D. (2009). Caspase-independent mitochondrial cell death results from loss of respiration, not cytotoxic protein release. Mol. Biol. Cell *20*, 4871–4884.

Li, Z., Jo, J., Jia, J.M., Lo, S.C., Whitcomb, D.J., Jiao, S., Cho, K., and Sheng, M. (2010). Caspase-3 activation via mitochondria is required for long-term depression and AMPA receptor internalization. Cell *141*, 859–871.

Lovric, M.M., and Hawkins, C.J. (2010). TRAIL treatment provokes mutations in surviving cells. Oncogene *29*, 5048–5060.

Lu, C., Zhu, F., Cho, Y.Y., Tang, F., Zykova, T., Ma, W.Y., Bode, A.M., and Dong, Z. (2006). Cell apoptosis: requirement of H2AX in DNA ladder formation, but not for the activation of caspase-3. Mol. Cell *23*, 121–132.

Martinou, I., Desagher, S., Eskes, R., Antonsson, B., André, E., Fakan, S., and Martinou, J.C. (1999). The release of cytochrome c from mitochondria during apoptosis of NGF-deprived sympathetic neurons is a reversible event. J. Cell Biol. *144*, 883–889.

Mathew, R., Karantza-Wadsworth, V., and White, E. (2009). Assessing metabolic stress and autophagy status in epithelial tumors. Methods Enzymol. *453*, 53–81.

Michalak, E.M., Vandenberg, C.J., Delbridge, A.R., Wu, L., Scott, C.L., Adams, J.M., and Strasser, A. (2010). Apoptosis-promoted tumorigenesis: gamma-irradiation-induced thymic lymphomagenesis requires Puma-driven leukocyte death. Genes Dev. *24*, 1608–1613.

Ni Chonghaile, T., and Letai, A. (2008). Mimicking the BH3 domain to kill cancer cells. Oncogene *27* (*Suppl 1*), S149–S157.

Oltersdorf, T., Elmore, S.W., Shoemaker, A.R., Armstrong, R.C., Augeri, D.J., Belli, B.A., Bruncko, M., Deckwerth, T.L., Dinges, J., Hajduk, P.J., et al. (2005). An inhibitor of Bcl-2 family proteins induces regression of solid tumours. Nature *435*, 677–681.

Orth, J.D., Loewer, A., Lahav, G., and Mitchison, T.J. (2012). Prolonged mitotic arrest triggers partial activation of apoptosis, resulting in DNA damage and p53 induction. Mol. Biol. Cell *23*, 567–576.

Otera, H., Ohsakaya, S., Nagaura, Z., Ishihara, N., and Mihara, K. (2005). Export of mitochondrial AIF in response to proapoptotic stimuli depends on processing at the intermembrane space. EMBO J. *24*, 1375–1386.

Reed, J.C. (1996). Mechanisms of Bcl-2 family protein function and dysfunction in health and disease. Behring Inst. Mitt. 72–100.

Rehm, M., Huber, H.J., Dussmann, H., and Prehn, J.H. (2006). Systems analysis of effector caspase activation and its control by X-linked inhibitor of apoptosis protein. EMBO J. *25*, 4338–4349.

Rooswinkel, R.W., van de Kooij, B., de Vries, E., Paauwe, M., Braster, R., Verheij, M., and Borst, J. (2014). Antiapoptotic potency of Bcl-2 proteins primarily relies on their stability, not binding selectivity. Blood *123*, 2806–2815.

Sakahira, H., Enari, M., and Nagata, S. (1998). Cleavage of CAD inhibitor in CAD activation and DNA degradation during apoptosis. Nature *391*, 96–99.

Shiloh, Y. (2003). ATM and related protein kinases: safeguarding genome integrity. Nat. Rev. Cancer *3*, 155–168.

Sim, S.P., and Liu, L.F. (2001). Nucleolytic cleavage of the mixed lineage leukemia breakpoint cluster region during apoptosis. J. Biol. Chem. *276*, 31590–31595.

Simon, D.J., Weimer, R.M., McLaughlin, T., Kallop, D., Stanger, K., Yang, J., O'Leary, D.D., Hannoush, R.N., and Tessier-Lavigne, M. (2012). A caspase cascade regulating developmental axon degeneration. The Journal of neuroscience *32*, 17540–17553.

Smith, G.C., d'Adda di Fagagna, F., Lakin, N.D., and Jackson, S.P. (1999). Cleavage and inactivation of ATM during apoptosis. Mol. Cell. Biol. *19*, 6076–6084.

Tait, S.W., and Green, D.R. (2010). Mitochondria and cell death: outer membrane permeabilization and beyond. Nat. Rev. Mol. Cell Biol. *11*, 621–632.

Tait, S.W., Parsons, M.J., Llambi, F., Bouchier-Hayes, L., Connell, S., Muñoz-Pinedo, C., and Green, D.R. (2010). Resistance to caspase-independent cell death requires persistence of intact mitochondria. Dev. Cell *18*, 802–813.

Tait, S.W., Ichim, G., and Green, D.R. (2014). Die another way—non-apoptotic mechanisms of cell death. J. Cell Sci. *127*, 2135–2144.

Tang, H.L., Tang, H.M., Mak, K.H., Hu, S., Wang, S.S., Wong, K.M., Wong, C.S., Wu, H.Y., Law, H.T., Liu, K., et al. (2012). Cell survival, DNA damage, and oncogenic transformation after a transient and reversible apoptotic response. Mol. Biol. Cell *23*, 2240–2252.

Van Damme, P., Martens, L., Van Damme, J., Hugelier, K., Staes, A., Vandekerckhove, J., and Gevaert, K. (2005). Caspase-specific and nonspecific in vivo protein processing during Fas-induced apoptosis. Nat. Methods *2*, 771–777.

Wright, K.M., Linhoff, M.W., Potts, P.R., and Deshmukh, M. (2004). Decreased apoptosome activity with neuronal differentiation sets the threshold for strict IAP regulation of apoptosis. J. Cell Biol. *167*, 303–313.

Xue, L., Fletcher, G.C., and Tolkovsky, A.M. (2001). Mitochondria are selectively eliminated from eukaryotic cells after blockade of caspases during apoptosis. Current biology: CB *11*, 361–365.

Zhang, J., Wang, X., Cui, W., Wang, W., Zhang, H., Liu, L., Zhang, Z., Li, Z., Ying, G., Zhang, N., and Li, B. (2013). Visualization of caspase-3-like activity in cells using a genetically encoded fluorescent biosensor activated by protein cleavage. Nat. Commun. *4*, 2157.

Molecular Cell

Supplemental Information

Limited Mitochondrial Permeabilization Causes DNA Damage and Genomic Instability in the Absence of Cell Death

Gabriel Ichim, Jonathan Lopez, Shafiq U. Ahmed, Nathiya Muthalagu, Evangelos Giampazolias, M. Eugenia Delgado, Martina Haller, Joel S. Riley, Susan M. Mason, Dimitris Athineos, Melissa J. Parsons, Bert van de Kooij, Lisa Bouchier-Hayes, Anthony J. Chalmers, Rogier W. Rooswinkel, Andrew Oberst, Karen Blyth, Markus Rehm, Daniel J. Murphy, and Stephen W.G. Tait

Figure S1

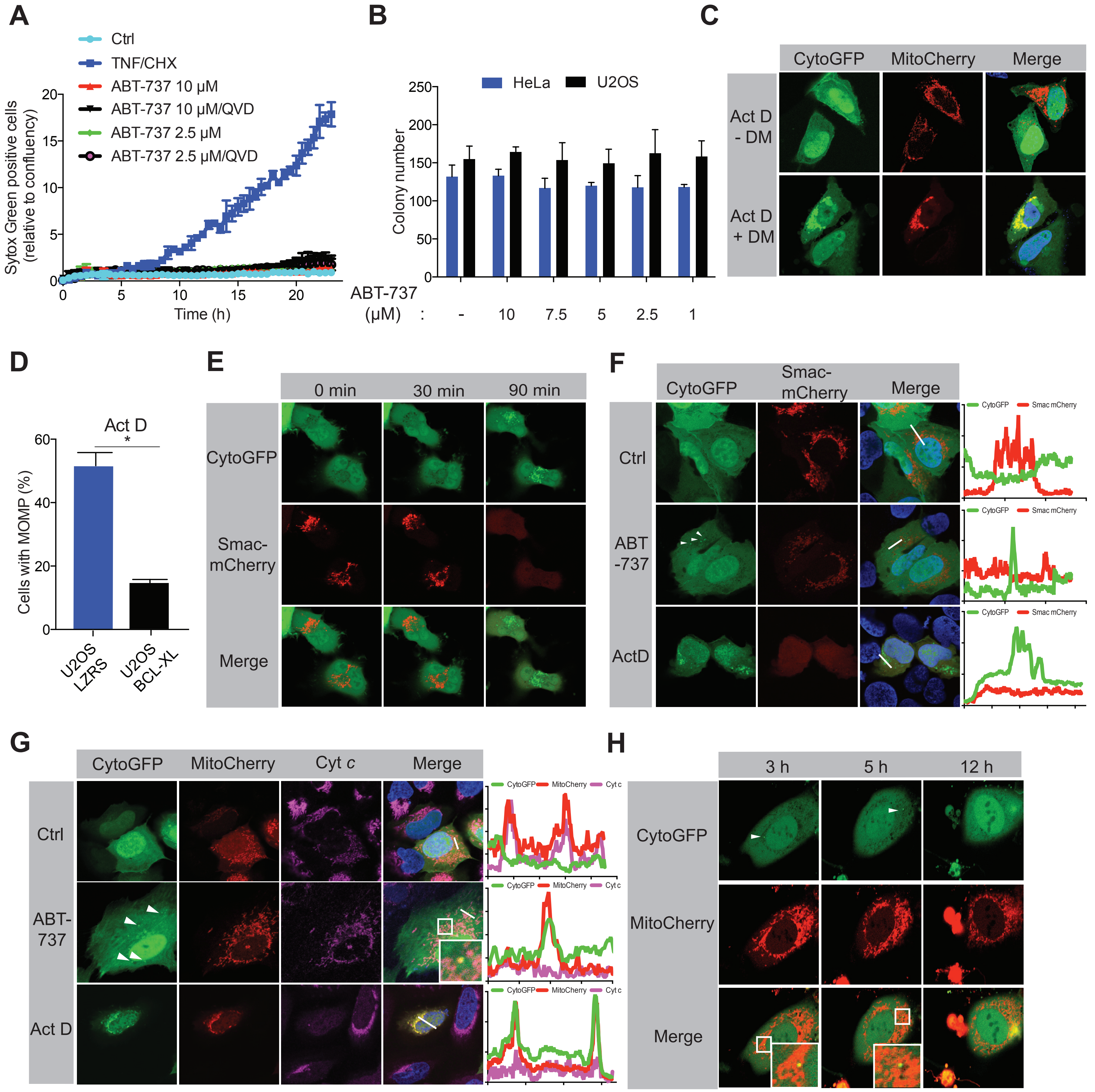
A
Ctrl
TNF/CHX
ABT-737 10 µM
ABT-737 10 µM/QVD
ABT-737 2.5 µM
ABT-737 2.5 µM/QVD
Sytox Green positive cells (relative to confluency)
Time (h)
B
HeLa
U2OS
Colony number
ABT-737 (µM) :
-
10
7.5
5
2.5
1
C
CytoGFP
MitoCherry
Merge
Act D - DM
Act D + DM
D
Act D
*
Cells with MOMP (%)
U2OS LZRS
U2OS BCL-XL
E
0 min
30 min
90 min
CytoGFP
Smac-mCherry
Merge
F
CytoGFP
Smac-mCherry
Merge
Ctrl
ABT -737
ActD
Smac mCherry
G
CytoGFP
MitoCherry
Cyt c
Merge
Ctrl
ABT-737
Act D
H
3 h
5 h
12 h
CytoGFP
MitoCherry
Merge

Figure S1 (related to Figure 1)

A. HeLa cells were treated with ABT-737 at the indicated concentrations over a 24-hour period and cell viability was continuously measured by Sytox Green exclusion in an Incucyte Imager. Graphs report the mean of a representative experiment repeated three times independently. As a positive control to induce apoptosis, tumour necrosis factor (TNF, 20ng/ml) cycloheximide (CHX, 1µg/ml) treatment was used. **B.** HeLa and U2OS cells were treated for 3 hours with varying concentrations of ABT-737 and quantified for long-term clonogenic survival. Graph represents the mean ± SEM of three independent experiments. **C**. U2OS cells expressing CytoGFP/MitoCherry were treated with Act D (1µM) for 3 hours in the presence or absence of heterodimeriser (DM) and imaged by confocal microscopy. **D**. U2OS cells stably expressing BCL-xL (U2OS BCL-xL) or empty vector (U2OS LZRS) together with CytoGFP/MitoCherry were treated with Act D (1µM for 12 hours) in the presence of heterodimeriser and the percentage of cells with complete MOMP was quantified. Data represent the mean ± SEM of three independent experiments. **E.** U2OS cells transiently expressing Smac-mCherry together with CytoGFP and MitoFRB (MitoCherry lacking mCherry) were treated with Act D (1µM) together with heterodimeriser and imaged by confocal microscopy for 90 minutes every 5 minutes. Representative images of the same two cells are shown over time. **F**. U2OS cells transiently expressing Smac-mCherry together with CytoGFP and MitoFRB in the presence of dimeriser were treated with ABT-737 for 3 hours. Act D was used as positive control to induce complete mitochondrial release of Smac-mCherry. Line scans represent variation in red and green fluorescence intensity along the denoted line. **G**. Same as in F, except U2OS cells transiently expressing CytoGFP and MitoCherry were immunostained for cytochrome *c*. **H.** U2OS cells transiently expressing CytoGFP and MitoCherry were treated for 3 hours prior to imaging with ABT-737 (5µM) in the presence of chemical dimeriser. Representative images over the time course are shown. Arrows and inset indicate mitochondria undergoing permeabilisation. Where stated,* represents $p<0.05$, compared versus control.

Figure S2

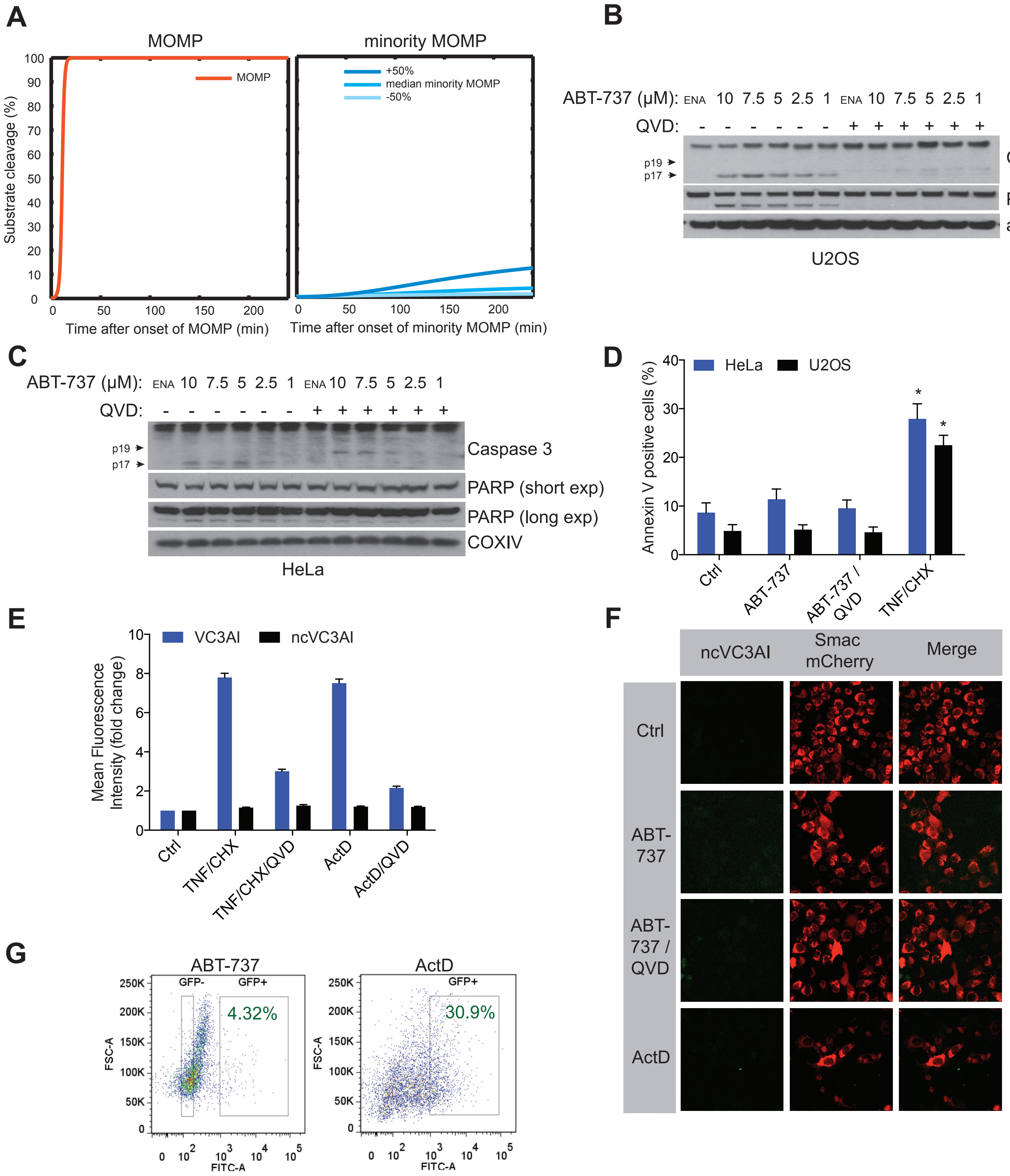
A
MOMP
minority MOMP
MOMP
+50%
median minority MOMP
-50%
Substrate cleavage (%)
Time after onset of MOMP (min)
Time after onset of minority MOMP (min)
B
ABT-737 (μM): ENA 10 7.5 5 2.5 1 ENA 10 7.5 5 2.5 1
QVD: - - - - - - + + + + + +
p19
p17
Caspase 3
PARP
actin
U2OS
C
ABT-737 (μM): ENA 10 7.5 5 2.5 1 ENA 10 7.5 5 2.5 1
QVD: - - - - - - + + + + + +
p19
p17
Caspase 3
PARP (short exp)
PARP (long exp)
COXIV
HeLa
D
HeLa
U2OS
Annexin V positive cells (%)
Ctrl
ABT-737
ABT-737 / QVD
TNF/CHX
E
VC3AI
ncVC3AI
Mean Fluorescence Intensity (fold change)
Ctrl
TNF/CHX
TNF/CHX/QVD
ActD
ActD/QVD
F
ncVC3AI
Smac mCherry
Merge
Ctrl
ABT-737
ABT-737 / QVD
ActD
G
ABT-737
ActD
GFP-
GFP+
4.32%
30.9%
FSC-A
FITC-A

Figure S2 (related to Figure 2)

A. The mathematical HeLa cell model of apoptosis execution was used to calculate the amounts of substrate cleavage by activated caspase-3 for scenarios of MOMP and minority MOMP. Model predictions demonstrate that minority MOMP triggers limited amounts of substrate cleavage, consistent with experimental validation data (see Figure 2D in the main manuscript). **B** and **C**. U2OS (B) or HeLa cells (C) were treated for 3 hours with varying doses of ABT-737 in the presence or absence of Q-VD-OPh (10μM) or enantiomer (10μM, ENA) and cell lysates were probed by Western blot for caspase-3 and PARP. **D**. HeLa and U2OS cells were treated for 3 hours with ABT-737 or TNF/CHX, stained for Annexin V and the percentage of dead cells was determined by flow-cytometry. Data represent mean ± standard error of the mean (SEM) of three independent experiments **E**. HeLa cells stably expressing the fluorescence caspase reporter VC3AI or the non-cleavable variant (ncVC3AI) were treated for 12 hours with TNF/CHX and Act D in the presence or absence of Q-VD-OPh and analysed by flow-cytometry for GFP mean fluorescence intensity. Data represent mean ± SEM of three independent experiments. **F**. HeLa cells stably expressing the non-cleavable caspase reporter (ncVC3AI) and transiently transfected with Smac-mCherry were treated with ABT-737 (10μM for 24h) and imaged by confocal microscopy; representative images are shown. **G**. Flow-cytometry dot plot profiles showing the GFP negative (ABT-737) and positive (ABT-737/Act D) cell populations sorted and assessed for clonogenic survival.

Figure S3

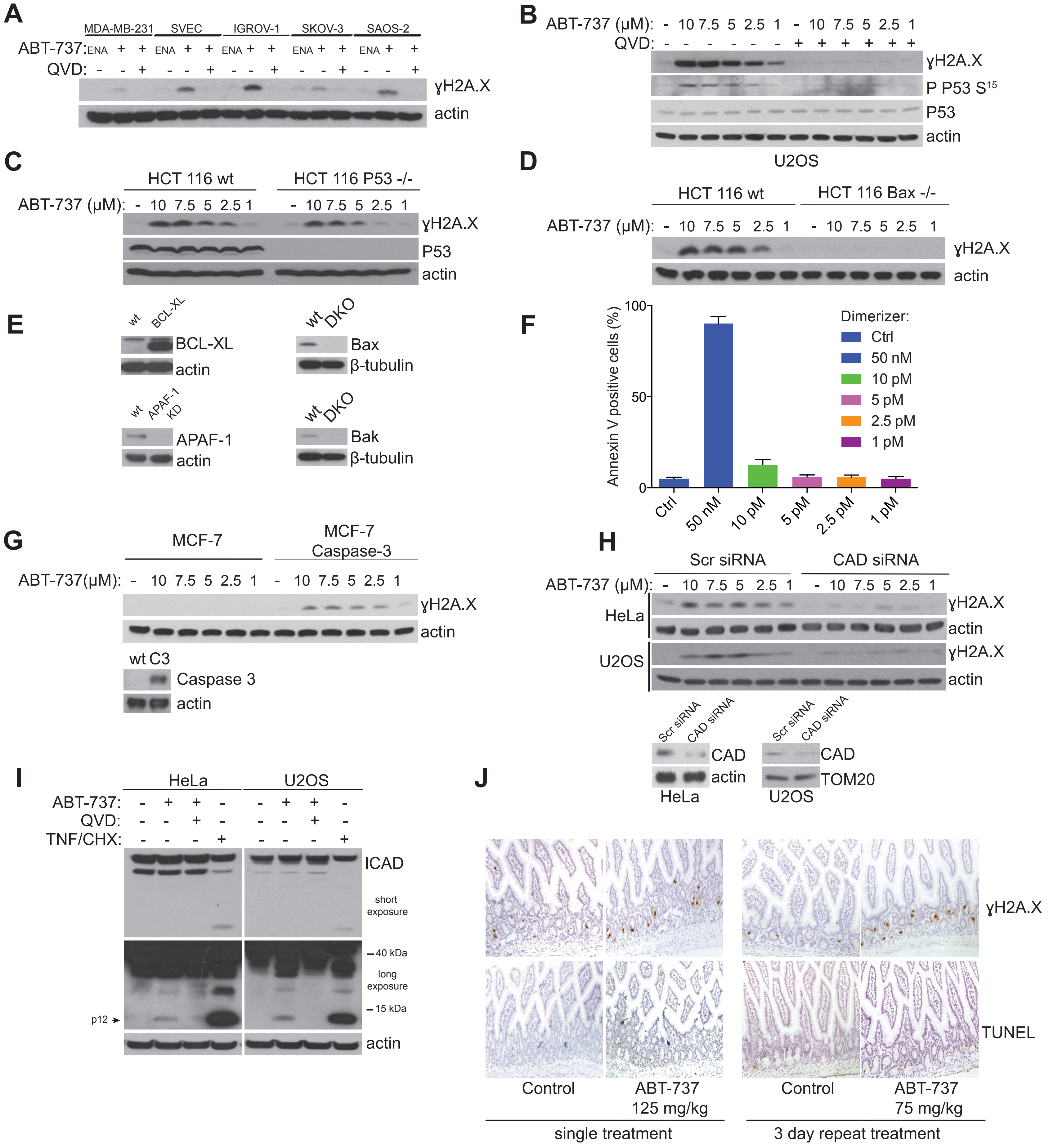
A
MDA-MB-231
SVEC
IGROV-1
SKOV-3
SAOS-2
ABT-737: ENA + + ENA + + ENA + + ENA + + ENA + +
QVD: - - + - - + - - + - - + - - +
ɣH2A.X
actin
B
ABT-737 (μM): - 10 7.5 5 2.5 1 - 10 7.5 5 2.5 1
QVD: - - - - - - + + + + + +
ɣH2A.X
P P53 S15
P53
actin
U2OS
C
HCT 116 wt
HCT 116 P53 -/-
ABT-737 (μM): - 10 7.5 5 2.5 1 - 10 7.5 5 2.5 1
ɣH2A.X
P53
actin
D
HCT 116 wt
HCT 116 Bax -/-
ABT-737 (μM): - 10 7.5 5 2.5 1 - 10 7.5 5 2.5 1
ɣH2A.X
actin
E
wt BCL-XL
BCL-XL
actin
wt DKO
Bax
β-tubulin
wt APAF-1 KD
APAF-1
actin
wt DKO
Bak
β-tubulin
F
Annexin V positive cells (%)
100
50
0
Dimerizer:
Ctrl
50 nM
10 pM
5 pM
2.5 pM
1 pM
Ctrl
50 nM
10 pM
5 pM
2.5 pM
1 pM
G
MCF-7
MCF-7 Caspase-3
ABT-737(μM): - 10 7.5 5 2.5 1 - 10 7.5 5 2.5 1
ɣH2A.X
actin
wt C3
Caspase 3
actin
H
Scr siRNA
CAD siRNA
ABT-737 (μM): - 10 7.5 5 2.5 1 - 10 7.5 5 2.5 1
HeLa
ɣH2A.X
actin
U2OS
ɣH2A.X
actin
Scr siRNA CAD siRNA
CAD
actin
HeLa
Scr siRNA CAD siRNA
CAD
TOM20
U2OS
I
HeLa
U2OS
ABT-737: - + + - - + + -
QVD: - - + - - - + -
TNF/CHX: - - - + - - - +
ICAD
short exposure
40 kDa
long exposure
15 kDa
p12
actin
J
ɣH2A.X
TUNEL
Control
ABT-737 125 mg/kg
Control
ABT-737 75 mg/kg
single treatment
3 day repeat treatment

Figure S3 (related to Figure 3)

A. The indicated cell lines were treated for 3 hours with ABT-737 (1μM) or enantiomer (1μM, ENA) and immunoblotted for γH2A.X. **B**. U2OS cells were treated with ABT-737 as in (A) and immunoblotted for phospho-S^{15} p53 and total p53. **C**. Wild type HCT-116 or HCT-116 p53 -/- cells were treated with ABT-737 as in (B) and cell lysates were probed for γH2A.X. **D**. Wild type HCT-116 and HCT-116 BAX -/- were treated and analysed as in (C). **E**. Expression analysis, Western blot for HeLa BCL-xL and HeLa APAF-1 KD, respectively. Right panel, Western blot validation of BAX and BAK knock-out MEF. **F**. A549 cells inducible for Caspase-9 dimerization/activation were treated for 3 hours with the indicated concentrations of homodimerizer (DM), stained for Annexin V and analysed by flow cytometry for cell death. Data represent mean ± standard error of the mean (SEM) of three independent experiments. **G**. Wild type MCF-7 or MCF-7 stably expressing Caspase-3 were treated with ABT-737 as in (C) and cell lysates were probed for γH2A.X. **H**. HeLa and U2OS cells were transfected with CAD siRNA and then treated with ABT-737 as in (B). The lower panels show the efficacy of CAD knock-down determined by Western blot. **I**. U2OS and HeLa cells were treated with ABT-737 (10μM for 3 hours) and cell lysates were blotted for ICAD (antibody recognizing both full length and cleaved ICAD). TNF/CHX treatment was used as positive control for ICAD cleavage. **J** Representative images of γH2A.X and TUNEL staining in small intestine from mice treated with ABT 737 (125 mg/kg for 1 day or 75 mg/kg for three consecutive days). Control mice were administered with vehicle.

Figure S4

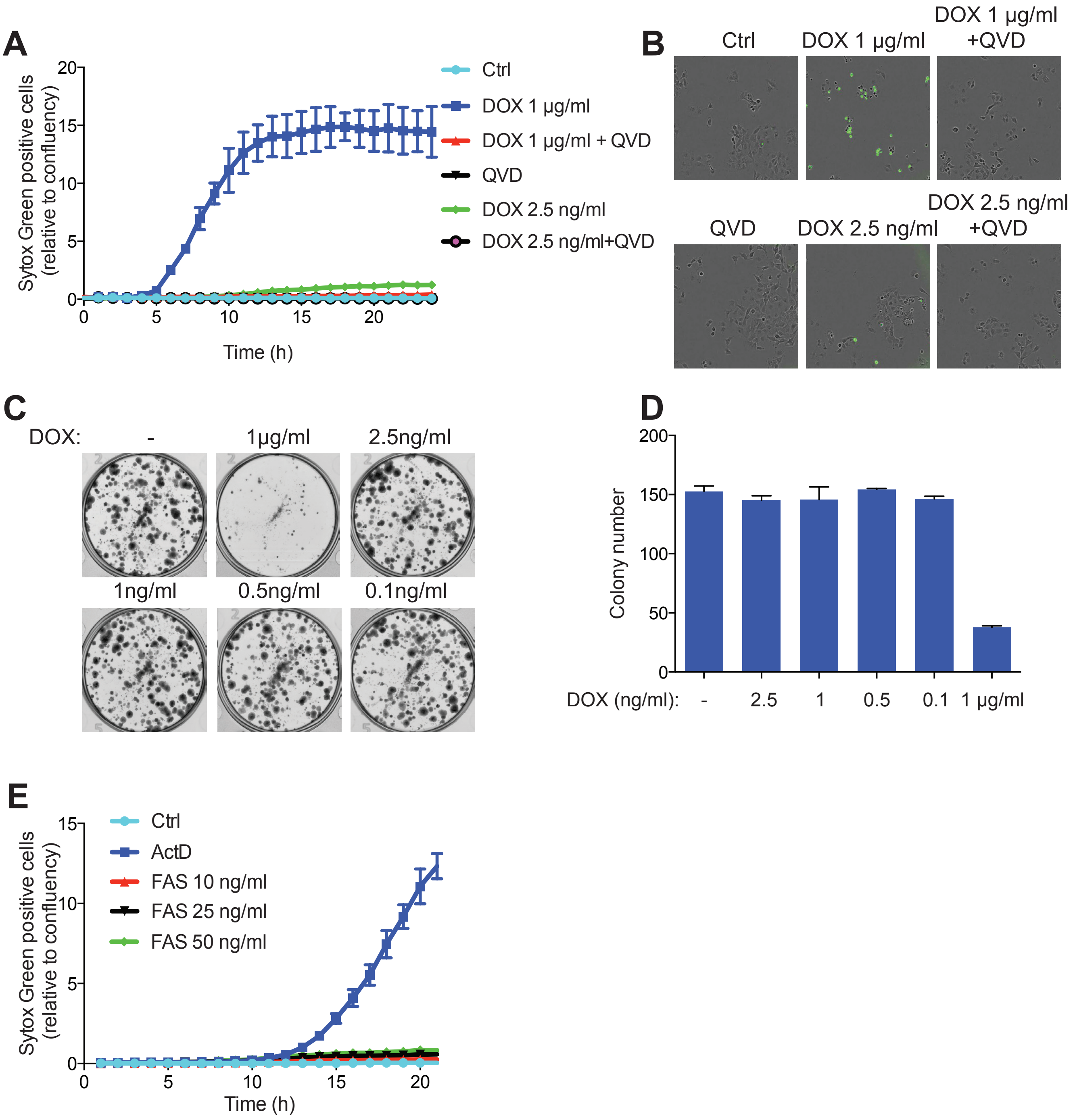

A
Sytox Green positive cells (relative to confluency)
Time (h)
Ctrl
DOX 1 µg/ml
DOX 1 µg/ml + QVD
QVD
DOX 2.5 ng/ml
DOX 2.5 ng/ml+QVD
B
Ctrl
DOX 1 µg/ml
DOX 1 µg/ml +QVD
QVD
DOX 2.5 ng/ml
DOX 2.5 ng/ml +QVD
C
DOX:
-
1µg/ml
2.5ng/ml
1ng/ml
0.5ng/ml
0.1ng/ml
D
Colony number
DOX (ng/ml):
-
2.5
1
0.5
0.1
1 µg/ml
E
Sytox Green positive cells (relative to confluency)
Time (h)
Ctrl
ActD
FAS 10 ng/ml
FAS 25 ng/ml
FAS 50 ng/ml

Figure S4 (related to Figure 4)

A. MelJuSo tBid tetON cells were treated for 24 hours with the indicated concentrations of doxycycline (DOX) and cell viability was measured by Sytox Green exclusion in an Incucyte Imager. One representative experiment out of three independent repeats is shown. **B**. Images of MelJuSo tBid tetON cells treated with DOX as in (A). Images were taken at 12 hour after DOX treatment, Sytox Green positive cells are non-viable. **C** and **D**. MelJuSo tBid tetON cells were treated for 3 hours with the indicated concentrations of DOX followed by long-term clonogenic survival assay. Representative images are shown in (C) and quantitation is depicted in (D). Data represent mean ± SEM from three independent experiments. **E**. U2OS cells were treated with FAS ligand and CHX at the indicated concentrations and cell viability was measured by Sytox Green exclusion. One representative experiment is shown (repeated three times independently).

Figure S5

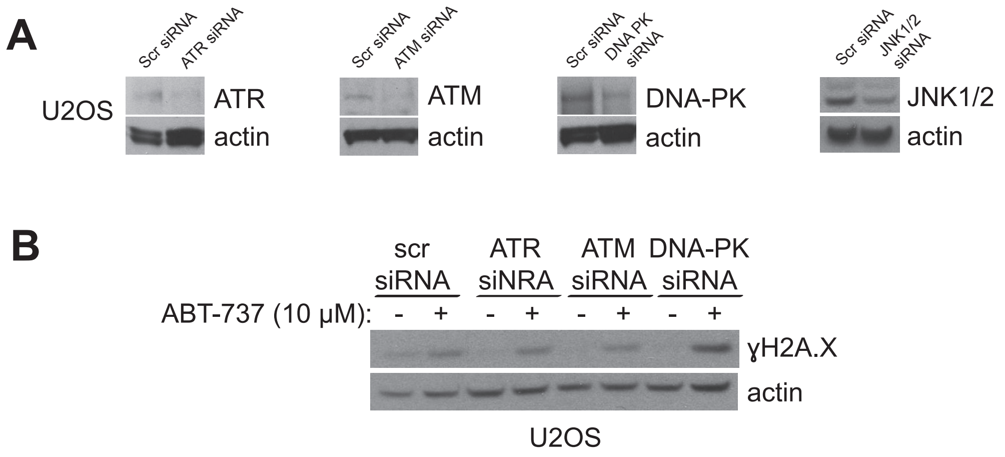
A
Scr siRNA
ATR siRNA
U2OS
ATR
actin
Scr siRNA
ATM siRNA
ATM
actin
Scr siRNA
DNA PK siRNA
DNA-PK
actin
Scr siRNA
JNK1/2 siRNA
JNK1/2
actin
B
scr siRNA
ATR siNRA
ATM siRNA
DNA-PK siRNA
ABT-737 (10 μM):
- + - + - + - +
ɣH2A.X
actin
U2OS

Figure S5 (related to Figure 5)

A. Western blot analysis of ATM, ATR, DNA PK and JNK1/2 expression in U2OS cells following siRNA transfection. **B**. U2OS cells expressing siRNA targeting ATR, ATM or DNA-PK were treated with ABT-737 (10μM for 3 hours) and cell lysates were Western blotted for γH2A.X.

Figure S6

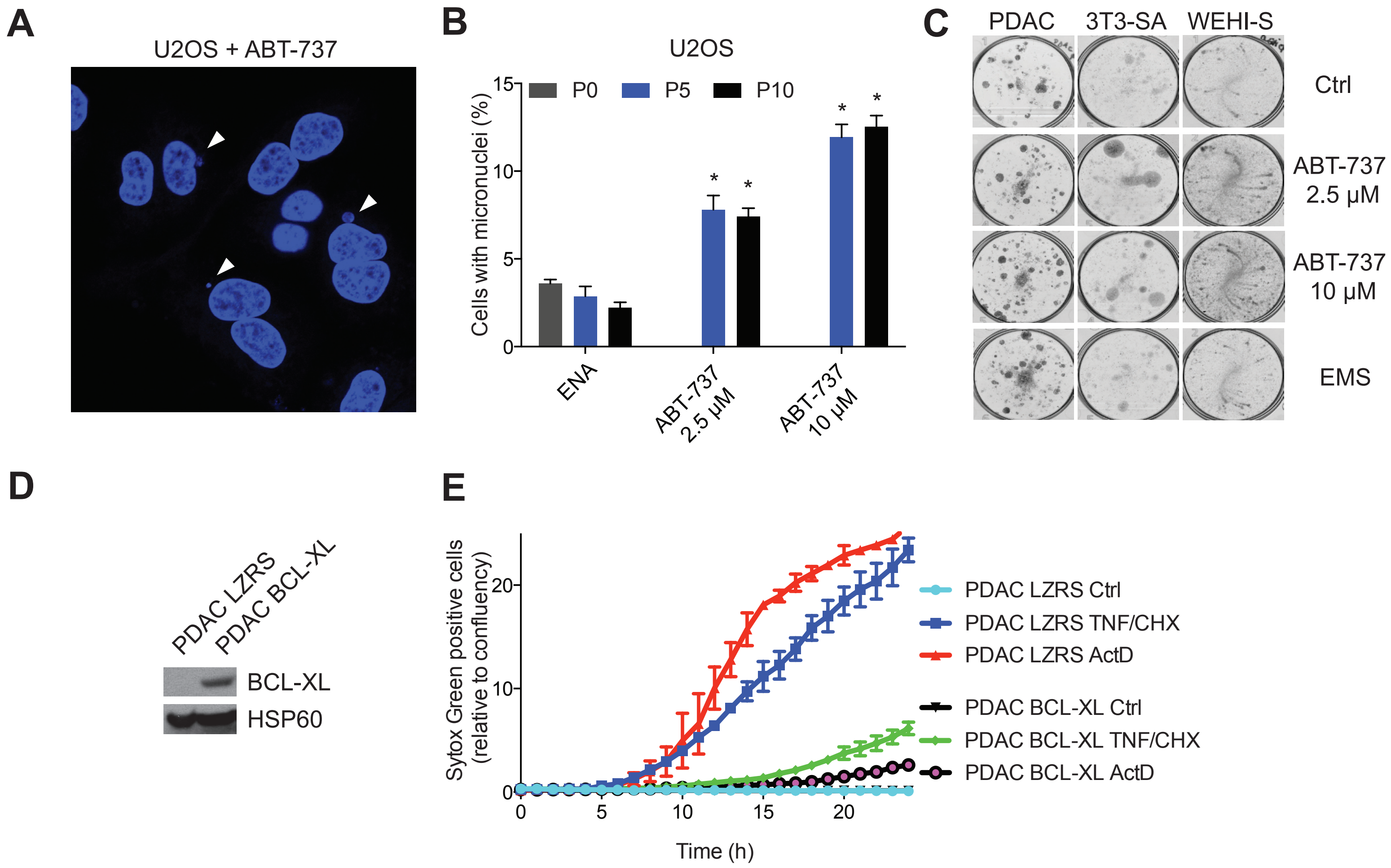

A
U2OS + ABT-737
B
U2OS
P0
P5
P10
Cells with micronuclei (%)
15
10
5
0
*
ENA
ABT-737
2.5 µM
ABT-737
10 µM
C
PDAC
3T3-SA
WEHI-S
Ctrl
ABT-737
2.5 µM
ABT-737
10 µM
EMS
D
PDAC LZRS
PDAC BCL-XL
BCL-XL
HSP60
E
Sytox Green positive cells
(relative to confluency)
20
10
0
5
15
Time (h)
PDAC LZRS Ctrl
PDAC LZRS TNF/CHX
PDAC LZRS ActD
PDAC BCL-XL Ctrl
PDAC BCL-XL TNF/CHX
PDAC BCL-XL ActD

Figure S6 (related to Figure 6)

A. Representative image of U2OS cells treated daily for 10 passages with ABT-737 (10μM). Nuclei were stained using DAPI. **B**. U2OS cells were treated daily with ABT-737 or enantiomer (10μM, Ctrl) at the indicated concentrations for either 5 (P5) or 10 (P10) passages and then scored for micronuclei. Data represent mean ± SEM of three independent experiments. **C**. Representative images of PALA-resistant colonies for PDAC, 3T3-SA and WEHI-S. **D**. Analysis of BCL-xL expression by Western blot in stably overexpressing PDAC cells. **E**. Incucyte viability analysis of PDAC cells stably expressing BCL-xL (PDAC BCL-xL) or empty vector (PDAC LZRS) and treated for 24 hours with the indicated apoptotic stimuli.

Figure S7

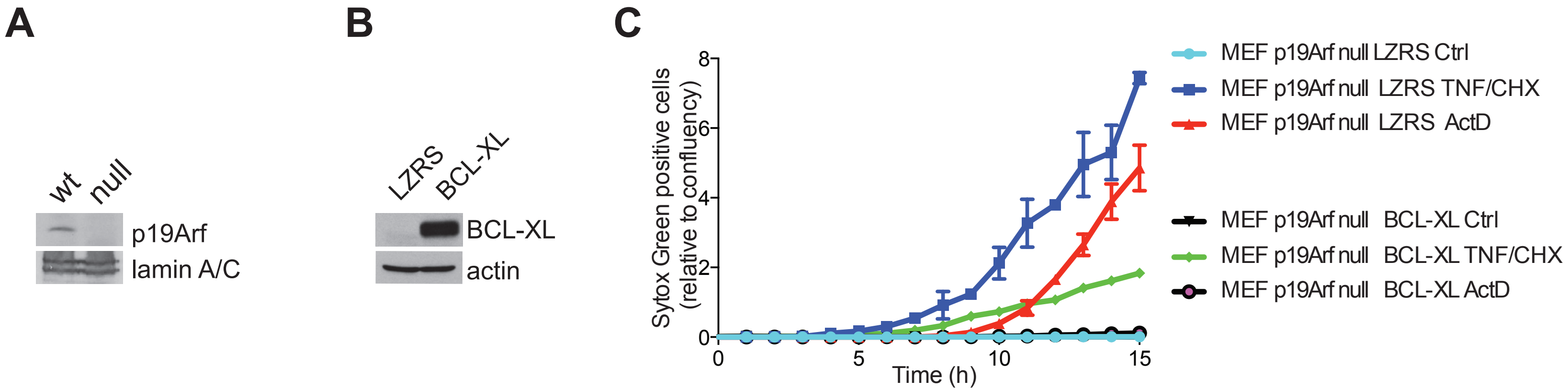
A
wt
null
p19Arf
lamin A/C
B
LZRS
BCL-XL
BCL-XL
actin
C
Sytox Green positive cells
(relative to confluency)
8
6
4
2
0
0
5
10
15
Time (h)
MEF p19Arf null LZRS Ctrl
MEF p19Arf null LZRS TNF/CHX
MEF p19Arf null LZRS ActD
MEF p19Arf null BCL-XL Ctrl
MEF p19Arf null BCL-XL TNF/CHX
MEF p19Arf null BCL-XL ActD

Figure S7 (related to Figure 7)

A. Western blot validation of $p19^{Arf}$ deficiency in $p19^{Arf}$ null MEFs. **B**. Western blot analysis of BCL-xL expression in vector and BCL-xL transduced $p19^{Arf}$ null MEF. **C**. Incucyte, Sytox Green exclusion-based viability analysis of $p19^{Arf}$ null MEF stably expressing BCL-xL and treated for 15 hours with the indicated apoptotic stimuli.

Supplemental Experimental Procedures

Cell lines and reagents

All cell lines were maintained in DMEM high glucose medium supplemented with 10% FCS, 2mM glutamine, 1mM sodium pyruvate, 50μM β-mercaptoethanol and penicillin/streptomycin. ABT-737 and Q-VD-OPh were obtained from ApexBio, the ABT-737 enantiomer was obtained from Abbvie Pharmaceuticals. PALA compound was obtained through the NCI Developmental Therapeutics Program.

Microscopy

For live cell imaging, cells were plated at 2×10^5 cells in glass bottom 35 mm microwell dishes (MatTek Corporation), transfected next day as indicated in the respective figure legend and imaged 24 hour later. Immunofluorescence staining for γH2A.X and cytochrome *c* was as follows: U2OS cells transiently expressing CytoGFP and MitoCherry, were fixed in 4% PFA/PBS for 10 minutes and permeabilised in 0.2 % Triton/PBS for 15 minutes followed by 1 hour blocking in 2% BSA/PBS. The primary antibody for phospho H2A.X (Cell Signalling, 2577, 1/100 in PBS) and cytochrome *c* (BD Biosciences, 556432, 1/300 in PBS) was incubated overnight. AlexaFluor 647 goat anti-rabbit was used as secondary antibody (Life Technologies, A21245, 1/300 in PBS). For visualizing minority MOMP, U2OS, HeLa, MelJuso tBID tetON or PDAC cells were transiently transfected in 12-well plates using GeneJuice. 24 hours later, cells were treated as described in the figures legend together with A/C heterodomerizer (50nM, Clontech, 635057). Cells were then fixed with PFA 4% in PBS for 10 minutes at room temperature and mounted using Vectashield mounting medium with DAPI (Vector Laboratories, H-1200). Cells displaying minority MOMP were then scored under the confocal microscope. Images were analyzed with ImageJ 1.47i.

E1A/Ras transformation and soft-agar assays

Primary MEF cells were isolated from E14 embryos (C57BL/6 strain) and cultured with ABT-737 for 5 passages. Cells were then plated in 6-well plates, in triplicate at 500, 1000, 2500 or 5000 cells/well. The following day cells were

infected with retroviruses encoding E1A and *KRas*. 24 hours later fresh media with 0.5 µg/ml puromycin (for *KRas*) and 40µg/ml hygromycin B (for E1A) was added and 7 days later the colonies were stained using methylene blue (1 % w/v in 50/50 methanol/water v/v). For soft-agar assays, primary p19^{ARF} null MEF were generated from E14 embryos derived from *Arf*-null mice *Cdkn2atm1(GFP)Cjs* (*ARF GFP*) mice, wherein GFP is inserted into exon 1 β of the *Cdkn2a* locus, abrogating p19^{ARF} expression (Zindy et al., 2003). Cells were treated with enantiomer or ABT-737 (10μM) in the presence or absence of caspase-inhibitor Q-VD-OPh for 10 passages. Alternatively, cells were transduced with empty LZRS-zeo retrovirus or retrovirus encoding BCL-xL and selected with Zeocin (200μg/ml) prior to treatment. Soft agar assay was performed as follows: a 1% base agarose solution (Low gelling temperature agarose, Sigma, A9414-25G) was poured into 6-well plates and allowed to solidify. Meanwhile, 7500 cells were suspended in 0.6% agarose solution in order to reach a final concentration of 0.3% and then laid on top of the base agarose. When the agarose solution solidified the dish was covered in complete DMEM media and colonies were counted 14 days later. Representative images for the soft agarose colonies were taken using Qcapture Pro7 software (QImaging).

Immunohistochemistry

All immunohistochemistry (IHC) staining was performed on Zinc-formalin fixed, paraffin-embedded sections (4µm thick). TUNEL staining was performed using the ApopTag peroxidase labelling kit according to the manufacturer's instructions (Millipore, S7100). An additional blocking step (1% BSA for 1hr at RT) was incorporated prior to addition of peroxidase-conjugated anti-digoxigenin. Anti-phospho H2A.X (Cell Signalling, 2577) and anti-pJNK (Thr183/Tyr185) (Abcam, ab18680) were used at 1:50 dilution after antigen retrieval with sodium citrate buffer (pH 6.0). All tissues were counterstained in Gil 1 haematoxylin followed by blueing solution.

Western blotting

Cell lysates were prepared using NP-40 lysis buffer (1% NP-40, 1mM EDTA, 150mM NaCl, 50mM Tris pH7.4, 1mM PMSF, Complete Protease Inhibitors [Roche]). Protein content was determined by Bio-Rad assay, proteins were separated by SDS-PAGE and blotted onto nitrocellulose. Membranes were probed with antibodies at 1/1000 dilution unless otherwise stated: anti-actin (MP Biomedicals, 8691001, 1/10000), anti-Tom20 (Santa Cruz, sc-11415), anti-BCL-xL (Abcam, ab32370), anti-cytochrome *c* (BD, 556433), anti-COXIV (Cell Signalling, 4850), anti-caspase-3 (Cell Signalling, 9662), anti-PARP (Cell Signalling, 9532, 1/1000), anti-γH2A.X (Cell Signalling, 9718S), anti-HSP60 (Santa Cruz, sc-13115), anti-BAK (Cell Signalling, 12105), anti-BAX (Santa Cruz, sc-493), anti-ICAD (Abcam, ab108521), anti-CAD (Santa Cruz, sc-374067), anti-APAF1 (Cell Signalling, 8723), anti-DNA-PK (Abcam, ab1832), anti-ATM (Abcam, ab2618), anti-P-ATM S1981 (Novus, 100-307), anti-ATR (Santa Cruz, sc-1887, 1/250), anti-P-ATR S428 (Cell Signalling, 2853), anti-JNK (Cell Signalling, 9252S), anti-P-JNK T183/Y185 (Cell Signalling, 4668S, 1/1000), anti-p53 (BD Biosciences, 554293), anti-P-S10 p53 (Cell Signalling, 9286), anti caspase-7 (Cell Signalling, 9492), anti caspase-9 (Cell Signalling, 9502S), anti caspase-8 (Enzo, ALX-804-429-C050) antibodies followed by incubation with the appropriate HRP conjugated secondary antibody and detection of immunoreactive proteins by ECL.

Comet assay

The alkaline Comet assay was used as described in the manufacturer's manual (Trevigen, 4250-050-K). The comet tail length was measured in ImageJ.

zVAD-biotin pull-downs

For caspase-3 and-7 pull-down, HeLa cells were treated with the indicated concentrations of ABT-737, Act D and TNF/CHX for 3 hours and then zVAD-biotin (Santa Cruz, sc-311290) was incubated with the cells for another 3 hours at 50μM final concentration. Cell were lysed in RIPA buffer (150 mM NaCl, 0.5% sodium deoxycholate, 0.1% SDS, and 50 mM Tris, pH 8.0) and

250 µg total protein (determined by Bio-Rad assay) were incubated overnight at 4°C with neutravidin agarose resin (Thermo Scientific, 29202). The following day, the resin was washed 3 times in RIPA buffer and once in PBS and then denaturated at 95°C with 30 µl sample buffer. For the caspase-9 and caspase-8 pulldowns the procedure was identical except zVAD-biotin was incubated with the cells for 1 hour prior to and maintained throughout the treatment period.

Generation of CAD deficient cells by CRISPR/Cas9 genome editing

Oligos targeting the following human CAD sequence: CAGCCCGAGGAAGTTCGGCG were cloned into LentiCRISPRv1 plasmid (obtained from Addgene, 49535) as previously described (Shalem et al., 2014). Following transduction, CAD-deficient HeLa and U20S cells were selected with puromycin for 2 weeks prior to analysis.

PALA assay

The PALA LD_{50} was determined for the three murine cell lines. Cells were cultured in nucleoside-free α-MEM containing Pen/Strep and 10% dialyzed FBS. Briefly, cells were plated in 96-well plate at a density of 500 and 1000 cells per well. PALA treatment was added at concentrations ranging from 0 to 100 µM. Three days later, MMT assay was performed to determine viability. Briefly, 10 % (v/v) MTT (5 mg/ml in PBS) was added to the media and incubated for 2 hours at 37°C. DMSO was added to solubilize the MTT crystals and the absorbance was read at 560 nm. For PALA resistance assay, cells were cultured in triplicates in 6-well plates at a density of 2500 cells/well. PALA was added at 3xLD_{50} and the treatment was carried out until colonies were visible. At the end of the experiment, colonies were stained using methylene blue (1 % in 50/50 methanol/water v/v). qPCR was performed on PALA-resistant colonies to assess *Cad* genomic amplification as previously described (Mathew et al., 2009). PCR fragments were amplified 3 min at 95°C, followed by 40 cycles of 20 sec at 95°C, 30 sec at 57°C, 30 sec at 72°C and final 5min at 72°C. The following primers were used: mCAD For AAGCTCAGATCCTAGTGCTAACG, mCAD Rev –

CCGTAGTTGCCGATGAGAGG, m18S For – ATGGTAGTCGCCGTGCCTAC and m18S Rev – CCGGAATCGAACCCTGATT.

Digitonin fractionation

Cells were washed once in PBS and re-suspended in a solution containing 70 mM Tris and 250 mM sucrose, pH 7.0. Digitonin (Sigma-Aldrich, D5628) was added to 100 µg/ml on ice for 1 minute and the cells were immediately centrifuged at 3000 rpm for 3 minutes. The supernatant was collected as cytosol and the pellet was solubilised in RIPA buffer (150 mM NaCl, 0.5% sodium deoxycholate, 0.1% SDS, and 50 mM Tris, pH 8.0) to obtain the whole cell lysate fraction.

Retroviral and lentiviral transduction

Phoenix Ampho (for human retrovirus), Phoenix Eco (for mouse retrovirus) or 293T cells (for lentivirus) (2 x 10^6 in a 10 cm dish) were transfected with LZRS zeo, LZRS FLAG-BCL-xL, pCDH-puro-CMV-VC3AI, pCDH-puro-CMV-ncVC3AI, pWZLH.E1A or pBabePuro.H-ras using Lipofectamine 2000 (Invitrogen) according to the manufacturer's instructions. The helper plasmids for lentivirus production were pVSVg (Addgene 8454)(Stewart et al., 2003) and psPAX2 (Addgene 12260). Two days later virus-containing supernatant was harvested, filtered and used infect target cells in the presence of 1μg/ml polybrene. Two days post-infection, stably expressing cells were selected by growth in 200ug/ml Zeocin (Invitrogen) or 1ug/ml puromycin, respectively. MelJuSo cells with Dox-inducible expression of tBid proteins were created by sequential lentiviral transduction with pLVX-Tet-On Advanced (Clontech) and pLVX-tight encoding tBid as previously described (Rooswinkel et al., 2014).

siRNA and plasmid transfection

Cells were transfected with 25 nM non-targeting control or the individual RNAi duplexes (Thermo Scientific) using Lipofectamine 2000 (Invitrogen) according to the manufacturer's protocol. Alternatively, for plasmid transfection GeneJuice transfectant (Merck Millipore) was used according to the manufacturer's instructions. The following siRNA oligos were used: hCAD

(Dharmacon, D-004425-01), hJNK1 (Dharmacon, M-003514-04), hJNK2 (Dharmacon, M-003505-02), hATM (Dharmacon, M-003201-04), hATR (Dharmacon, M-003202-05), hDNA-PK (Dharmacon, M-005030-01).

Cell viability assays

Cell viability was determined using an Incucyte FLR imaging system (Essen Bioscience). Cells were plated in medium containing 30nM Sytox Green (Invitrogen S7020). Cells were treated as described, imaged every 60 minutes over a period of 24 hours and analysed using Incucyte image analysis software (Essen Bioscience). The number of SytoxGreen positive cells was normalized to the confluency factor of the respective well. Alternatively, cell viability was analysed by flow cytometry using a FACS calibur (BD Biosciences). For this purpose, cells were stained with Alexa Fluor 647-Annexin V (BioLegend) according to the manufacturer's protocols. Analysis was performed using CellQuest Pro software (BD Biosciences).

Caspase-reporter based cell sorting

HeLa cells stably expressing the VC3AI caspase-3 reporter were treated for 24 hours with ABT-737 (10 μM) or 12 hours with 0.5 μM actinomycin D. Following treatment, equal numbers of GFP positive (ABT-737 or actinomycin D treated) or negative (ABT-737 treated sample) were sorted into 6-well plates using a FACSAria Fusion cell sorter (BD Biosciences). Clonogenic survival was quantified after 2 weeks using methylene blue (1 % w/v in 50/50 methanol/water v/v).

Mathematical modelling

The mathematical model of apoptosis execution has been described previously (Rehm et al., 2006). In brief, it comprises 19 reaction partners and 53 reactions, implemented as ordinary differential equations on the basis of mass action kinetics. The model has been parameterised and validated to accurately simulate the non-linear signalling from MOMP to caspase-3 activation and apoptosis execution in HeLa cervical cancer cells. For the analysis of the consequences of minority MOMP, here the model was adapted by modulating the strength of model input functions for mitochondrial Smac

release and cytochrome *c* driven caspase-9 activation, according to quantitative measurements of minority MOMP (Figure 2A). Calculations of the total amount of processed caspase-3 and the resulting cleavage of caspase-3 substrates served as model outputs. Simulations were conducted using MatLab (The Mathworks, UK).

In vivo experiments

All procedures were approved by Animal Welfare and Ethical Review Board (University of Glasgow) and carried out in accordance with the Animals (Scientific Procedures) Act 1986 and guidelines of the Committee of the National Cancer Research Institute. C57BL/6 mice were treated with ABT-737 (single intraperitoneal injection, 75 mg/kg and 125 mg/kg of ABT-737 or 3 consecutive days treatment of 75 mg/kg single dose per day), while control mice were treated with vehicle (30% v/v propylene glycol, 5% v/v Tween, 5% w/v dextrose, pH4). For a positive TUNEL staining control, mice were treated with doxorubicin (single intraperitoneal injection, 10 mg/kg). For tumourigenesis experiments, 7-week old CD1-*Nude* female mice were injected subcutaneously with 5×10^6 cells (15 mice per group). Tumor formation was monitored and tumor volume based on caliper measurements was calculated by the formula: tumor volume = (length x width x width)/2.

Supplemental References

Mathew, R., Karantza-Wadsworth, V., and White, E. (2009). Assessing metabolic stress and autophagy status in epithelial tumors. Methods in enzymology *453*, 53-81.

Rehm, M., Huber, H.J., Dussmann, H., and Prehn, J.H. (2006). Systems analysis of effector caspase activation and its control by X-linked inhibitor of apoptosis protein. The EMBO journal *25*, 4338-4349.

Rooswinkel, R.W., van de Kooij, B., de Vries, E., Paauwe, M., Braster, R., Verheij, M., and Borst, J. (2014). Antiapoptotic potency of Bcl-2 proteins primarily relies on their stability, not binding selectivity. Blood *123*, 2806-2815.

Shalem, O., Sanjana, N.E., Hartenian, E., Shi, X., Scott, D.A., Mikkelsen, T.S., Heckl, D., Ebert, B.L., Root, D.E., Doench, J.G., *et al.* (2014). Genome-scale CRISPR-Cas9 knockout screening in human cells. Science *343*, 84-87.

Stewart, S.A., Dykxhoorn, D.M., Palliser, D., Mizuno, H., Yu, E.Y., An, D.S., Sabatini, D.M., Chen, I.S., Hahn, W.C., Sharp, P.A., *et al.* (2003). Lentivirus-delivered stable gene silencing by RNAi in primary cells. Rna *9*, 493-501.

Zindy, F., Williams, R.T., Baudino, T.A., Rehg, J.E., Skapek, S.X., Cleveland, J.L., Roussel, M.F., and Sherr, C.J. (2003). Arf tumor suppressor promoter monitors latent oncogenic signals in vivo. Proceedings of the National Academy of Sciences of the United States of America *100*, 15930-15935.
